# Efficacy of Exercise‐Based Interventions for Metabolic Syndrome: An Umbrella Review With Meta‐Analyses

**DOI:** 10.1111/obr.70144

**Published:** 2026-04-26

**Authors:** Eric Tsz‐Chun Poon, Hong‐Yat Li, Po‐San Wong, Wesley Man‐Kuk Sum, Jonathan Peter Little, Angelo Sabag

**Affiliations:** ^1^ Department of Sports Science and Physical Education The Chinese University of Hong Kong Shatin Hong Kong; ^2^ School of Health and Exercise Sciences University of British Columbia Kelowna British Columbia Canada; ^3^ Sydney School of Health Sciences The University of Sydney Sydney New South Wales Australia; ^4^ Charles Perkins Centre The University of Sydney Sydney New South Wales Australia

**Keywords:** cardiometabolic health, overview, physical activity, public health

## Abstract

**Background:**

Regular exercise is a first‐line nonpharmacological strategy for managing metabolic syndrome (MetS), but varied exercise modalities and outcomes across studies have led to inconsistent findings that limit clinical guidance.

**Objective:**

To synthesize up‐to‐date evidence on the efficacy of exercise‐based interventions in improving MetS components and cardiometabolic health in individuals with MetS.

**Methods:**

Following the Preferred Reporting Items for Overviews of Reviews guideline, we searched seven databases from inception to May 2025 for systematic reviews with meta‐analyses evaluating various types of exercise‐based interventions (aerobic, high‐intensity interval training [HIIT], resistance, mind–body, and combined aerobic and resistance training). [Correction added on 1 May 2026, after first online publication: The previous sentence has been corrected in this version.] Outcomes included MetS components—waist circumference (WC), systolic/diastolic blood pressure (SBP/DBP), high‐density lipoprotein cholesterol (HDL‐C), triglycerides (TG), and fasting blood glucose (FBG)—along with other clinically relevant cardiometabolic parameters.

**Results:**

Twelve systematic reviews with meta‐analyses, representing 122 primary studies (9639 participants), were included. Overall, exercise‐based interventions significantly improved all MetS components and secondary cardiometabolic outcomes (all *p* < 0.001). Subgroup analyses showed aerobic and mind–body exercises improved all MetS components, whereas resistance, HIIT, and combined training enhanced specific components. Compared to usual care, combined training elicited larger effects on reducing FBG (−0.73 mmol/L, 95% CI: −1.43 to −0.02), TG (−0.26 mmol/L, 95% CI: −0.48 to −0.05), SBP (−4.25 mmHg, 95% CI: −7.16 to −1.34), and DBP (−3.69 mmHg, 95% CI: −5.18 to −2.20) than aerobic or resistance exercise alone.

**Conclusion:**

This umbrella review represents the largest evaluation specific to patients with MetS to date, indicating that exercise‐based interventions, across various modalities, significantly improve MetS components. The findings underscore the versatility of exercise, supporting tailored, patient‐centered prescriptions for managing MetS.

## 1 Introduction

Metabolic syndrome (MetS) represents a cluster of interconnected risk factors, including hyperglycemia, elevated blood pressure, dyslipidemia, and central obesity [1, 2]. Recent epidemiological research estimated that MetS affects approximately one in four adults globally, with prevalence varying by diagnostic criteria [3]. This represents a significant public health concern as the high MetS prevalence also heightens the likelihood of developing other chronic conditions and comorbidities, such as cardiovascular disease and type 2 diabetes mellitus [4]. Although the etiology of MetS is not fully understood, it is believed to be linked to a combination of genetic and lifestyle factors, including poor diet and a sedentary lifestyle, which can lead to obesity and insulin resistance—key components of MetS [5, 6]. Although pharmacological interventions exist for individual MetS components, there is growing recognition of the need for cost‐effective, accessible, and sustainable nonpharmacological approaches.

Physical activity (PA) has been widely recommended as a first‐line lifestyle intervention for the management of MetS and related conditions. Authoritative organizations such as the American College of Sports Medicine (ACSM) [7], Exercise and Sports Science Australia (ESSA) [8, 9], American Heart Association (AHA) [10], and World Health Organization (WHO) [11] have established guidelines that underscore the importance of regular PA in managing the condition. They generally recommend that all adults engage in a minimum of 150–300 min of moderate‐intensity PA or 75–150 min of vigorous‐intensity PA each week, or a combination of both, to improve health. However, these guidelines provide general PA recommendations but often lack specificity for exercise prescription. Moreover, global PA participation remains suboptimal [12], and the existing evidence base presents several critical gaps. First, existing reviews often focus on single modalities or components, lacking a holistic synthesis of their efficacy for managing MetS. Although some systematic reviews and meta‐analyses have examined specific exercise modalities including aerobic exercise [13, 14], resistance exercise [15], combined training (combining aerobic and resistance exercise) [16], mind–body exercise [17], or high‐intensity interval training (HIIT) [18, 19] on MetS, the relative efficacy of different exercise approaches remains unclear due to variability in study designs and outcome measures. Second, previous reviews have often focused on individual MetS components rather than the syndrome as a whole, potentially overlooking important interactions between metabolic parameters [14]. Third, the methodological quality and comprehensiveness of existing evidence syntheses vary considerably, creating uncertainty for clinical decision‐making [20]. These limitations pose challenges for healthcare professionals seeking to interpret the body of evidence regarding the impact of various exercise modalities on the overall cardiometabolic profile among the MetS cohort.

In this context, umbrella reviews—also known as overviews of reviews or meta‐reviews—have been proposed as a strategy to comprehensively synthesize evidence on a given topic [21]. Umbrella reviews represent the highest level of evidence synthesis by systematically evaluating and combining findings from multiple reviews. This approach offers three distinct advantages: (1) It provides a comprehensive overview of the entire evidence landscape, potentially offering a more reliable and comprehensive foundation for informing evidence‐based guidelines compared to individual systematic reviews [21]; (2) it uniquely integrates findings from multiple systematic reviews and meta‐analyses, which allows for a broader and more comparative analysis than a single meta‐analysis focused on one intervention or dataset; and (3) it enhances the statistical power to detect meaningful effects through large‐scale meta‐analyses. Their ability to synthesize the totality of highest quality evidence (systematic reviews) makes them an invaluable resource for researchers, clinicians, and allied healthcare professionals.

To the best of our knowledge, no umbrella review has been conducted to date examining exercise‐based interventions for MetS. Considering the rising prevalence and important healthcare implications of MetS, along with the substantial increase in relevant evidence published through systematic reviews and meta‐analyses in recent years, an umbrella review addressing the aforementioned research gaps to further establish the benefits and applications of various types of exercise‐based interventions among individuals with MetS appears timely. Furthermore, with the growing use of glucagon‐like peptide‐1 receptor agonists (GLP‐1RAs) for cardiometabolic management, exercise offers a unique and complementary nonpharmacological approach [22, 23]. Exercise interventions often provide distinct benefits in cardiometabolic health parameters, which can occur independently of weight loss [24, 25], highlighting their substantial therapeutic potential for individuals with MetS. Therefore, the primary aim of this review was to provide the most comprehensive synthesis of evidence to date by (1) quantifying the overall efficacy of exercise‐based interventions across all MetS core components and related cardiometabolic parameters, (2) comparing the relative effectiveness of different exercise modalities, and (3) critically appraising the methodological quality of existing evidence. The findings will provide crucial guidance for informing evidence‐based clinical practice while identifying key priorities for future research in MetS management.

## 2 Methods

### 2.1 Search Strategy

Our umbrella review of systematic reviews with meta‐analyses followed the Preferred Reporting Items for Overviews of Reviews (PRIOR) statement [21]. The protocol for the umbrella review was registered in the PROSPERO database (CRD420251058883). The review focused exclusively on peer‐reviewed systematic review articles published in English from inception until 1 May 2025. Seven databases (PubMed, EMBASE, Cochrane Database, CINAHL, Scopus, SPORTDiscus, and Web of Science) were searched using subject heading, keyword, and Medical Subject Headings (MeSH) term searches for “metabolic syndrome,” “physical activity,” “exercise,” “systematic review,” and “meta‐analysis” (detailed search strategy is presented in Table S1). The reference lists of the selected review articles were also examined for other potentially eligible papers.

### 2.2 Selection Procedure and Eligibility Criteria

The population, intervention, comparison, outcomes, and study type (PICOS) framework was used to develop the inclusion criteria.

#### 2.2.1 Populations

The population of interest was human participants who had been diagnosed with MetS based on recognized diagnostic criteria established by authoritative organizations (such as IFD, WHO, AHA/NHLBI, and NCEP‐ATP III) [1]. No exclusion criteria were applied to participants' age, sex, and baseline fitness. However, we excluded studies that combined MetS populations with other clinical groups unless the MetS subgroup data could be separately extracted.

#### 2.2.2 Interventions

Reviews that involved any type of exercise‐based intervention were included. Papers were eligible irrespective of exercise modality (e.g., aerobic exercise, resistance exercise, mind–body exercise, or other forms of exercise training), setting (e.g., laboratory, or community facility), or dose (e.g., weekly frequency and volume). Interventions had to last at least 2 weeks (regarded as reflecting the minimum time frame to observe meaningful changes in cardiometabolic health parameters in typical intervention studies) [26]. Studies involving dietary or other lifestyle interventions were only included if the intervention was used equally by all participants (i.e., including those in the comparator group).

#### 2.2.3 Comparator

Reviews that involved nonactive control comparison groups were included. Reviews with no comparison groups or those comparing with baseline values only were excluded.

#### 2.2.4 Outcomes

The primary outcomes for this umbrella review were MetS core risk components, including waist circumference (WC), systolic blood pressure (SBP), and diastolic blood pressure (DBP), high‐density lipoprotein cholesterol (HDL‐C), triglycerides (TG), and fasting blood glucose (FBG). Secondary outcome measures related to cardiometabolic health, including total body mass (BM), BM index (BMI), fat mass (FM), body fat percentage (BF%), cardiorespiratory fitness (Peak VO_2_), total cholesterol (TC), low‐density lipoprotein cholesterol (LDL‐C), homeostatic model assessment (HOMA‐IR), and MetS *z*‐score were also included in the analyses.

#### 2.2.5 Studies

Systematic reviews with meta‐analyses were selected.

### 2.3 Data Management and Extraction

Search results were imported into EndNote X10 (Clarivate, Philadelphia), where duplicates were removed. Two independent reviewers (E.P. and H.L.) conducted title/abstract and full‐text screening in duplicate. Interreviewer disagreements were resolved by consensus or arbitration by a third reviewer (P.W.). Data were extracted using a standardized extraction form, and two independent reviewers (E.P. and H.L.) performed the data extraction in duplicate. The extracted data included the lead author, year of publication, population characteristics, number of original studies, design of original studies, sample size, exercise intervention protocols, included outcomes, and main findings. Additional findings related to certainty of evidence, risk of bias (RoB), study quality, sensitivity analysis, publication bias, and potential moderators on exercise outcomes were extracted as supplementary information. Discrepancies were resolved through consensus or arbitration by a third reviewer (P.W.).

### 2.4 Methodological Quality Assessment of Included Systematic Reviews

Two independent reviewers (E.P. and W.S.) assessed the methodological quality of the included reviews in duplicate using the A MeaSurement Tool to Assess systematic Reviews (AMSTAR‐2) [20]. Discrepancies were resolved through consensus or arbitration by a third reviewer (P.W.). The AMSTAR‐2 consists of 16 items, each scored as “yes,” “partial yes,” or “no.” In this review, six items were considered “critical” because of their impact on evidence reliability, and 10 were considered “noncritical” [20]. The critical domains included protocol registration, adequacy of search strategy, risk of RoB assessment, appropriateness of meta‐analysis methods, use of RoB during interpretation, and assessment of publication bias. Reviews were rated as having “high confidence” (0 or 1 noncritical weakness), “moderate” (> 1 noncritical weakness but 0 critical flaws), “low” (1 critical flaw with or without noncritical weaknesses), or “critically low” (> 1 critical flaw with or without noncritical weaknesses) [20].

### 2.5 Umbrella Review Synthesis Methods

The overlap in component primary studies included in all eligible reviews was assessed using the corrected covered area (CCA) formula [27]: CCA = (*N* − *r*)/(*rc* − *r*), where *N* is the sum of total primary studies included in all the reviews, *r* is the number of unique primary studies, and *c* is the total number of reviews. The CCA ranges from 0% to 100%, with 100% indicating that all the reviews in an umbrella review included the same component original studies and 0% indicating that each review included entirely unique original studies. The CCA was categorized based on the following cutoffs: 0%–5% as “slight,” 6%–10% as “moderate,” 11%–15% as “high,” and > 15% as “very high” overlap [27].

Meta‐analysis results from each review reporting either standardized (i.e., standardized mean difference [SMD]) or unstandardized effect sizes (i.e., weighted mean difference [WMD] in absolute units) were presented using tables. SMD was calculated by dividing the difference in means between the intervention group and control group by the pooled standard deviation, whereas WMD was calculated by taking the difference in means between the intervention group and control group and weighting it by the inverse of the square root of the variance. Data reported by each review were cross‐checked with the original data reported by the primary studies for consistency. Aggregated results were summarized using mean ranges of effect sizes, as performed previously [28, 29].

### 2.6 Additional Meta‐Analyses Based on Primary Studies

To address the potential overlapping and methodological inconsistencies between individual reviews, we conducted additional meta‐analyses using eligible primary studies (i.e., randomized controlled trials [RCTs]) included in all reviews, as employed in prior research [30, 31]. Our analytical approach aligned with the guidance provided in the Cochrane Handbook for Systematic Reviews of Interventions [32]. The absolute change in mean difference and standard deviation of the outcome values from postintervention between groups in each study was calculated and pooled using the DerSimonian and Laird random‐effects method (RevMan Version 5.4.1; Cochrane Collaboration, Oxford, UK). SMDs with 95% CIs were used to synthesize continuous outcomes and create forest plots. To address the potential unit of analysis error, we followed the Cochrane Handbook's recommendation by combining all relevant exercise intervention groups (e.g., same type of exercise with varying volume and durations) and nonactive comparator groups into single groups within individual studies, creating a single pair‐wise comparison for the overall analysis [32]. Heterogeneity among studies was assessed using the chi‐square (Chi^2^) test, whereas the degree of inconsistency was quantified with the *I*‐square (*I* ^2^) statistic. *I* ^2^ values of < 25%, 50%, and 75% were considered indicative of low, moderate, and high heterogeneity, respectively [33]. Subgroup analyses were conducted based on the exercise modalities (aerobic exercise, resistance exercise, combined training, HIIT, and mind–body exercise) for outcomes with at least three studies in each comparison arm.

## 3 Results

### 3.1 Overview of Search Results

The search strategy yielded a total of 3962 records from seven electronic databases. After removing duplicates, 2808 records remained, out of which 2684 were subsequently excluded based on title and abstract screening, and eight reports were not retrieved. The full texts of the remaining 116 articles were assessed, and 12 systematic reviews and meta‐analyses that met the inclusion criteria were included in this umbrella review (refer to Figure 1 for flowchart and reasons for exclusions in Table S2).

**FIGURE 1:**
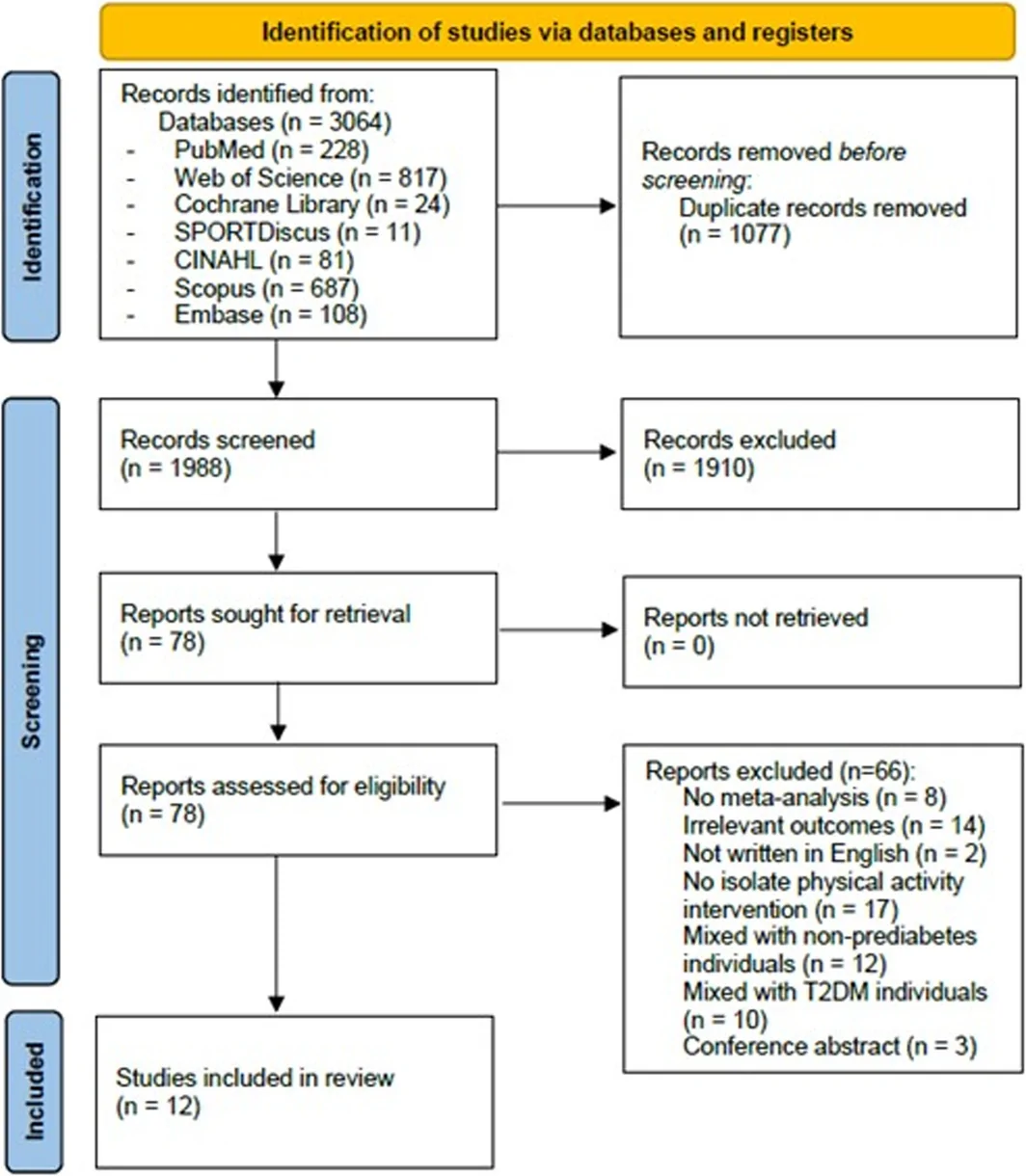
PRISMA 2020 flowchart of literature selection on systematic reviews.

### 3.2 Characteristics of Included Reviews

Table 1 presents a summary of the author, year, study type, population and intervention characteristics, outcomes, and main findings of the included systematic reviews. The sample sizes of the 12 systematic reviews ranged from 245 [35] to 2290 [14], with the number of studies in each review varying from 4 [34] to 44 [14]. A total of 122 unique primary studies with 9639 unique participants were listed in the included systematic reviews (Table S3), with a CCA of 3.68% indicating slight overlap. The publication year range of the primary studies was from 2013 to 2024. Two included reviews focused exclusively on aerobic exercise [13, 14], one on resistance exercise [15], three on HIIT [18, 19, 38], and three on mind–body exercise (including yoga and martial arts such as tai chi and qigong) [17, 34, 36]. Additionally, three reviews included multiple types of exercise interventions [16, 35, 37]. All systematic reviews consisted solely of RCTs, except one study [35] included a combination of RCTs and clinical controlled trials. Notably, most of the reviews concentrated on middle‐aged adults aged over 40 years. All reviews included studies with a minimum intervention period of 2 weeks and a frequency of 1–8.2 times per week, with each exercise session lasting between 10 and 90 min.

**TABLE 1:** Summary of included systematic reviews.

References	Included studies & sample size	Population	Exercise intervention	Outcome	Main findings from individual reviews
Cramer et al. 2016 [34]	*k* = 4 (RCTs)^a^ *n* = 450	Age: 48.2–62.4 (mean range) Gender: Male (*k* = 1), female (*k* = 1), and both (*k* = 2) Diagnostic method: NCEP (*k* = 2), revised NCEP (*k* = 1), and SAM‐NCEP (*k* = 1)	Type = mind–body exercise (yoga) Duration = 10–52 weeks Time = 60–90 min Frequency = 1–3 times/week, 2 times daily Adverse events = No (*k* = 1), NR (*k* = 3)	WC, TG, HDL‐C, FBG, SBP, and DBP	No recommendation can be made for or against yoga as an intervention for MetS.
Leme et al. 2018 [13]	*k* = 17 (RCTs) *n* = 495	Age: 43.1–64 (mean range) Gender: Male (*k* = 3), female (*k* = 2), and both (*k* = 12) Activity level: Inactive (*k* = 5), NR (*k* = 12) Diagnostic method: IDF, NECP, and WHO Diabetes status: Yes (*k* = 2), no (*k* = 5), NR (*k* = 10) Medication: NR (*k* = 11), no (*k* = 4), and restricted (*k* = 2)	Type = aerobic exercise Duration = 6–52 weeks Time = 25–60 min, 350–400 kcal/training session Frequency = 2–5 times/week	WC, TG, HDL‐C, FBG, SBP, and DBP	Aerobic exercise is effective in reducing blood pressure and WC; meanwhile, it increases HDL‐C level.
Leme et al. 2016 [15]	*k* = 8 (RCTs) *n* = 519	Age: 42.6–67.6 (mean range) Gender: Male (*k* = 3) and both (*k* = 5) Activity level: Sedentary or low level (*k* = 4), unclear (*k* = 2), and NR (*k* = 2) Diabetes status: Yes (*k* = 3), no (*k* = 2), and NR (*k* = 2) Medication: Restricted (*k* = 3, No glycemic control), and NR (*k* = 5)	Type = resistance exercise Duration = 12 weeks to 9 months Time = NR Frequency = 3 times/week	WC, TG, HDL‐C, FBG, SBP, and DBP	Resistance training would reduce systolic blood pressure, stroke mortality, and mortality from heart disease in people with MetS.
Li et al. 2024 [17]	*k* = 14 (RCTs) *n* = 1148	Age: 40–73 (range) Diagnostic method: NCEP (*k* = 5), IDF (*k* = 7), A (*k* = 1), and B (*k* = 1)	Type = Mind–body exercise (yoga, tai chi, fitness qigong: baduanjin, wuqinxim yijinjing) Duration = 10–48 weeks Time = 30–90 min Frequency = 2–7 times/week	WC, TG, HDL‐C, FBG, SBP, and DBP BMI and HOMA‐IR	Mind–body exercise is effective in improving MetS risk factors.
Ostman et al. 2017 [ 16 ]	*k* = 16 (RCTs) *n* = 1433	Age: NR Diagnostic method: IFD, WHO, and NCEP Diabetes status: Yes (*k* = 2) and NR (*k* = 15)	Type = aerobic exercise (*k* = 12), combined exercise (aerobic & resistance, *k* = 2), and aerobic and combined exercise (*k* = 2) Duration = 8–52 weeks Time = 30–150 min Frequency = 2–5 times/week	WC, TG, HDL‐C, FBG, SBP, DBP BMI, Peak VO_2_, BM, FM, and LDL	Exercise improves body composition, cardiovascular, and metabolic outcomes in people with MetS, whereas isolated aerobic training shows optimal outcomes.
Pattyn et al. 2013 [35]	*k* = 7 (5 RCTs & 2 CTs) *n* = 245	Age: 46–64 (range) Gender: both (*k* = 6) and female (*k* = 1) Diagnostic method: WHO, NCEP, and IDF Diabetes status: Yes (*k* = 1), no (*k* = 4), and NR (*k* = 2) Medication: Yes (*k* = 3, antihypertensive agents, lipid‐lowering agents, and oral hypoglycemic agents/insulin)	Type = aerobic exercise (*k* = 7), resistance exercise (*k* = 1), and combined (aerobic & resistance, *k* = 2) Duration = 8–52 weeks Time = 40–60 min Frequency = 2–5 times/week	WC, TG, HDL‐C, FBG, SBP, and DBP BMI, Peak VO_2_, BM, FM, TC, and LDL‐C	Dynamic endurance training has a favorable effect on most of the risk factors related to MetS.
Poon et al. 2024 [18]	*k* = 23 (RCTs) *n* = 1374	Age: 46–67 (mean range) Gender: Male (*k* = 2), female (*k* = 1), and both (*k* = 20) Activity level: No exclusion criteria applied Diagnostic method: WHO, IDF, AHA, and NCEP	Type = HIIT Duration = 2–24 weeks Time = 15–60 min Frequency = 3–5 times/week Adherence = most studies have a compliance level of ≥ 80% Adverse events = No (*k* = 12), NR (*k* = 11)	WC, TG, HDL‐C, FBG, SBP, DBP BMI, BM, FM, Peak VO_2_, TC, LDL‐C, HOMA‐IR, and MetS *z*‐score	HIIT improves cardiometabolic health in individuals with MetS. Low‐volume HIIT appears to be a viable alternative to traditional aerobic exercise.
Serrablo et al. 2020 [19]	*k* = 10 (RCTs) *n* = 529	Age: Adult Gender: Male (*k* = 2), female (*k* = 1), and both (*k* = 8) Diagnostic method: ≥ 3 MetS factors	Type = HIIT Duration = 3–24 weeks Time = 14–25 min Frequency = 3–5 times/week	WC, TG, HDL‐C, FBG, SBP, and DBP	HIIT improves FBG, SBP, DBP, and WC in people with MetS, compared to those who do not perform physical exercise.
Tao et al. 2023 [36]	*k* = 7 (RCTs) *n* = 496	Age: 40–70 (range)	Type = mind–body exercise (qigong) Duration = 12 weeks—6 months Time = 30–60 min Frequency = 4–7 times/week Adherence = 100% (*k* = 7)	WC, TG, HDL‐C, FBG, SBP, DBP BMI, LDL‐C, and TC	Qigong may be an alternative exercise to improve cardiovascular risk factors among people with MetS.
Wewege et al. 2018 [37]	*k* = 12 (RCTs) *n* = 608	Age: 51 (mean); 38–60 (range) Gender: Male (*k* = 1), female (*k* = 5), and both (*k* = 6) Diagnostic method: IDF (*k* = 7), NCEP (*k* = 7), and syndrome X (*k* = 1) Medication: Yes (*k* = 4, hypertensive medication, no restriction), no (*k* = 5), NR (*k* = 3), and restricted (*k* = 3)	Type = Aerobic exercise (*k* = 9), Resistance exercise (*k* = 2) Duration = 8–52 weeks Time = 20–60 min Frequency = 3–5 times/week	WC, TG, HDL‐C, FBG, SBP, and DBP BM, BMI, FM, LDL‐C, and TC	Aerobic exercise following current guidelines provided widespread benefits to people with MetS.
Wood et al. 2021 [14]	*k* = 44 (RCTs) *n* = 2990	Age: < 35: *k* = 1, 35–55: *k* = 25, > 55: *k* = 22 Gender: Male (*k* = 8), female (*k* = 8), and both (*k* = 28) Activity level: Sedentary Diagnostic method: ≥ 3 MetS factors	Type = aerobic exercise Duration = 12–104 weeks Time = 15–90 min Frequency = 1–8.2 times/week	TG, HDL‐C, LDL‐C, and TC	Aerobic exercise training positively changes the standard lipid profile.
Yin et al. 2024 [38]	*k* = 5 (RCTs)^a^ *n* = 268	Age: 48.8–56.7 (mean range) NR (*k* = 1) Gender: Both (*k* = 3) and NR (*k* = 2) Diabetes status: No	Type = HIIT (low volume) Duration = 12–16 weeks Time = 10–14 min Frequency = 2–3 times/week	MetS *z*‐score	LV‐HIIT was efficacious in improving MetS *z*‐score in nonathlete adults.

Abbreviations: A: Diabetes Branch of the Chinese Medical Association in 2004; AHA: America Heart Association; B: Guidelines for the Prevention and Control of Type 2 Diabetes Mellitus in China in 2017; BF%: body fat percentage; BM: body mass; BMI: body mass index; DBP: diastolic blood pressure; FBG: fasting blood glucose; FM: fat mass; HDL‐C: high‐density lipoprotein cholesterol; HIIT: high‐intensity interval training; HOMA‐IR: Homeostatic Model Assessment for Insulin Resistance; IDF: International Diabetes Federation metabolic syndrome criteria; *k*: number of studies; LDL‐C: low‐density lipoprotein cholesterol; MetS: metabolic syndrome; MICT: moderate‐intensity interval training; NCEP: US National Cholesterol Education Program Adult Treatment Panel III; SBP: systolic blood pressure; TC: total cholesterol; TG: triglyceride; WC: waist circumference.

^a^ Only select the relevant RCTs involved in MA.

### 3.3 Summary of Meta‐Analyses Based on Included Reviews

The cardiometabolic outcomes included in each systematic review are summarized in Table 1, with Table S4 providing a comprehensive summary of the meta‐analysis results from all 12 systematic reviews. In comparison with nonactive control conditions, our included reviews generally showed beneficial effects of exercise‐based interventions on improving five core MetS components, including WC (Figure 2a), SBP (Figure 2b), DBP (Figure 2c), HDL‐C (Figure 2d), and TG (Figure 2e), although several individual reviews displayed relatively wide CIs that crossed 0. Additionally, FBG showed improvements following exercise‐based interventions in most reviews (Figure 2f), except for one review focused on resistance exercise [37] and another on mind–body yoga exercise [34]. Regarding other parameters of cardiometabolic health, all included reviews consistently revealed beneficial impacts following exercise‐based interventions as shown in Table S4, which included BMI, BM, FM, HOMA‐IR, TC, LDL‐C, Peak VO_2_, and MetS *z*‐score.

**FIGURE 2:**
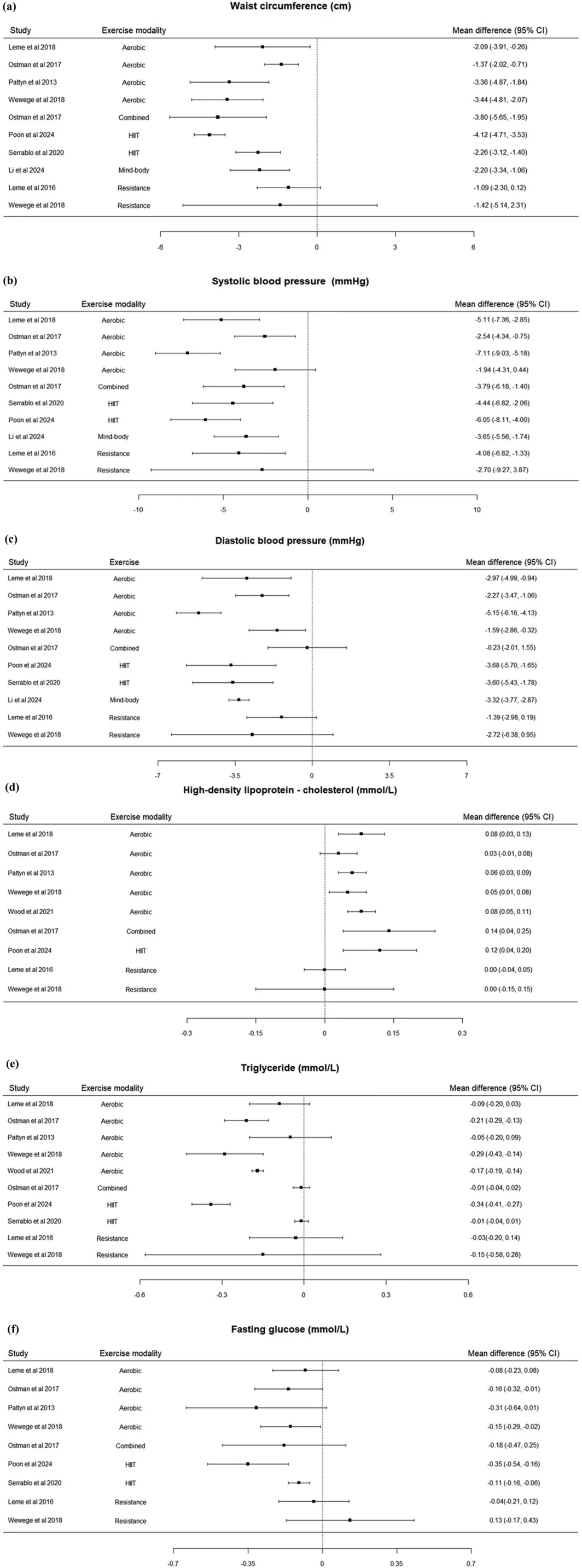
Summary of meta‐analysis results from included reviews that compared exercise‐based interventions with nonactive control conditions for (a) WC, (b) SBP, (c) DBP, (d) HDL‐C, (e) TG, and (f) FG (expressed in WMD with 95% CI).

With regard to moderation analysis, nine out of 12 reviews in this umbrella review explicitly examined the potential moderators of exercise effects. These investigations were typically conducted through subgroup analysis or linear meta‐regression analysis. However, because of the relatively small sample sizes, lack of detailed information, and methodological limitations in the original studies included, three systematic reviews [19, 34, 35] were unable to perform quantitative analyses for potential moderators of exercise effects. Nonetheless, five systematic reviews [14, 15, 17, 36, 37] suggested that interventions lasting ≥ 3–6 months may confer greater advantages in improving MetS core components and/or other cardiometabolic health outcomes compared with shorter interventions. Additionally, several reviews reported that other intervention components including exercise intensity [14, 37], volume [14], and frequency [17, 37], as well as participants' age [37] and gender [37], may moderate the effects of exercise, although other reviews did not observe clear moderation effects on these parameters.

### 3.4 Additional Meta‐Analyses Based on Primary Studies

To address potential overlaps among primary studies and methodological inconsistencies between individual reviews, additional meta‐analyses were conducted using eligible primary studies from all reviews. This approach aimed to enhance the certainty of the findings and facilitate comparisons regarding the relative effectiveness of different exercise modalities (see Table 2 for a tabulated summary). Overall, our meta‐analyses indicated that exercise‐based interventions significantly improved all MetS core components (all *p* < 0.001), including WC (*k* = 93; WMD: −2.94 cm; 95% CI: −3.54 to −2.34), SBP (*k* = 98; WMD: −4.16 mmHg; 95% CI: −7.11 to −1.94), DBP (*k* = 98; WMD: −2.58 mmHg; 95% CI: −3.12 to −2.05), HDL‐C (*k* = 118; WMD: 0.08 mmol/L; 95% CI: 0.05 to 0.10), TG (*k* = 115; WMD: −0.19 mmol/L; 95% CI −0.23 to −0.15), and FBG (*k* = 107; WMD: −0.37 mmol/L; 95% CI: −0.48 to −0.26). Other clinically relevant parameters of cardiometabolic health also showed consistent improvement trends (all *p* < 0.001), including BMI (*k* = 80; WMD: −0.95 kg/m^2^; 95% CI: −1.21 to −0.69), BM (*k* = 80; WMD: −2.24 kg; 95% CI: −4.84 to −0.20), FM (*k* = 36; WMD: −0.73 kg; 95% CI: −1.05 to −0.41), BF% (*k* = 21; WMD: −2.21%; 95% CI: −3.23 to −1.19), HOMA‐IR (*k* = 27; WMD: −0.56 unit; 95% CI: −0.81 to −0.32), TC (*k* = 92; WMD: −0.25 mmol/L; 95% CI: −0.32 to −0.18), LDL‐C (*k* = 85; WMD: −0.18 mmol/L; 95% CI: −0.24 to −0.12), Peak VO_2_ (*k* = 63; WMD: 3.68 mL/kg/min; 95% CI: 2.77 to 4.60), and MetS *z*‐score (*k* = 17; WMD: −0.31 unit; 95% CI: −0.36 to −0.26).

**TABLE 2:** Summary of meta‐analyses comparing various types of exercise‐based interventions for MetS based on primary studies.

Outcome	*k*	Participants	Mean difference (95% CI)	*p*	Tau^2^	Chi^2^ (*p*)	*I* ^2^
Primary outcome measures: MetS core risk factors							
Waist circumference (cm)	93	5540	−2.94 (−3.54 to −2.34)	< 0.001	3.75	329.3 (*p* < 0.001)	72%
High‐intensity interval training	19	966	−2.96 (−3.90 to −2.02)	< 0.001	0.02	18.1 (*p* = 0.45)	0%
Resistance exercise	10	412	−2.38 (−4.31 to −0.46)	0.02	3.73	21.3 (*p* = 0.01)	58%
Aerobic exercise	40	2102	−2.86 (−3.83 to −1.89)	< 0.001	4.68	203.7 (*p* < 0.001)	81%
Combined training	7	755	−2.85 (−4.19 to −1.50)	< 0.001	0.94	9.0 (*p* = 0.18)	33%
Mind–body exercise	14	1256	−2.91 (−4.23 to −1.59)	< 0.001	3.87	46.1 (*p* < 0.001)	72%
Systolic blood pressure (mmHg)	98	5636	−4.16 (−5.13 to −3.19)	< 0.001	11.40	423.5 (*p* < 0.001)	77%
High‐intensity interval training	17	839	−5.23 (−7.37 to −3.09)	< 0.001	9.12	41.9 (*p* < 0.001)	62%
Resistance exercise	13	503	−2.09 (−7.20 to 3.03)	0.42	67.04	98.4 (*p* < 0.001)	88%
Aerobic exercise	43	2312	−3.57 (−5.17 to −1.98)	< 0.001	13.53	155.8 (*p* < 0.001)	73%
Combined training	7	749	−4.25 (−7.16 to −1.34)	0.004	5.35	10.8 (*p* = 0.10)	44%
Mind–body exercise	16	1191	−5.35 (−7.32 to −3.38)	< 0.001	8.04	35.8 (*p* = 0.002)	58%
Diastolic blood pressure (mmHg)	98	5501	−2.58 (−3.12 to −2.05)	< 0.001	2.27	208.6 (*p* < 0.001)	53%
High‐intensity interval training	17	823	−3.20 (−4.44 to −1.97)	< 0.001	1.28	20.8 (*p* = 0.19)	23%
Resistance exercise	13	503	−1.57 (−2.18 to −0.95)	< 0.001	0.00	6.3 (*p* = 0.90)	0%
Aerobic exercise	44	2312	−2.18 (−3.11 to −1.25)	< 0.001	3.87	106.5 (*p* < 0.001)	60%
Combined training	7	369	−3.69 (−5.18 to −2.20)	< 0.001	1.27	9.9 (*p* = 0.13)	40%
Mind–body exercise	15	1056	−3.38 (−4.77 to −1.99)	< 0.001	3.78	35.3 (*p* = 0.001)	60%
High‐density lipoprotein cholesterol (mmol/L)	118	7169	0.08 (0.05 to 0.10)	< 0.001	0.01	1184.4 (*p* < 0.001)	90%
High‐intensity interval training	20	1040	0.05 (−0.07 to 0.16)	0.41	0.06	558.1 (*p* < 0.001)	97%
Resistance exercise	13	546	0.06 (0.01 to 0.11)	0.02	0.00	35.6 (*p* < 0.001)	66%
Aerobic exercise	56	3285	0.08 (0.05 to 0.11)	< 0.001	0.01	260.4 (*p* < 0.001)	79%
Combined training	9	819	0.03 (−0.04 to 0.10)	0.35	0.01	60.7 (*p* < 0.001)	87%
Mind–body exercise	16	1351	0.12 (0.06 to 0.18)	< 0.001	0.01	121.3 (*p* < 0.001)	88%
Triglycerides (mmol/L)	115	6935	−0.19 (−0.23 to −0.15)	< 0.001	0.02	288.7 (*p* < 0.001)	61%
High‐intensity interval training	20	1040	−0.23 (−0.32 to −0.14)	< 0.001	0.01	38.6 (*p* = 0.005)	51%
Resistance exercise	13	509	−0.16 (−0.31 to −0.02)	0.03	0.02	22.2 (*p* = 0.04)	46%
Aerobic exercise	54	3059	−0.17 (−0.22 to −0.12)	< 0.001	0.01	87.7 (*p* = 0.002)	40%
Combined training	9	819	−0.26 (−0.48 to −0.05)	0.02	0.04	15.4 (*p* = 0.05)	48%
Mind–body exercise	16	1406	−0.35 (−0.51 to −0.18)	< 0.001	0.06	47.1 (*p* < 0.001)	68%
Fasting blood glucose (mmol/L)	107	6315	−0.37 (−0.48 to −0.26)	< 0.001	0.21	1394.3 (*p* < 0.001)	92%
High‐intensity interval training	19	966	−0.30 (−0.62 to 0.03)	0.07	0.36	123.6 (*p* < 0.001)	85%
Resistance exercise	12	442	−0.30 (−0.61 to 0.02)	0.07	0.21	57.5 (*p* < 0.001)	81%
Aerobic exercise	48	2529	−0.39 (−0.57 to −0.21)	< 0.001	0.25	649.4 (*p* < 0.001)	93%
Combined training	7	705	−0.73 (−1.43 to −0.02)	0.04	0.52	57.0 (*p* < 0.001)	89%
Mind–body exercise	18	1571	−0.41 (−0.63 to −0.19)	< 0.001	0.14	145.2 (*p* < 0.001)	88%
Secondary outcome measures: Other clinically relevant cardiometabolic health variables							
Low‐density lipoprotein cholesterol (mmol/L)	85	5582	−0.18 (−0.24 to −0.12)	< 0.001	0.04	496.8 (*p* < 0.001)	83%
High‐intensity interval training	13	628	−0.21 (−0.35 to −0.06)	0.004	0.03	37.9 (*p* < 0.001)	68%
Resistance exercise	10	459	−0.21 (−0.38 to −0.04)	0.02	0.03	16.3 (*p* = 0.06)	45%
Aerobic exercise	42	2711	−0.14 (−0.22 to −0.06)	< 0.001	0.03	117.9 (*p* < 0.001)	65%
Combined training	6	764	−0.21 (−0.69 to 0.27)	0.39	0.03	118.7 (*p* < 0.001)	96%
Mind–body exercise	12	962	−0.30 (−0.50 to −0.11)	0.002	0.08	172.2 (*p* < 0.001)	94%
Total cholesterol (mmol/L)	92	5416	−0.25 (−0.32 to −0.18)	< 0.001	0.05	397.6 (*p* < 0.001)	77%
High‐intensity interval training	14	612	−0.30 (−0.45 to −0.15)	< 0.001	0.04	33.8 (*p* = 0.001)	62%
Resistance exercise	11	410	−0.21 (−0.46 to 0.05)	0.12	0.10	32.1 (*p* < 0.001)	69%
Aerobic exercise	42	2476	−0.16 (−0.26 to −0.07)	< 0.001	0.04	126.3 (*p* < 0.001)	68%
Combined training	9	819	−0.36 (−0.73 to 0.01)	0.06	0.18	68.5 (*p* < 0.001)	88%
Mind–body exercise	13	997	−0.49 (−0.75 to −0.23)	< 0.001	0.16	59.5 (*p* < 0.001)	80%
Homeostatic Model Assessment for Insulin Resistance (HOMA‐IR)	27	1671	−0.56 (−0.81 to −0.32)	< 0.001	0.21	102.1 (*p* < 0.001)	75%
High‐intensity interval training	5	288	−0.90 (−1.60 to −0.21)	0.01	0.49	29.3 (*p* < 0.001)	86%
Aerobic exercise	15	587	−0.13 (−0.34 to 0.08)	0.23	0.00	11.6 (*p* = 0.64)	0%
Combined training	3	611	−0.67 (−1.40 to 0.07)	0.08	0.30	11.0 (*p* = 0.004)	82%
Body mass index (kg/m^2^)	80	4669	−0.95 (−1.21 to −0.69)	< 0.001	0.51	267.2 (*p* < 0.001)	70%
High‐intensity interval training	14	721	−1.29 (−2.02 to −0.56)	< 0.001	0.45	18.6 (*p* = 0.14)	30%
Resistance exercise	8	248	−1.05 (−2.27 to 0.18)	0.09	1.47	16.3 (*p* = 0.02)	57%
Aerobic exercise	36	1921	−0.82 (−1.21 to −0.42)	< 0.001	0.58	91.61 (*p* < 0.001)	62%
Combined training	7	686	−0.74 (−1.39 to −0.09)	0.02	0.25	11.4 (*p* = 0.08)	47%
Mind–body exercise	12	991	−1.45 (−2.11 to −0.78)	< 0.001	0.85	46.5 (*p* < 0.001)	76%
Body mass (kg)	80	3945	−2.28 (−3.19 to −1.37)	< 0.001	5.85	354.8 (*p* < 0.001)	83%
High‐intensity interval training	14	718	−2.52 (−4.84 to −0.20)	0.03	6.63	29.29 (*p* = 0.0006)	56%
Resistance exercise	12	404	−1.26 (−3.17 to 0.65)	0.20	4.35	30.7 (*p* = 0.001)	64%
Aerobic exercise	37	1778	−2.91 (−4.20 to −1.63)	< 0.001	6.98	193.9 (*p* < 0.001)	81%
Combined training	7	171	−0.79 (−3.91 to 2.33)	0.62	7.31	19.1 (*p* = 0.004)	69%
Mind–body exercise	8	772	−1.92 (−3.59 to −0.25)	0.02	2.10	11.6 (*p* = 0.12)	40%
Fat mass (kg)	36	1514	−0.73 (−1.05 to −0.41)	< 0.001	0.09	41.9 (*p* = 0.20)	16%
High‐intensity interval training	9	503	−1.56 (−3.70 to 0.59)	0.16	0.00	5.0 (*p* = 0.76)	0%
Resistance exercise	6	245	−0.02 (−0.64 to 0.60)	0.96	0.00	3.0 (*p* = 0.70)	0%
Aerobic exercise	15	610	−1.65 (−2.60 to −0.69)	< 0.001	1.01	26.0 (*p* = 0.03)	46%
Combined training	3	54	−0.72 (−0.99 to −0.45)	< 0.001	0.00	0.1 (*p* = 0.96)	0%
Fat percentage (%)	21	675	−2.21 (−3.23 to −1.19)	< 0.001	2.17	58.7 (*p* = 0.20)	66%
High‐intensity interval training	3	154	−3.34 (−7.74 to 1.06)	0.14	11.05	7.6 (*p* = 0.02)	74%
Resistance exercise	4	83	−2.15 (−6.66 to 2.36)	0.35	11.80	7.3 (*p* = 0.06)	59%
Aerobic exercise	12	389	−2.04 (−3.22 to −0.85)	< 0.001	1.83	37.7 (*p* < 0.001)	71%
Peak VO_2_ (mL/kg/min)	63	3223	3.68 (2.77 to 4.60)	< 0.001	8.52	349.4 (*p* < 0.001)	82%
High‐intensity interval training	15	784	4.37 (1.65 to 7.09)	0.002	23.02	193.9 (*p* < 0.001)	93%
Resistance exercise	7	193	2.40 (1.20 to 3.61)	< 0.001	0.00	1.7 (*p* = 0.94)	0%
Aerobic exercise	30	1323	3.20 (2.45 to 3.95)	< 0.001	1.45	48.7 (*p* = 0.01)	40%
Combined training	6	666	3.36 (1.79 to 4.94)	< 0.001	0.65	5.7 (*p* = 0.34)	12%
Metabolic syndrome severity *z*‐score (MetS *z*‐score)	17	1017	−0.31 (−0.36 to −0.26)	< 0.001	0.00	12.3 (*p* = 0.72)	0%
High‐intensity interval training	9	549	−0.32 (−0.37 to −0.27)	< 0.001	0.00	4.4 (*p* = 0.82)	0%

Subgroup analyses revealed differential effects of exercise modalities on MetS parameters (see Table 2). Both aerobic and mind–body exercises demonstrated significant improvements (*p* < 0.05) across all six MetS components. Resistance exercise significantly benefited WC, DBP, HDL‐C, and TG (*p* < 0.05), whereas HIIT showed significant improvements in WC, SBP, DBP, and TG (*p* < 0.001). Combined training significantly improved WC, SBP, DBP, TG, and FBG (*p* < 0.05) but not HDL‐C. Of note, combined training showed greater effect sizes in reducing FBG (WMD: −0.73 mmol/L, 95% CI: −1.43 to −0.02), TG (WMD: −0.26 mmol/L, 95% CI: −0.48 to −0.05), SBP (WMD: −4.25 mmHg, 95% CI: −7.16 to −1.34), and DBP (WMD: −3.69 mmHg, 95% CI: −5.18 to −2.20) compared to aerobic or resistance exercise alone (Table 2).

Regarding the subgroup analyses of secondary cardiometabolic health outcomes (Table 2), the results were more equivocal, potentially because of limited primary studies (i.e., *k* < 10) for certain modality–parameter combinations. Nonetheless, all exercise modalities appeared to confer additional benefits across various cardiometabolic health markers, beyond their effects on core MetS parameters. The detailed results are provided in Table 2.

### 3.5 Methodological Quality of Included Reviews

Table 3 provides a summary of the AMSTAR‐2 scores. Two reviews received a moderate score, whereas five reviews received a low score, and five received a critically low score (see File S1 for scoring justifications). Specifically, only five (42%) of the reviews fully referred to a predefined methodology (Item 2). None of the studies reported on the sources of funding for the included studies (Item 10), and only four of the studies (33%) provided a list of excluded studies with reasons for exclusions (Item 7). Furthermore, four reviews (33%) did not employ appropriate methods for the statistical combination of meta‐analysis results (Item 11). Only seven reviews (58%) fully used a satisfactory technique for assessing the RoB in individual studies (Item 9), and six (50%) assessed the potential impact of RoB on the results (Item 12). Seven reviews (58%) discussed heterogeneity in the results (Item 14) and investigated publication bias (Item 15) when conducting meta‐analyses. Results of certainty of evidence using Grading of Recommendations Assessment, Development and Evaluation (GRADE) and RoB reported by included reviews were provided in Table S5. Out of the 12 included original reviews, only two reviews provided GRADE assessments for all MetS risk components [13, 15]. The reported certainty of outcomes for WC, SBP, DBP, HDL‐C, and TG ranged from “low” to “moderate,” whereas the certainty for FBG was assessed as “low.”

**TABLE 3:** AMSTAR‐2 ratings of systematic reviews and meta‐analyses.

Reference	1	2	3	4	5	6	7	8	9	10	11	12	13	14	15	16	Confidence
Cramer et al. [34]	Y	N	Y	PY	Y	Y	Y	Y	Y	N	N	Y	Y	N	N	Y	Critically low
Leme et al. [13]	Y	PY	Y	PY	Y	N	N	PY	PY	N	Y	Y	Y	Y	Y	Y	Moderate
Leme et al. [15]	Y	PY	Y	PY	Y	Y	Y	PY	Y	N	Y	Y	Y	Y	N	Y	Low
Li et al. [17]	Y	Y	Y	PY	Y	Y	N	Y	Y	N	N	N	N	N	Y	Y	Critically low
Ostman et al. [ 16 ]	Y	Y	Y	PY	Y	Y	N	Y	Y	N	Y	N	N	N	Y	Y	Low
Pattyn et al. [35]	N	N	Y	N	N	Y	Y	N	PY	N	N	N	N	N	Y	Y	Critically low
Poon et al. [18]	Y	Y	Y	PY	Y	Y	N	Y	Y	N	Y	Y	Y	Y	Y	Y	Moderate
Serrablo et al. [19]	Y	N	Y	PY	Y	N	N	Y	PY	N	N	N	N	N	N	Y	Critically low
Tao et al. [36]	Y	N	Y	N	N	N	N	Y	Y	N	Y	Y	Y	Y	N	N	Critically low
Wewege et al. [37]	Y	Y	Y	PY	Y	N	Y	N	PY	N	Y	N	N	Y	Y	Y	Low
Wood et al. [14]	Y	Y	Y	PY	Y	Y	N	N	Y	N	Y	Y	Y	Y	N	Y	Low
Yin et al. [38]	Y	PY	Y	PY	N	Y	N	N	PY	N	Y	N	N	Y	Y	Y	Low

*Note:* ITEM—DESCRIPTION 1 Did the research questions/inclusion criteria include the components of PICO? 2 Did the review contain an explicit statement that the review methods were established prior to the conduct of the review? 3 Did the review authors explain their selection of the study designs for inclusion in the review? 4 Did the review authors use a comprehensive literature search strategy? 5 Did the review authors perform study selection in duplicate? 6 Did the review authors perform data extraction in duplicate? 7 Did the review authors provide a list of excluded studies and justify the exclusions? 8 Did the review authors describe the included studies in adequate detail? 9 Did the review authors assess the RoB in studies that were included in the review? 10 Did the review authors report on the sources of funding for the studies included in the review? 11 If a meta‐analysis was performed, did the review authors use appropriate methods for statistical combination of results? 12 If meta‐analysis was performed, did the review authors assess the potential impact of RoB in individual studies on the results of the meta‐analysis? 13 Did the review authors account for RoB in individual studies when interpreting the results of the review? 14 Did the review authors provide a satisfactory explanation for, and discussion of, any heterogeneity observed in the results of the review? 15 If they performed quantitative synthesis, did the review authors investigate publication bias? 16 Did the review authors report any potential sources of conflict of interest, including any funding they received for conducting the review?

Abbreviations: N: no; PY: partial yes; Y: yes.

## 4 Discussion

To the best of our knowledge, this is the first umbrella review evaluating the efficacy of exercise‐based interventions in individuals with MetS. Synthesizing 12 systematic reviews with meta‐analyses, encompassing 122 primary studies and 9639 unique participants, our findings establish exercise as a pivotal nonpharmacological strategy for managing MetS (see Figure 3 for the graphical representation of findings). Our meta‐analyses demonstrate that exercise‐based interventions overall significantly improve all MetS components—WC, SBP, DBP, HDL‐C, TG, and FBG. Secondary outcomes, including BMI, FM, HOMA‐IR, and Peak VO_2_, also exhibited consistent improvements. These findings align with guidelines from major health organizations advocating exercise for cardiometabolic risk reduction [7, 10, 11], reinforcing exercise's broad therapeutic potential. Our results, robustly supported by additional meta‐analyses addressing slight study overlaps (CCA: 3.68%) and methodological inconsistencies, underscore the versatility of exercise modalities in addressing MetS's multifaceted risk profile.

**FIGURE 3:**
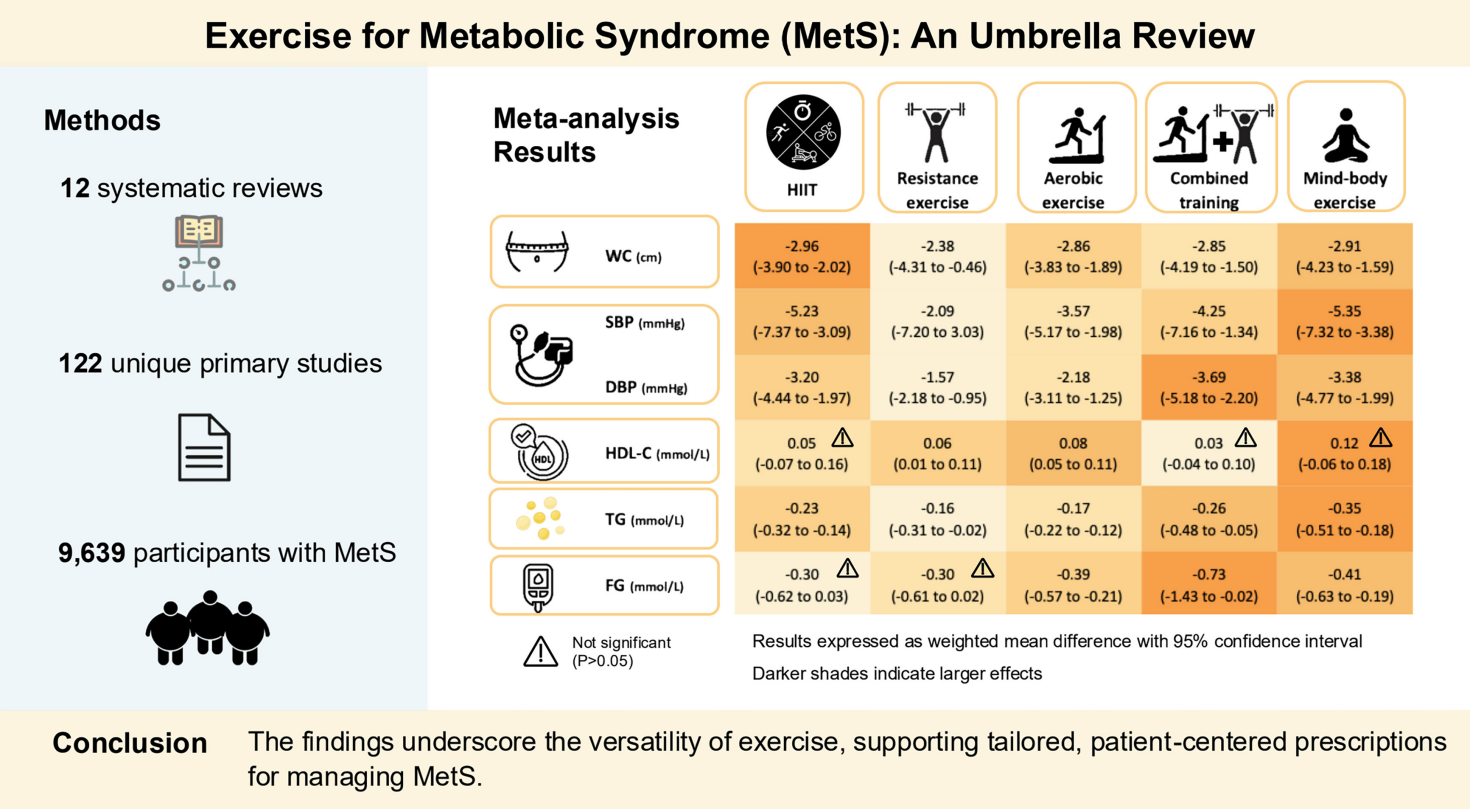
Graphical representation of the efficacy of exercise‐based intervention in improving MetS components. DBP: diastolic blood pressure, FBG: fasting blood glucose, HDL‐C: high‐density lipoprotein cholesterol, HIIT: high‐intensity interval training, SBP: systolic blood pressure, TG: triglyceride, WC: waist circumference. [Correction added on 1 May 2026, after first online publication: Figure 3 has been updated in this version.]

### 4.1 Modality‐Specific Effects and Mechanisms in Exercise Interventions for MetS

A highlighted aspect of the present umbrella review is the comparative analysis of modality‐specific effects, offering insights for tailored exercise interventions with MetS. As shown in Table 2, our results suggest differential effects on MetS components across modalities, which are possibly driven by complementary yet distinct physiological mechanisms underlying their cardiometabolic benefits. Specifically, aerobic exercise improved all MetS components. It is well‐documented for promoting cardiorespiratory fitness and sustained energy expenditure [39], as well as improving insulin sensitivity via increased skeletal muscle GLUT4 expression [40]. These pathways likely explain its robust effect on FBG (−0.39 mmol/L) and Peak VO_2_ (3.20 mL/kg/min). It also enhances endothelial function via nitric oxide production, lowering SBP (−3.57 mmHg) and DBP (−2.18 mmHg) [41, 42]. Further, heightened fat oxidation mediated by lipolytic hormones may drive reductions in abdominal fat (WC: −2.86 cm) [43] and improvements in lipid metabolism (HDL‐C: 0.08 mmol/L; TG: −0.17 mmol/L) [44, 45]. Resistance exercise is proficient in increasing muscle mass and function, associated with enhanced glucose uptake capacity, insulin sensitivity, and lipid metabolism [46]. These changes may account for its significant effects on WC (−2.38 cm), HDL‐C (0.06 mmol/L), and TG (−0.16 mmol/L). Its effect on DBP (−1.57 mmHg) may reflect improved vascular compliance [47], but its nonsignificant, limited impact on SBP may stem from lower cardiovascular demand compared to aerobic exercise. Combined training leverages aerobic‐driven fat oxidation and resistance‐driven muscle glucose uptake [48]. This comprehensive nature may explain its larger effect sizes in improving SBP (−4.25 mmHg), DBP (−3.69 mmHg), TG (−0.26 mmol/L), and FBG (−0.73 mmol/L) when compared to aerobic or resistance exercise alone. However, its lack of effect on HDL‐C may reflect competing metabolic demands [49] or insufficient aerobic intensity to optimize lipid metabolism compared to single‐component exercise.

With short bursts of intense exercise followed by brief recovery periods, HIIT is purported to rapidly induce hormonal changes and excess postexercise oxygen consumption (EPOC) [50, 51]. HIIT is known to enhance mitochondrial biogenesis and GLUT4 translocation, improving insulin sensitivity and lipid metabolism [50, 51]. These metabolic changes may drive its significant effects on WC (−2.96 cm), TG (−0.23 mmol/L), and blood pressure (SBP: −5.23 mmHg; DBP: −3.20 mmHg) despite the relatively low energy cost of this approach when compared to traditional higher volume exercise. Yet, its nonsignificant effect on FBG and HDL‐C may result from shorter intervention durations or variability in protocols (e.g., work: rest ratio, intensity, and volume) [18]. Mind–body exercise (e.g., yoga and tai chi) combines moderate activity with stress reduction techniques, which may reduce systemic inflammation, a key contributor to MetS, by decreasing basal levels of pro‐inflammatory cytokines (e.g., TNF‐α, C‐reactive protein, and IL‐6) [52, 53]. Their anti‐inflammatory and stress‐reducing effects may contribute to their broad efficacy across all MetS components (WC: −2.91 cm; SBP: −5.35 mmHg; DBP: −3.38 mmHg; HDL‐C: 0.12 mmol/L; TG: −0.35 mmol/; FBG: −0.41 mmol/L) [54]. Collectively, these modality‐specific mechanisms, while sharing some common pathways, differ in their primary physiological targets, contributing to the varied effects on MetS components and reinforcing the value of tailored exercise prescriptions in MetS management.

### 4.2 Methodological Issues Identified From Included Reviews

Despite the robust evidence synthesized from our current work, we identified several methodological issues that future systematic reviews should address. Firstly, it is noted that out of the 12 original reviews included, only two provided explicit GRADE assessments. The certainty of the outcomes reported in both reviews was limited to six MetS risk components (e.g., WC, SBP, DBP, HDL‐C, TG, and FBG) and showed a diverse range of judgments, varying from “low” to “moderate.” These observations suggest a lack of robust evaluation across individual reviews, which may affect the interpretations of the certainty of evidence across specific cardiometabolic parameters. Secondly, a relatively high proportion of included systematic reviews were rated as low (*k* = 5) or critically low (*k* = 5) in quality based on the AMSTAR‐2 rating, and did not strictly adhere to the Preferred Reporting Items for Systematic Reviews and Meta‐Analysis (PRISMA) guidelines, which currently present a widely accepted standard for reporting meta‐analysis. For instance, Li et al. [17] and Serrablo‐Torrejon et al. [19] inaccurately reported their meta‐analysis results as standardized effect size (SMD) in the text, whereas the actual synthesis was conducted in absolute units (mean difference; MD), as indicated by the forest plots provided by the authors. Reporting MD with the corresponding units is preferred for ease of interpretation and offers a more practical understanding of the therapeutic effect, particularly when assessing the same health outcome using consistent measurement units and comparable methodologies [32]. The erroneous use of SMD in place of MD could mislead readers unfamiliar with meta‐analytic techniques and result in incorrect interpretations of the findings. Future studies should exercise greater diligence in selecting and reporting effect measures to ensure accuracy and clarity in meta‐analytic research in accordance with existing guidelines [32].

Moreover, four reviews [19, 34, 35, 36] did not explicitly refer to a predefined methodology (i.e., adherence to a written protocol that was established prior to the conduct of the review with independent verification by a registry such as PROSPERO or another independent body). None of the reviews reported on the sources of funding for the included studies, which may potentially indicate publication bias. Furthermore, several reviews did not employ appropriate methods for the statistical combination of meta‐analysis results. In particular, one review [34] did not investigate the causes of significant heterogeneity, two reviews used multiple data points from individual studies without accounting for the likely dependence between those points [17, 35], whereas three applied a fixed‐effect meta‐analytic model [17, 19, 35], which is unrealistic given that it is unlikely any exercise intervention has a single true effect across samples. There were also five reviews utilizing RoB/quality assessments tools (i.e., either the PEDro tool [35, 37, 38] or TESTEX tools [14, 16]) that do not fully assess bias arising from the selection of reported outcomes, whereas six reviews did not perform analyses to investigate the possible impact of RoB on summary estimates [16, 17, 19, 35, 37, 38]. Taken together, these observations underscore the importance of exercising caution when interpreting certain included reviews and highlight the need for well‐conducted systematic reviews in this field.

### 4.3 Strengths, Limitations, and Future Directions

The strengths of this umbrella review include adherence to PRIOR guidelines and the use of widely recognized benchmarks (e.g., AMSTAR‐2) to assess the scientific rigor of the included systematic reviews. We focused exclusively on the highest level of evidence (i.e., systematic reviews with meta‐analyses) to ensure the robustness of our analyses. With a relatively large sample size of 9639 unique participants with MetS, our additional meta‐analysis resolved overlap and standardized effects to further enhance the accuracy and consistency of our summarized results. This provides the most robust and comprehensive evaluation to date compared to previous reviews and meta‐analyses. Furthermore, we have provided important clinical information and implications on the therapeutic magnitude of various exercise modalities on each individual component of MetS, thus clearly defining the novelty and significance of the work as the largest evaluation specific to MetS.

We acknowledge that this umbrella review represents some limitations. Firstly, some participants in the included studies were diagnosed with MetS and were on medications that were not withdrawn or reduced during the intervention period. This raises the possibility of an interaction between the medication and the exercise training effect. In addition, some of our outcome measures presented relatively high heterogeneity in the total meta‐analysis, and this may reflect the diversity in exercise protocols (e.g., work intensity, duration, and volume) used in the included studies. Although we have included a series of subgroup analyses based on exercise modalities in our present work, it is noted that certain parameters were evaluated based on a relatively small number of studies (e.g., < 10 studies), as indicated in Table 2. Notably, the current evidence is dominated by aerobic exercise studies, with comparatively limited data on resistance training, especially high‐intensity variants. Future research should focus on these underresearched modalities to strengthen the evidence base.

Furthermore, our analysis identified limited reporting of safety and adherence outcomes. Only two of the included reviews [18, 36] reported satisfactory exercise adherence rates among individuals with MetS, whereas others provided no data. Cramer et al. [34] and Poon et al. [18] indicated that some RCTs reported no adverse events, with others omitting safety data entirely. These gaps underscore the need for future studies to systematically document both adherence and adverse events. Additionally, this umbrella review was restricted to exercise‐based interventions, precluding direct comparison with other MetS management strategies such as dietary modifications or pharmacological treatments. Although our focus on modality‐specific efficacy and underlying mechanisms remains a strength, it limits the ability to offer patients a comprehensive overview of all available options. Future research would benefit from advanced approaches, such as network meta‐analysis (NMA), to simultaneously compare the relative efficacy of exercise, diet, pharmacotherapy, and combined interventions in improving MetS components.

### 4.4 Practical Application for Effective MetS Management

From a practical perspective, the findings of this umbrella review have potential implications for clinical practice. The modality‐specific benefits identified in our analysis should empower healthcare professionals to tailor exercise prescriptions to patient preferences, fitness levels, and primary MetS component(s) targeted, therefore enhancing adherence and effectiveness. For instance, aerobic exercise—such as brisk walking or cycling—is effective in offering benefits for all MetS components. Its accessibility and low‐impact nature make it suitable for a broad population, including older adults and those with mobility issues, promoting sustained engagement [7]. Resistance training, emphasizing muscular fitness (including strength, endurance, power, and hypertrophy), is particularly valuable for patients targeting body composition and functional improvement [55]. Combined training, integrating aerobic and resistance elements, provides a balanced, flexible approach for those requiring comprehensive risk factor management [37]. HIIT, with its lower volume and time‐efficient nature, may suit individuals with demanding schedules, offering significant cardiometabolic benefits in shorter sessions [18, 50]. Mind–body exercise—including yoga or tai chi—not only improves metabolic parameters but also may offer distinct psychological benefits by integrating mindfulness and stress‐reduction techniques (e.g., meditation, controlled breathing) [34, 54, 56], which are particularly effective in addressing prevalent comorbidities such as stress and anxiety in individuals with MetS, potentially enhancing adherence compared to traditional exercise. This modality versatility is crucial given the global challenge of suboptimal exercise participation [12] that limits the public health impact of lifestyle interventions. To maximize reach and adherence, clinicians can integrate these interventions into community‐based programs, workplace wellness initiatives, or telehealth platforms [57], leveraging technology to deliver tailored guidance and monitor progress [58, 59]. By aligning exercise prescriptions with individual lifestyles and clinical goals, healthcare providers might facilitate long‐term behavior change, amplifying the effectiveness of MetS management strategies.

## 5 Conclusion

This umbrella review presents a comprehensive and up‐to‐date evidence synthesis supporting the efficacy of exercise as a key nonpharmacological strategy for improving all MetS core components, along with a broad range of clinically relevant cardiometabolic health parameters. Aerobic and mind–body exercises significantly enhanced all MetS components, whereas resistance, HIIT, and combined training improved specific components. Compared to usual care, combined training elicited larger effects on reducing FBG, TG, and blood pressure compared to aerobic or resistance exercise alone. HIIT most effectively reduced WC, whereas mind–body exercise excelled in lowering blood pressure and improving lipid profile. These findings highlight the versatility of exercise, providing healthcare professionals with flexible, patient‐centered treatment options to enhance adherence and outcomes for MetS management. Future systematic reviews in this field should focus on improving methodological quality in reporting to enhance the reliability of the evidence.

## Author Contributions

E.P. and H.L. conceived the idea for the review. E.P., H.L., P.W., and W.S. conducted search, study selection, data extraction, and quality assessment. E.P. drafted the initial manuscript. E.P., H.L., J.L., and A.S. contributed to writing the manuscript. All authors reviewed and approved the final manuscript.

## Funding

The authors have nothing to report.

## Conflicts of Interest

The authors declare no conflicts of interest.

## Supporting information


**Table S1:** Search strategy.
**Table S2:** List of excluded studies with reasons.
**Table S3:** List of included RCTs.
**Table S4:** Summary of meta‐analysis results from included reviews.
**Table S5:** obr_70144‐sup‐0001‐Supplementary_merged.pdf. Summary of risk of bias, methodological quality, certainty of evidence, sensitivity analysis, publication bias, and potential moderators on PA outcomes reported by each included systematic review.

## Data Availability

The datasets analyzed in this review are available from the corresponding author on reasonable request.
