# Supplementary material for: Efficacy of Exercise‐Based Interventions for Metabolic Syndrome: An Umbrella Review With Meta‐Analyses
Source: Obes Rev. 2026 Apr 26;27(11):e70144. doi: 10.1111/obr.70144 (PMC13655955; doi:10.1111/obr.70144)
Supplement: Supplementary file 1 [file OBR-27-e70144-s001.pdf]

Efficacy of Exercise-based Interventions for Metabolic Syndrome: An Umbrella Review with Meta-Analyses

Running title: Exercise for Metabolic Syndrome: Umbrella Review

Authors:

Eric Tsz-Chun POON[1*] PhD (eric.poon@cuhk.edu.hk) ORCID: 0000-0002-0842-1323

Hong-Yat LI[1] MSc (lhy981224@gmail.com) ORCID: 0009-0006-8151-0177

Po-San WONG[1] MSc (sunwong@cuhk.edu.hk) ORCID: 0009-0000-5712-3544

Wesley Man-Kuk SUM[1] BSc (wesleysum@link.cuhk.edu.hk) ORCID: 0009-0007-5409-6094

Jonathan Peter LITTLE[2] PhD (jonathan.little@ubc.ca) ORCID: 0000-0002-9796-2008

Angelo SABAG[3,4] PhD (angelo.sabag@sydney.edu.au) ORCID: 0000-0002-0195-7029

1 Department of Sports Science and Physical Education, The Chinese University of Hong Kong, Shatin, Hong Kong

2 School of Health and Exercise Sciences, University of British Columbia, Kelowna, BC, Canada

3 Sydney School of Health Sciences, The University of Sydney, Sydney, NSW, Australia

4 Charles Perkins Centre, The University of Sydney, Sydney, NSW, Australia

*Correspondence: Eric Tsz-Chun POON, Department of Sports Science and Physical Education, The Chinese University of Hong Kong, Shatin, Hong Kong, Email: eric.poon@cuhk.edu.hk; Tel: +852-3943-7157

Keywords: Physical activity, cardiometabolic health; public health; overview

Acknowledgement: The authors acknowledge and express gratitude to Mr. Eric Ma at the Chinese University of Hong Kong for his data management assistance in this manuscript.

Potential conflicts of interest: All authors have no conflicts of interest to disclose.

Supplementary Table 1 Search strategy

Seven databases (MEDLINE, EMBASE, Cochrane Database, CINAHL, Scopus, SPORTDiscus, and Web of Science) were searched using subject heading, keyword and Medical Subject Headings (MeSH) term searches for ‘metabolic syndrome’, ‘physical activity’, ‘exercise’, ‘systematic review’, and ‘meta-analysis’:

**PubMed**
("systematic review"[Tiab] OR "systematic literature review"[Tiab] OR "cochrane database syst rev"[Tiab] OR "meta-analysis"[Tiab] OR "metaanalysis"[Tiab] OR "meta analysis"[Tiab] OR "meta-analytic review"[Tiab] OR "meta analy"[Tiab] OR "meta-analy"[Tiab] OR "metaanaly*"[Tiab] OR "metareview"[Tiab] OR "meta-review"[Tiab])<br>AND (“metabolic syndrome”[MeSH] OR "metabolic syndrom*"[Tiab] OR "syndrome X"[Tiab] OR "cardiometabolic disease*"[Tiab])<br>AND ("exercise therapy"[MeSH] OR "physical activit*"[Tiab] OR "exercise"[Tiab] OR "lifestyle change"[Tiab] OR "training"[Tiab] OR "workout"[Tiab] OR "sport*"[Tiab])
**Web of Science**
TS=("systematic review" OR "systematic literature review" OR "cochrane database syst rev" OR "meta-analysis" OR "metaanalysis" OR "meta analysis" OR "meta-analytic review" OR "meta analy" OR "meta-analy" OR "metaanaly*" OR "medreview" OR "meta-review")<br>AND TS=("metabolic syndrom*" OR "syndrome X" OR "cardiometabolic disease*")<br>AND TS=("exercise therapy" OR "physical activit*" OR "exercise" OR "lifestyle change" OR "training" OR "workout" OR "sport*")
**Cochrane Library**
((systematic review OR systematic literature review OR cochrane database syst rev OR meta-analysis OR metaanalysis OR meta analysis OR meta-analytic review OR meta analy OR meta-analy OR metaanaly* OR metareview OR meta-review)):ti,ab,kw<br>AND ((metabolic syndrom* OR syndrome X OR cardiometabolic disease*)):ti,ab,kw<br>AND ((exercise therapy OR physical activit* OR exercise OR lifestyle change OR training OR workout OR sport*)):ti,ab,kw
**SPORTDiscus**
((systematic review OR systematic literature review OR cochrane database syst rev OR meta-analysis OR metaanalysis OR meta analysis OR meta-analytic review OR meta analy OR meta-analy OR metaanaly* OR metareview OR meta-review))

AND ((metabolic syndrom* OR syndrome X OR cardiometabolic disease*))<br>AND ((exercise therapy OR physical activit* OR exercise OR lifestyle change OR training OR workout OR sport*))
**CINAHL**
((systematic review OR systematic literature review OR cochrane database syst rev OR meta-analysis OR metaanalysis OR meta analysis OR meta-analytic review OR meta analy OR meta-analy OR metaanaly* OR metareview OR meta-review))<br>AND ((metabolic syndrom* OR syndrome X OR cardiometabolic disease*))<br>AND ((exercise therapy OR physical activit* OR exercise OR lifestyle change OR training OR workout OR sport*))
**Scopus**
(TITLE-ABS-KEY ("systematic review" OR "systematic literature review" OR "cochrane database syst rev" OR "meta-analysis" OR "metaanalysis" OR "meta analysis" OR "meta-analytic review" OR "meta analy" OR "meta-analy" OR "metaanaly*" OR "metareview" OR "meta-review")<br>AND TITLE-ABS-KEY ("metabolic syndrom*" OR "syndrome X" OR "cardiometabolic disease*")<br>AND TITLE-ABS-KEY ("exercise therapy" OR "physical activit*" OR "exercise" OR "lifestyle change" OR "training" OR "workout" OR "sport*"))
**EMBASE**
(("systematic review" OR "systematic literature review" OR "cochrane database syst rev" OR "meta-analysis" OR "metaanalysis" OR "meta analysis" OR "meta-analytic review" OR "meta analy" OR "meta-analy" OR "metaanaly*" OR "metareview" OR "meta-review")<br>AND ("metabolic syndrom*" OR "syndrome X" OR "cardiometabolic disease*")<br>AND ("exercise therapy" OR "physical activit*" OR "exercise" OR "lifestyle change" OR "training" OR "workout" OR "sport*")).ti,ab.

Supplementary Table 2 List of excluded studies with reasons

Reference	Journal	Title	Reason
Afshar Naseri 2024	Asian Journal of Sports Medicine	Isometric Exercise for Blood Pressure and Endothelial Function in Metabolic Syndrome: A Review	No meta-analysis
Aguilera EguÃa et al. 2015	Nutricion Hospitalaria	EJERCICIO INTERVALADO DE ALTA INTENSIDAD COMO TERAPIA PARA DISMINUIR LOS FACTORES DE RIESGO CARDIOVASCULAR EN PERSONAS CON SÃçNDROME METABÃ“LICO; REVISIÃ“N SISTEMÃÅTICA CON METAANÃÅLISIS	No English full text
Amagasa et al. 2018	Int J Behav Nutr Phys Act	Is objectively measured light-intensity physical activity associated with health outcomes after adjustment for moderate-to-vigorous physical activity in adults? A systematic review	Irrelevant to exercise intervention and population diagnosed with MetS
Amin et al. 2023	Sports (Basel)	Effect of Physical Activity on Metabolic Syndrome Markers in Adults with Type 2 Diabetes: A Systematic Review and Meta-Analysis	Irrelevant to exercise intervention and population diagnosed with MetS
Asiah et al. 2023	Complementary Therapies in Medicine	Effect of yoga on anthropometry, quality of life, and lipid profile in patients with obesity and central obesity: A systematic review and meta-analysis	Irrelevant to exercise intervention and population diagnosed with MetS
Bassi et al. 2014	Am J Med	Lifestyle modification for metabolic syndrome: a systematic review	No meta-analysis
Batalau et al. 2013	Atencion Primaria	Association between physical activity, fitness and cardiovascular risk factors in children. Systematic review	No meta-analysis
Bouzalmate Hajjaj et al. 2022	Sci Rep	A systematic review and meta-analysis of weight loss in control group participants of lifestyle randomized trials	No isolated exercise intervention subgroup analysis
Cabrera et al. 2021	Nutrients	Effectiveness of Workplace-Based Diet and Lifestyle Interventions on Risk Factors in Workers with Metabolic Syndrome: A Systematic Review, Meta-Analysis and Meta-Regression	No MetS subgroup analysis
Carroll et al. 2004	Sports Med	What is the relationship between exercise and metabolic abnormalities? A review of the metabolic syndrome	No MetS subgroup analysis
Chan et al. 2024	European Journal of Preventive Cardiology	Comparative effectiveness of non-pharmacological interventions on metabolic syndrome and its components: a systematic review and network meta-analysis	No full text found
Chau et al. 2021	Complement Ther Clin Pract	Effects of Tai Chi on health outcomes among community-dwelling adults with or at risk of metabolic syndrome: A systematic review	No MetS subgroup analysis
Chen et al. 2020	BMJ Open	Effect of electronic health interventions on metabolic syndrome: a systematic review and meta-analysis	No isolated exercise intervention subgroup analysis
Chen et al. 2022	Zhejiang Da Xue Xue Bao Yi Xue Ban	Summary of the best evidence of diet and physical activity management in patients with metabolic syndrome	No meta-analysis
Cicero Teixeira et al. 2023	Retos: Nuevas Perspectivas de EducaciÃ³n FÃsica, Deporte y RecreaciÃ³n	Quais protocolos de TIAI sÃ£o mais utilizados em pessoas com sÃndrome metabÃ³lica? uma revisÃ£o sistemÃ¡tica	No English full text
da Silva Menezes et al. 2022	European Journal of Molecular and Clinical Medicine	THE EFFECTS OF COMBINED PHYSICAL TRAINING ON INSULIN RESISTANCE AND MUSCLE FUNCTION IN METABOLIC SYNDROME: A SYSTEMATIC REVIEW	No full text found
Das et al. 2021	Sains Malaysiana	The Effect of Type, Duration and Intensity of Exercise on Inflammatory Markers CRP, IL-6 and IL-18 in Metabolic Syndrome Patients: A Systematic Review	No meta-analysis
Deng et al. 2024	Int J Obes (Lond)	Combined lifestyle factors and metabolic syndrome risk: a systematic review and meta-analysis	Irrelevant to exercise intervention and population diagnosed with MetS
Doewes et al. 2023	Current Problems in Cardiology	An Updated Systematic Review on the Effects of Aerobic Exercise on Human Blood Lipid Profile	No meta-analysis

Eckstein et al. 2022	J Clin Med	Physiological Responses to Combat Sports in Metabolic Diseases: A Systematic Review	No meta-analysis
EguÃa et al. 2015	Nutricion Hospitalaria	HIGH-INTENSITY INTERVAL EXERCISE THERAPY TO REDUCE CARDIOVASCULAR RISK FACTORS IN PEOPLE WITH THE METABOLIC SYNDROME; SYSTEMATIC REVIEW WITH META-ANALYSIS	No English full text
El Haddad et al. 2023	Patient Prefer Adherence	Determinants of Non-Adherence to Exercise or Physical Activity in People with Metabolic Syndrome: A Mixed Methods Review	No meta-analysis
Ferret et al. 2021	Journal of Physical Education & Sport	Interventions based on practice of resistance exercises: a systematic review	No meta-analysis
Franssen et al. 2020	International Journal of Behavioral Nutrition & Physical Activity	Can consumer wearable activity tracker-based interventions improve physical activity and cardiometabolic health in patients with chronic diseases? A systematic review and meta-analysis of randomised controlled trials	Irrelevant to exercise intervention and population diagnosed with MetS
GÃ³mez-ÃÅlvarez et al. 2019	Cultura, Ciencia y Deporte	Efectos del entrenamiento de fuerza muscular en mujeres postmenopÃ¡usicas con sÃndrome metabÃ³lico. RevisiÃ³n sistemÃ¡tica	No English full text
GÃ³mez-Alvarez et al. 2019	Cultura Ciencia Y Deporte	Effects of muscle strength training in postmenopausal women with metabolic syndrome. Systematic review	No English full text
Gu et al. 2019	Diabetes-Metabolism Research and Reviews	Aerobic exercise training may improve nerve function in type 2 diabetes and pre-diabetes: A systematic review	No meta-analysis
Guinhouya et al. 2011	Int J Pediatr Obes	Evidence of the influence of physical activity on the metabolic syndrome and/or on insulin resistance in pediatric populations: a systematic review	No meta-analysis
Guretzki et al. 2024	Sensors (Basel)	Effects of Whole-Body Electromyostimulation on Metabolic Syndrome in Adults at Moderate-to-High Cardiometabolic Risk-A Systematic Review and Meta-Analysis	No MetS subgroup analysis
Gurusamy et al. 2018	Asian J Psychiatr	Exercise, diet and educational interventions for metabolic syndrome in persons with schizophrenia: A systematic review	No meta-analysis
GuzmÃ¡n et al. 2019	Biomedica	Effectiveness of interventions for the reversal of a metabolic syndrome diagnosis: An update of a meta-analysis of mixed treatment comparison studies	No English full text
Han et al. 2023	Front Physiol	Meta-analysis of the intervention effects of taekwondo on metabolic syndrome indicators	Irrelevant to exercise intervention and population diagnosed with MetS
Hartley et al. 2014	Cochrane Database of Systematic Reviews	Yoga for the primary prevention of cardiovascular disease	No MetS subgroup analysis
Hassan et al. 2023	J Coll Physicians Surg Pak	Effects of Physical Activity on Thyroid Stimulating Hormone Levels in Obese Metabolic Syndrome Patients: A Meta-Analysis	No MetS subgroup analysis
Hwang et al. 2011	Journal of Cardiopulmonary Rehabilitation and Prevention	Effect of Aerobic Interval Training on Exercise Capacity and Metabolic Risk Factors in People With Cardiometabolic Disorders A META-ANALYSIS	No MetS subgroup analysis
ìù´ê±´ì•„ et al. 2015	Journal of Korean Academy of Nursing	ëŒ€ì‚¬ì¦ùí›„êµ°ì—ê ëŒ€í•œ ì‹ùìù´ì™€ ìš´ëè™ íš¨ê³¼ì—ê ëŒ€í•œ ë©”íƒ€ë¶„ì„ù	No English full text
Innes et al. 2005	Journal of the American Board of Family Practice	Risk indices associated with the insulin resistance syndrome, cardiovascular disease, and possible protection with yoga: A systematic review	No meta-analysis
Janssen et al. 2010	Int J Behav Nutr Phys Act	Systematic review of the health benefits of physical activity and fitness in school-aged children and youth	No meta-analysis
Johnson et al. 2018	Evidence-Based Complementary and Alternative	Mind-Body Therapies for African-American Women at Risk for Cardiometabolic Disease: A Systematic Review	No meta-analysis

	Medicine		
Joseph et al. 2019	Diabetes Metab Syndr Obes	The Impact Of Structured Exercise Programs On Metabolic Syndrome And Its Components: A Systematic Review	No meta-analysis
Junges et al. 2015	Scientia Medica	Effect of Pilates exercises in risk factors for cardiometabolic diseases: a systematic review	No full text found
Junior et al. 2013	Revista Brasileira de PrescriÃ§Ã£o e Fisiologia do ExercÃcio	EXERCÃçCIO FÃçSICO RESISTIDO E SÃçNDROME METABÃ“LICA: UMA REVISÃƒO SISTEMÃÅTICA	No English full text
Kaur 2014	Cardiology Research & Practice	A Comprehensive Review on Metabolic Syndrome	Article retracted
Keating et al. 2017	Obesity Reviews	A systematic review and meta-analysis of interval training versus moderate-intensity continuous training on body adiposity	No MetS subgroup analysis
Kemmler et al. 2010	Deutsche Zeitschrift Fur Sportmedizin	Effects of Whole-Body-Electromyostimulation on Body Composition and Cardiac Risk Factors in Elderly Men with the Metabolic Syndrome. The TEST-II Study	No meta-analysis
KesztyÃ¼s et al. 2018	Deutsches Arzteblatt International	Therapeutic Treatment for Abdominal Obesity in Adults A Meta-Analysis and Systematic Review of Randomized Controlled Trials	No isolated exercise intervention subgroup analysis
Khalafi et al. 2020	Scand J Med Sci Sports	The impact of high-intensity interval training on inflammatory markers in metabolic disorders: A meta-analysis	No MetS subgroup analysis
Khalafi et al. 2022	Iranian Journal of Endocrinology and Metabolism	The Effect of Exercise Training on Inflammatory Markers in Patient with Metabolic Syndrome: A Systematic Review and Meta-analysis	No English full text
Kirk et al. 2019	Am J Health Promot	Wearable Technology and Physical Activity Behavior Change in Adults With Chronic Cardiometabolic Disease: A Systematic Review and Meta-Analysis	No MetS subgroup analysis
Konerman et al. 2019	Journal of the American College of Cardiology	STRUCTURED EXERCISE PROGRAMS FOR TREATMENT OF METABOLIC SYNDROME: A SYSTEMATIC REVIEW	No meta-analysis
Kuo et al. 2014	Worldviews Evid Based Nurs	Effectiveness of empowerment-based self-management interventions on patients with chronic metabolic diseases: a systematic review and meta-analysis	Irrelevant to exercise intervention and population diagnosed with MetS
Lee et al. 2015	Journal of Korean Academy of Nursing	Effects of Dietary and Physical Activity Interventions on Metabolic Syndrome: A Meta-analysis	No English full text
Lei et al. 2022	Exercise Science	Does Taekwondo Poomsae Training Impact on Body Composition, Physical Fitness, and Blood Composition in Children and Adolescents? A Systematic Review	No meta-analysis
Levinger et al. 2015	Clin Med Insights Cardiol	What Doesn't Kill You Makes You Fitter: A Systematic Review of High-Intensity Interval Exercise for Patients with Cardiovascular and Metabolic Diseases	No meta-analysis
Li et al. 2021	Chinese Journal of School Health	Meta-analysis of the effects of high-intensity interval training on metabolic syndrome in overweight college students	No full text found
Liang et al. 2021	Rev Cardiovasc Med	Effects of aerobic, resistance, and combined exercise on metabolic syndrome parameters and cardiovascular risk factors: a systematic review and network meta-analysis	No MetS subgroup analysis
Lin et al. 2014	Worldviews on Evidence-Based Nursing	Systematic Review of Impact of Lifestyle-Modification Programs on Metabolic Risks and Patient-Reported Outcomes in Adults With Metabolic Syndrome	No meta-analysis
Lin et al. 2015	Journal of the American Heart Association	Effects of Exercise Training on Cardiorespiratory Fitness and Biomarkers of Cardiometabolic Health: A Systematic Review and Meta-Analysis of Randomized Controlled Trials	Irrelevant to exercise intervention and population diagnosed with MetS
Malakou et al. 2018	Nutrients	The combined effect of promoting the mediterranean diet and physical activity on metabolic risk factors in adults: A systematic review and meta-analysis of randomised controlled trials	No MetS subgroup analysis

Marcos-Delgado et al. 2021	Int J Environ Res Public Health	The Effect of Lifestyle Intervention on Health-Related Quality of Life in Adults with Metabolic Syndrome: A Meta-Analysis	No isolated exercise intervention subgroup analysis
Marshall et al. 2013	American Journal of Preventive Medicine	Weight-Related Child Behavioral Interventions in Brazil A Systematic Review	No meta-analysis
Martemucci et al. 2024	Metabolites	Comprehensive Strategies for Metabolic Syndrome: How Nutrition, Dietary Polyphenols, Physical Activity, and Lifestyle Modifications Address Diabesity, Cardiovascular Diseases, and Neurodegenerative Conditions	No meta-analysis
Mohsenzadeh-Ledari et al. 2019	Int J Prev Med	Appropriate Interventions for Pregnant Women with Indicators of Metabolic Syndrome on Pregnancy Outcomes: A Systematic Review	No meta-analysis
Nafisah et al. 2023	Indonesian Journal of Human Nutrition	Impact of Mobile Applications on Improving Healthy Lifestyle of Patients with Metabolic Syndrome: A Systematic Review	No meta-analysis
Nailufar et al. 2022	Current Research in Nutrition and Food Science	The Effectiveness of Implementation of Healthy Living Community Movement in Overcoming Metabolic Syndrome Among Female Workers in East Kalimantan, Indonesia	No meta-analysis
Nambiar et al. 2014	Journal of Science and Medicine in Sport	A systematic review to assess the impact of physical activity intervention on people with metabolic syndrome	No full text found
Nele Pattyn et al. 2012	European Journal of Preventive Cardiology	The effect of exercise on the risk factors of the metabolic syndrome in metabolic syndrome patients: A meta-analysis of controlled trials	No full text found
Nizamani et al. 2022	Diabetes and Metabolic Syndrome: Clinical Research and Reviews	Couples-based behaviour change interventions to reduce metabolic syndrome risk. A systematic review	Irrelevant to exercise intervention and population diagnosed with MetS
Ortiz-GuzmÃ¡n et al. 2023	Journal of Clinical Medicine	Short-Term Heart Rate Variability in Metabolic Syndrome: A Systematic Review and Meta-Analysis	Irrelevant to exercise intervention and population diagnosed with MetS
PÃ©rez et al. 2018	Nutrients	New Insights about How to Make an Intervention in Children and Adolescents with Metabolic Syndrome: Diet, Exercise vs. Changes in Body Composition. A Systematic Review of RCT	No meta-analysis
PÃ©rez et al. 2019	International Journal of Environmental Research and Public Health	Practical Guidance for Interventions in Adults with Metabolic Syndrome: Diet and Exercise vs. Changes in Body Composition	No meta-analysis
Papanastasiou 2012	Therapeutic Advances in Endocrinology and Metabolism	Interventions for the metabolic syndrome in schizophrenia: A review	No meta-analysis
Parameshwar et al. 2021	Curr Diabetes Rev	Lifestyle Modification with Physical Activity Promotion on Leptin Resistance and Quality of Life in Metabolic Syndrome - A Systematic Review with Meta-Analysis	No full text found
Park et al. 2024	J Nurs Scholarsh	Comprehensive lifestyle modification interventions for metabolic syndrome: A systematic review and meta-analysis	No isolated exercise intervention subgroup analysis
Pashkevych et al. 2024	Phytotherapy Journal	WAYS TO IMPROVE THE QUALITY OF LIFE OF PATIENTS WITH METABOLIC SYNDROME: A SYSTEMATIC REVIEW	No meta-analysis
Patterson et al. 2020	JBI Evid Synth	Experiences of providers in delivering nutrition-focused lifestyle interventions for adults with obesity or metabolic syndrome in primary healthcare settings: a qualitative systematic review protocol	No meta-analysis
Peiris et al. 2021	Rev Endocr Metab Disord	Education-based, lifestyle intervention programs with unsupervised exercise improve outcomes in adults with metabolic syndrome. A systematic review and meta-analysis	No isolated exercise intervention subgroup analysis
Peiris et al. 2023	Patient Prefer Adherence	Behavior Change Techniques Improve Adherence to Physical Activity Recommendations for Adults with Metabolic Syndrome: A Systematic Review	No meta-analysis
Rahimi et al. 2022	Biol Res Nurs	Effects of Lifestyle Intervention on Inflammatory Markers and Waist Circumference in Overweight/Obese Adults With Metabolic Syndrome: A Systematic Review and Meta-Analysis of Randomized Controlled Trials	No isolated exercise intervention subgroup analysis

Rai et al. 2023	Journal of Diabetes and Metabolic Disorders	Impact of exercise training duration on obesity and cardiometabolic biomarkers: a systematic review	No meta-analysis
Reig Garcia-Galbis et al. 2017	Annals of Nutrition and Metabolism	Design of clinical trials in adults with metabolic syndrome. Systematic review	No full text found
Renninger et al. 2020	Pediatr Obes	Associations between accelerometry measured physical activity and sedentary time and the metabolic syndrome: A meta-analysis of more than 6000 children and adolescents	Irrelevant to exercise intervention and population diagnosed with MetS
Rodrigues et al. 2016	Preventive Medicine	A systematic review and meta-analysis of primary prevention programmes to improve cardio-metabolic risk in non-urban communities	Irrelevant to exercise intervention and population diagnosed with MetS
SÃ¡-Caputo et al. 2014	Rehabilitation Research & Practice	Whole Body Vibration Exercises and the Improvement of the Flexibility in Patient with Metabolic Syndrome	No meta-analysis
Sadeghi et al. 2016	Journal of Research in Medical Sciences	Effect of cardiac rehabilitation on metabolic syndrome and its components: A systematic review and meta-analysis	No isolated exercise intervention subgroup analysis
Salinas-Escudero et al. 2023	Salud Uninorte	Interventions for the Control and Prevention of Metabolic Syndrome. A Systematic Review of a Decade	No meta-analysis
Sequi-Dominguez et al. 2020	J Med Internet Res	Effectiveness of Mobile Health Interventions Promoting Physical Activity and Lifestyle Interventions to Reduce Cardiovascular Risk Among Individuals With Metabolic Syndrome: Systematic Review and Meta-Analysis	No isolated exercise intervention subgroup analysis
Sousa et al. 2017	Sports Medicine	The Antioxidant Effect of Exercise: A Systematic Review and Meta-Analysis	No MetS subgroup analysis
Stone 2008	American Journal of Cardiology	Nonpharmacologic management of mixed dyslipidemia associated with diabetes mellitus and the metabolic syndrome: a review of the evidence	No meta-analysis
Strasser et al. 2010	Sports Med	Resistance training in the treatment of the metabolic syndrome: a systematic review and meta-analysis of the effect of resistance training on metabolic clustering in patients with abnormal glucose metabolism	Irrelevant to exercise intervention and population diagnosed with MetS
Tegegne et al. 2024	Health Policy and Technology	Digital lifestyle interventions for cardiovascular risk reduction: A systematic review and network meta-analysis	Irrelevant to exercise intervention and population diagnosed with MetS
Teixeira et al. 2023	Retos-Nuevas Tendencias En Educacion Fisica Deporte Y Recreacion	Which HIIT protocols are most used in people with metabolic syndrome? a systematic review	No meta-analysis
Thomas et al. 2023	Journal of Medical Internet Research	Accelerometer-Measured Physical Activity Data Sets (Global Physical Activity Data Set Catalogue) That Include Markers of Cardiometabolic Health: Systematic Scoping Review	No meta-analysis
Tsirogianni et al. 2024	Clinical Nutrition Espen	The usefulness of randomized trials of lifestyle interventions for overweight, obesity, or metabolic syndrome: A systematic review	No meta-analysis
van Namen et al. 2019	Metabolism	Supervised lifestyle intervention for people with metabolic syndrome improves outcomes and reduces individual risk factors of metabolic syndrome: A systematic review and meta-analysis	No isolated exercise intervention subgroup analysis
VelÃ¡squez et al. 2017	Endocrinologia Diabetes Y Nutricion	Systematization of clinical trials related to treatment of metabolic syndrome, 1980-2015	No English full text
Verheggen et al. 2016	Obesity Reviews	A systematic review and meta-analysis on the effects of exercise training versus hypocaloric diet: distinct effects on body weight and visceral adipose tissue	No MetS subgroup analysis
Villamil-Parra et al. 2024	Exp Gerontol	Effects of physical exercise on Irisin and BDNF concentrations, and their relationship with cardiometabolic and mental health of individuals with Metabolic Syndrome: A Systematic Review	No meta-analysis

Weston et al. 2014	Br J Sports Med	High-intensity interval training in patients with lifestyle-induced cardiometabolic disease: a systematic review and meta-analysis	No MetS subgroup analysis
Yamaoka et al. 2012	Bmc Medicine	Effects of lifestyle modification on metabolic syndrome: a systematic review and meta-analysis	No isolated exercise intervention subgroup analysis
Yu et al. 2023	Iran J Public Health	Effect of Baduanjin Exercise on Metabolic Syndrome Risk: A Systematic Review and Meta-Analysis of Randomized Controlled Trials	Irrelevant to exercise intervention and population diagnosed with MetS
Yuen Li 2016		Effects of Tai Chi Exercise for Community-dwelling Chinese Adults with Metabolic Syndrome	No peer review
Zhang et al. 2017	Metabolism	Leisure-time physical activity and incident metabolic syndrome: a systematic review and dose-response meta-analysis of cohort studies	Irrelevant to exercise intervention and population diagnosed with MetS
Zimbron et al. 2016	Eur Neuropsychopharmacol	A systematic review and meta-analysis of randomised controlled trials of treatments for clozapine-induced obesity and metabolic syndrome	No isolated exercise intervention subgroup analysis
Zou et al. 2019	Int J Environ Res Public Health	Wuqinxi Qigong as an Alternative Exercise for Improving Risk Factors Associated with Metabolic Syndrome: A Meta-Analysis of Randomized Controlled Trials	Irrelevant to exercise intervention and population diagnosed with MetS

Supplementary Table 3 List of included RCT

Reference	Year	Article	Analyzed Subject (n)
Alvarez	2018	High-intensity interval training as a tool for counteracting dyslipidemia in women	49
Alvarez	2016	Low-Volume high-intensity interval training as a therapy for type 2 diabetes.	23
Anderssen	2007	Combined diet and exercise intervention reverses the metabolic syndrome in middle-aged males: results from the Oslo Diet and Exercise Study	137
Anderssen	1995	Oslo diet and exercise study - A one-year randomised intervention trial - Effect on hemostatic variables and other coronary risk-factors.	92
Arija	2017	Effectiveness of a physical activity program on cardiovascular disease risk in adult primary health-care users: the "Pas-a-Pas" community intervention trial.	324
Aristizabal	2021	Effects of Low-Volume, High-Intensity Interval Training Compared with Continuous Training on Regional and Global Body Composition in Adults with Metabolic Syndrome: a post hoc Analysis of a Randomized Clinical Trial.	60
Balducci	2010a	Anti-inflammatory effect of exercise training in subjects with type 2 diabetes and the metabolic syndrome is dependent on exercise modalities and independent of weight loss	82
Balducci	2010b	Effect of an Intensive Exercise Intervention Strategy on Modifiable Cardiovascular Risk Factors in Subjects With Type 2 Diabetes Mellitus	563
Cao	2019	Exercise training at maximal fat oxidation intensity for overweight or obese older women: a randomized study.	28
Cardoso	2016	Resistance exercise does not change components and markers of metabolic syndrome in pre- and postmenopausal period	43
Castaneda	2002	A randomized controlled trial of resistance exercise training to improve glycemic control in older adults with type 2 diabetes.	60
Chan	2018	Tai chi exercise is more effective than brisk walking in reducing cardiovascular disease risk factors among adults with hypertension: a randomised controlled trial.	246
Choi	2012	Effects of exercise on sRAGE levels and cardiometabolic risk factors in patients with type 2 diabetes: a randomized controlled trial.	75
Cohen	2008	Restorative yoga in adults with metabolic syndrome: A randomized, controlled pilot trial	24
Conners	2019	Impact of underwater treadmill training on glycemic control, blood lipids, and health-related fitness in adults with type 2 diabetes.	26
Dai	2019	Two-year-supervised resistance training prevented diabetes incidence in people with prediabetes: a randomised control trial. *Diabetes Metab Res Rev*	137
Damichi	2014	Influence of aerobic training and detraining on serum BDNF, insulin resistance, and metabolic risk factors in middle-aged men diagnosed with metabolic syndrome	21
de Matos	2021	Two Weekly Sessions of High-Intensity Interval Training Improve Metabolic Syndrome and Hypertriglyceridemic Waist Phenotype in Older Adults: a Randomized Controlled Trial.	104
DeVallance	2016	The effects of resistance exercise training on arterial stiffness in metabolic syndrome	57
Doğan Dede	2015	Influence of exercise on leptin, adiponectin and quality of life in type 2 diabetics.	60
Donley	2014	Aerobic exercise training reduces arterial stiffness in metabolic syndrome	43
Dumortier	2003	Low intensity endurance exercise targeted for lipid oxidation improves body composition and insulin sensitivity in patients with the metabolic syndrome.	39
Dunstan	2002	High-intensity resistance training improves glycemic control in older patients with type 2 diabetes.	29
Earnest	2014	Aerobic and strength training in concomitant metabolic syndrome and type 2 diabetes.	262
Fang	2019	Effectiveness of a physical activity program on weight, physical fitness, occupational stress, job satisfaction and quality of life of overweight employees in high-tech industries: a randomized controlled study.	75
Farag	2019	Effects of vitamin C supplementation with and without endurance physical activity on components of metabolic syndrome: a randomized, double-blind, placebo-controlled clinical trial.	120
Farinatti	2016	Long term home-based exercise is effective to reduce blood pressure in low income Brazilian hypertensive patients: a controlled trial.	43
Gallo-Villegas	2022	Efficacy of high-intensity interval- or continuous aerobic-training on insulin resistance and muscle function in adults with metabolic syndrome: a clinical trial.	60
Gao	2024	Effect of Exercise Training on Some Anti-Inflammatory Adipokines, High Sensitivity C-Reactive Protein, and Clinical Outcomes in Sedentary Adults With Metabolic Syndrome.	33
Gomes	2008	Enhanced concentrations of relevant markers of nitric oxide formation after exercise training in patients with metabolic syndrome	48

Gordon	2008	Effect of exercise therapy on lipid profile and oxidative stress indicators in patients with type 2 diabetes.	231
Gram	2010	Effects of Nordic walking and exercise in type 2 diabetes mellitus: a randomized controlled trial.	68
Heiston	2020	Exercise improves adiposopathy, insulin sensitivity and metabolic syndrome severity independent of intensity.	28
Huei (Phing)	2017	Effectiveness of physical activity intervention among government employees with metabolic syndrome.	155
Irving	2008	Effect of exercise training intensity on abdominal visceral fat and body composition	27
Irving	2009	Effects of Exercise Training Intensity on Nocturnal Growth Hormone Secretion in Obese Adults with the Metabolic Syndrome	34
Jiang	2020	Aerobic exercise training at maximal fat oxidation intensity improves body composition, glycemic control, and physical capacity in older people with type 2 diabetes	49
Jin	2021	Study on the effect of health-care qigong baduanjin exercises on metabolic syndrome	90
Jo	2020	Effects of High-Intensity Interval Training Versus Moderate-Intensity Continuous Training on Epicardial Fat Thickness and Endothelial Function in Hypertensive Metabolic Syndrome.	34
Kadoglou	2010	Exercise ameliorates serum MMP-9 and TIMP-2 levels in patients with type 2 diabetes.	47
Kanaya	2014	Restorative yoga and metabolic risk factors: the Practicing Restorative Yoga vs. Stretching for the Metabolic Syndrome (PRYSMS) randomized trial	180
Kang	2016	Effects of aerobic exercise on the resting heart rate, physical fitness, and arterial stiffness of female patients with metabolic syndrome	23
Kazemi	2023	Effect of resistance training and high-intensity interval training on metabolic parameters and serum level of Sirtuin1 in postmenopausal women with metabolic syndrome: a randomized controlled trial.	45
Ke	2020	Effect of Baduanjin and lifestyle on patients with metabolic syndrome	41
Kim	2011	Effects of a cardiovascular risk reduction intervention with psychobehavioral strategies for Korean adults with type 2 diabetes and metabolic syndrome	43
Kim	2012	Effects of aerobic exercise training on serum sex hormone binding globulin, body fat index, and metabolic syndrome factors in obese postmenopausal women.	30
Kim	2013	Effects of yoga on sexual function in women with metabolic syndrome: A randomized controlled trial	37
Kukkonen-Harjula	2005	Effects of a weight maintenance program with or without exercise on the metabolic syndrome: a randomized trial in obese men.	90
Laaksonen	2000	Aerobic exercise and the lipid profile in type 1 diabetic men: a randomized controlled trial.	42
Labrunée	2012	Effects of a home-based rehabilitation program in obese type 2 diabetics.	23
Lambers	2008	Influence of combined exercise training on indices of obesity, diabetes and cardiovascular risk in type 2 diabetes patients.	46
Landaeta-Díaz	2013	Mediterranean diet, moderate-to-high intensity training, and health-related quality of life in adults with metabolic syndrome	40
Lavrencic	2000	Physical training improves flow-mediated dilation in patients with the polymetabolic syndrome.	29
Lehmann	1995	Loss of abdominal fat and improvement of the cardiovascular risk profile by regular moderate exercise training in patients with NIDDM.	29
Leung	2019	Tai Chi in Chinese adults with metabolic syndrome: A pilot randomized controlled trial	35
Liao	2013	Effects of the traditional Chinese medicine fitness method of Baduanjin on patients with metabolic syndrome	140
Ligtenberg	1997	Effects of physical training on metabolic control in elderly type 2 diabetes mellitus patients.	51
Lima	2012	Aerobic training does not alter blood pressure in menopausal women with metabolic syndrome	44
Liu	2012	Correlation research on effect of wuqinxi exercise on cognitive function in old people with metabolic syndrome	40
Madden	2013	Aerobic training-induced improvements in arterial stiffness are not sustained in older adults with multiple cardiovascular risk factors.	52
Mager	2008	Expression of ghrelin gene in peripheral blood mononuclear cells and plasma ghrelin concentrations in patients with metabolic syndrome	75
Manchanda	2013	Reversal of early atherosclerosis in metabolic syndrome by yoga – A randomized controlled trial	100
Maresca	2013	Exercise training improves erectile dysfunction (ED) in patients with metabolic syndrome on phosphodiesterase-5 (PDE-5) inhibitors	20

Matoyama	1995	The effects of long-term low intensity aerobic training and detraining on serum lipid and lipoprotein concentrations in elderly men and women.	30
Mendoza-Núñez	2018	Hypoglycemic and antioxidant effect of Tai chi exercise training in older adults with metabolic syndrome	85
Morales-Palomo	2019	Effectiveness of aerobic exercise programs for health promotion in metabolic syndrome	121
Morales-Palomo	2017	Effects of repeated yearly exposure to exercise-training on blood pressure and metabolic syndrome evolution	49
Mora-Rodriguez	2017	Aerobic interval training reduces vascular resistances during submaximal exercise in obese metabolic syndrome individuals	34
Mora-Rodriguez	2018a	Effects of aerobic interval training on arterial stiffness and microvascular function in patients with metabolic syndrome	46
Mora-Rodriguez	2019	Insulin sensitivity improvement with exercise training is mediated by body weight loss in subjects with metabolic syndrome	96
Mora-Rodriguez	2018b	Weight loss but not gains in cardiorespiratory fitness after exercise-training predicts improved health risk factors in metabolic syndrome	160
Moreno-Cabañas	2021	Substitution of parts of aerobic training by resistance training lowers fasting hyperglycemia in individuals with metabolic syndrome.	87
Oh	2010	Effects of a 6-month lifestyle modification intervention on the cardiometabolic risk factors and health-related qualities of life in women with metabolic syndrome	52
Okura	2007	Effects of aerobic exercise on metabolic syndrome improvement in response to weight reduction	59
Paolillo	2017	Effects of phototherapy plus physical training on metabolic profile and quality of life in postmenopausal women.	30
Ramirez-Jimenez	2020	Effects of Exercise Training during Christmas on Body Weight and Cardiometabolic Health in Overweight Individuals.	38
Ramos	2016a	12 min/week of high-intensity interval training reduces aortic reservoir pressure in individuals with metabolic syndrome.	50
Ramos	2021	Exercise Training Intensity and the Fitness-Fatness Index in Adults with Metabolic Syndrome: a Randomized Trial.	77
Ramos	2017a	High-intensity interval training and cardiac autonomic control in individuals with metabolic syndrome: a randomised trial.	56
Ramos	2017b	Low-volume high-intensity interval training is sufficient to ameliorate the severity of metabolic syndrome	65
Ramos	2016b	The effect of different volumes of high-intensity interval training on proinsulin in participants with the metabolic syndrome: a randomised trial.	66
Raz	1994	Moderate exercise improves glucose metabolism in uncontrolled elderly patients with non-insulin-dependent diabetes mellitus.	38
Reljic	2021a	Effects of very low volume high intensity versus moderate intensity interval training in obese metabolic syndrome patients: a randomized controlled study	87
Reljic	2022(a)	HIIT the inflammation”: comparative effects of low-volume interval training and resistance exercises on inflammatory indices in obese metabolic syndrome patients undergoing caloric restriction.	104
Reljic	2023	Maximum heart rate- and lactate threshold-based low-volume high-intensity interval training prescriptions provide similar health benefits in metabolic syndrome patients.	58
Reljic	2021b	Very low-volume interval training improves nonalcoholic fatty liver disease fibrosis score and cardiometabolic health in adults with obesity and metabolic syndrome	65
Reseland	2001	Effect of long-term changes in diet and exercise on plasma leptin concentrations	186
Rönnemaa	1988	Effects of long-term physical exercise on serum lipids, lipoproteins and lipid metabolizing enzymes in type 2 (non-insulin-dependent) diabetic patients.	25
Rosety-Rodriguez	2013	A short-term training program reduced acute phase proteins in premenopausal women with metabolic syndrome	135
Saremi	2011	Effects of twelve-week strength training on serum chemerin, TNF-α and CRP level in subjects with the metabolic syndrome.	32
Sari-Sarraf	2015	A combined continuous and interval aerobic training improves metabolic syndrome risk factors in men	30
Shakil-Ur-Rehman	2017	Effects of supervised structured aerobic exercise training program on high and low density lipoprotein in patients with type II diabetes mellitus.	195
Shi	2013	Intervention of metabolic syndrome by yijinjing exercise combined with acupuncture in community.	180
Sigal	2007	Effects of aerobic training, resistance training, or both on glycemic control in type 2 diabetes: a randomized trial	251
Silva	2022	Combining different concurrent training methods in older adults with metabolic syndrome.	39

Silva	2020	The Effects of Concurrent Training Combining Both Resistance Exercise and High-Intensity Interval Training or Moderate-Intensity Continuous Training on Metabolic Syndrome.	39
Silva	2011	High-intensity aerobic training improves endothelium-dependent vasodilation in patients with metabolic syndrome and type 2 diabetes mellitus	31
Siu	2015	Effects of 1-year yoga on cardiovascular risk factors in middle-aged and older adults with metabolic syndrome: A randomized trial	244
Slentz	2007	Inactivity, exercise training and detraining, and plasma lipoproteins. STRRIDE: a randomized, controlled study of exercise intensity and amount	240
Smutok	1993	Aerobic versus strength training for risk factor intervention in middle-aged men at high risk for coronary heart disease.	37
Soares	2014	Alimentary habits, physical activity, and Framingham global risk score in metabolic syndrome	70
Sonnenchein	2011	Exercise training improves in vivo endothelial repair capacity of early endothelial progenitor cells in subjects with metabolic syndrome	24
Stefanick	1998	Effects of diet and exercise in men and postmenopausal women with low levels of HDL cholesterol and high levels of LDL cholesterol.	177
Stensvold	2010	Strength training versus aerobic interval training to modify risk factors of metabolic syndrome	43
Straznicky	2009	Sympathetic Neural Adaptation to Hypocaloric Diet With or Without Exercise Training in Obese Metabolic Syndrome Subjects	59
Sun	2019	Exploration of the mechanism of improving cardiovascular function by Five Animal Play intervention in the context of general health–a case study of middle-aged men with MS	30
Sun	2015	The effect and biological mechanism of wuqinxi's intervention on middle-aged men's metabolic syndrome	30
Sykes	2004	A 12-week prospective randomized controlled trial to investigate the effects of aerobic training on type 2 diabetes patients, 2004.	36
Thompson	2010	Time course of changes in inflammatory markers during a 6-mo exercise intervention in sedentary middle-aged men: a randomized-controlled trial.	41
Thongthawee	2016	The effect of guang-im-ju-jai-gong qigong promoting program on ferric reducing antioxidant power (FRAP) anti-oxidative capacity in metabolic syndrome	55
Tjønna	2008	Aerobic interval training versus continuous moderate exercise as a treatment for the metabolic syndrome: A pilot study	28
Van	2020	Physical activity and markers of glycation in older individuals: data from a combined cross-sectional and randomized controlled trial (EXAMIN age).	158
Venojärvi	2013	Nordic walking decreased circulating chemerin and leptin concentrations in middle-aged men with impaired glucose regulation.	115
Veríssimo	2002	Effect of physical exercise on lipid metabolism in the elderly.	31
Verrusio	2016	Efficacy and safety of spinning exercise in middle- Aged and older adults with metabolic syndrome: Randomized control trial	30
Vinetti	2015	Supervised exercise training reduces oxidative stress and cardiometabolic risk in adults with type 2 diabetes: a randomized controlled trial.	20
Watkins	2003	Effects of exercise and weight loss on cardiac risk factors associated with syndrome X	41
Wedell-Neergaard	2019	Exercise-Induced changes in visceral adipose tissue mass are regulated by IL-6 signaling: a randomized controlled trial.	53
Wei	2017	A study of the effects of Baduanjin exercises on middle-aged men with metabolic syndrome	40
Yang	2023	Effect of elderly care-and -attention exercises improving metabolic syndrome index	80
Yavari	2012	Effect of aerobic exercise, resistance training or combined training on glycaemic control and cardiovascular risk factors in patients with type 2 diabetes.	80
Zou	2013	Clinical study of 100 cases of metabolic syndrome intervened by Yi Jin Jing	200

Supplementary Table 4. Summary of meta-analysis results from included reviews

References	Exercise	No. of studies (participants)	Metrics	Summary effects (95% CI)	*p*-value	I^2 (%)
**Primary outcome measures: MetS core risk factors**						
**Waist circumference (cm)**						
Cramer et al 2016	Mind-body	4 (n=320)	SMD	-0.35 (-0.57, -0.13)	0.002	0
Leme et al 2018	Aerobic	16 (n=515)	MD	-2.09 (-3.91, -0.26)	0.006	31
Leme et al 2016	Resistance	8 (n=497)	MD	-1.09 (-2.30, 0.12)	0.08	0
Li et al 2024	Mind-body	8 (n=1,105)	MD	-2.20 (-3.34, -1.06)	<0.001	60
Ostman et al 2017	Aerobic	13 (n=261)	MD	-1.37 (-2.02, -0.71)	<0.001	61
Ostman et al 2017	Combined	3 (n=652)	MD	-3.80 (-5.65, -1.95)	<0.001	59
Pattyn et al 2013	Aerobic	6 (n=184)	MD	-3.36 (-4.87, -1.84)	<0.001	35
Poon et al 2024	HIIT	13 (n=694)	MD	-4.12 (-4.71, -3.53)	<0.001	6
Serrablo et al 2020	HIIT	9 (n=480)	MD	-2.26 (-3.12, -1.40)	<0.001	49
Tao et al 2023	Mind-body	3 (n=136)	SMD	-0.67 (-1.16, -0.17)	NR	49
Wewege et al 2018	Aerobic	9 (n=504)	MD	-3.44 (-4.81, -2.07)	<0.001	0
Wewege et al 2018	Resistance	2 (n=75)	MD	-1.42 (-5.14, 2.31)	0.46	0
**Systolic blood pressure (mmHg)**						
Cramer et al 2016	Mind-body	4 (n=320)	SMD	-0.29 (-0.51, -0.07)	0.01	0
Leme et al 2018	Aerobic	15 (n=482)	MD	-5.11 (-7.36, -2.85)	<0.001	35
Leme et al 2016	Resistance	7 (n=476)	MD	-4.08 (-6.82, -1.33)	<0.01	0
Li et al 2024	Mind-body	12 (n=1,375)	MD	-3.65 (-5.56, -1.74)	<0.001	75
Ostman et al 2017	Aerobic	15 (n=364)	MD	-2.54 (-4.34, -0.75)	0.006	78
Ostman et al 2017	Combined	3 (n=652)	MD	-3.79 (-6.18, -1.40)	0.002	0
Pattyn et al 2013	Aerobic	6 (n=170)	MD	-7.11 (-9.03, -5.18)	<0.001	0
Poon et al 2024	HIIT	9 (n=446)	MD	-6.05 (-8.11, -4.00)	<0.001	19
Serrablo et al 2020	HIIT	6 (n=296)	MD	-4.44 (-6.82, -2.06)	<0.001	57
Tao et al 2023	Mind-body	5 (n=256)	SMD	-0.53 (-0.78, -0.28)	NR	0
Wewege et al 2018	Aerobic	7 (n=336)	MD	-1.94 (-4.31, 0.44)	0.11	0
Wewege et al 2018	Resistance	2 (n=72)	MD	-2.70 (-9.27, 3.87)	0.42	0
**Diastolic blood pressure (mmHg)**						
Cramer et al 2016	Mind-body	4 (n=320)	SMD	-0.20 (-0.42, 0.02)	0.07	0
Leme et al 2018	Aerobic	14 (n=449)	MD	-2.97 (-4.99, -0.94)	0.004	55
Leme et al 2016	Resistance	7 (n=476)	MD	-1.39 (-2.98, 0.19)	0.08	0
Li et al 2024	Mind-body	11 (n=1,105)	MD	-3.32 (-3.77, -2.87)	<0.001	28
Ostman et al 2017	Aerobic	14 (n=337)	MD	-2.27 (-3.47, -1.06)	<0.001	61
Ostman et al 2017	Combined	3 (n=652)	MD	-0.23 (-2.01, 1.55)	0.85	68
Pattyn et al 2013	Aerobic	6 (n=170)	MD	-5.15 (-6.16, -4.13)	<0.001	28
Poon et al 2024	HIIT	19 (n=446)	MD	-3.68 (-5.70, -1.65)	<0.001	39
Serrablo et al 2020	HIIT	6 (n=296)	MD	-3.60 (-5.43, -1.78)	<0.001	45
Tao et al 2023	Mind-body	5 (n=256)	SMD	-0.52 (-0.97, -0.06)	NR	67
Wewege et al 2018	Aerobic	7 (n=336)	MD	-1.59 (-2.86, -0.32)	0.01	0
Wewege et al 2018	Resistance	2 (n=72)	MD	-2.72 (-6.38, 0.95)	0.15	0
**High-density lipoprotein cholesterol (mmol/L)**						
Cramer et al 2016	Mind-body	4 (n=320)	SMD	0.17 (-0.36, 0.70)	0.53	76
Leme et al 2018	Aerobic	16 (n=496)	MD	0.08 (0.03, 0.13)	0.04	57
Leme et al 2016	Resistance	8 (n=497)	MD	0.00 (-0.04, 0.05)	0.91	0

Li et al 2024	Mind-body	12 (n=1,400)	SMD	0.58 (0.28, 0.87)	<0.001	84
Ostman et al 2017	Aerobic	15 (n=265)	MD	0.03 (-0.01, 0.08)	0.19	71
Ostman et al 2017	Combined	2 (n=623)	MD	0.14 (0.04, 0.25)	0.009	61
Pattyn et al 2013	Aerobic	7 (n=209)	MD	0.06 (0.03, 0.09)	<0.001	38
Poon et al 2024	HIIT	14 (n=728)	MD	0.12 (0.04, 0.20)	0.004	85
Serrablo et al 2020	HIIT	8 (n=456)	SMD	0.02 (0.00, 0.05)	0.03	61
Tao et al 2023	Mind-body	6 (n=441)	SMD	0.93 (0.19, 1.67)	NR	91
Wewege et al 2018	Aerobic	7 (n=427)	MD	0.05 (0.01, 0.08)	0.02	0
Wewege et al 2018	Resistance	2 (n=72)	MD	0.00 (-0.15, 0.15)	0.97	0
Wood et al 2021	Aerobic	41 (n=2,719)	MD	0.08 (0.05, 0.11)	<0.001	53
**Triglyceride (mmol/L)**						
Cramer et al 2016	Mind-body	4 (n=320)	SMD	-0.02 (-0.24, 0.20)	0.89	0
Leme et al 2018	Aerobic	16 (n=507)	MD	-0.09 (-0.20, 0.03)	0.13	0
Leme et al 2016	Resistance	8 (n=497)	MD	-0.03 (-0.20, 0.14)	0.75	0
Li et al 2024	Mind-body	12 (n=1,420)	SMD	-0.27 (-0.46, -0.08)	0.004	62
Ostman et al 2017	Aerobic	13 (n=308)	MD	-0.21 (-0.29, -0.13)	<0.001	57
Ostman et al 2017	Combined	1 (n=606)	MD	-0.01 (-0.04, 0.02)	0.50	0
Pattyn et al 2013	Aerobic	7 (n=209)	MD	-0.05 (-0.20, 0.09)	0.47	0
Poon et al 2024	HIIT	14 (n=728)	MD	-0.34 (-0.41, -0.27)	<0.001	55
Serrablo et al 2020	HIIT	8 (n=456)	MD	-0.01 (-0.04, 0.01)	0.32	91
Tao et al 2023	Mind-body	6 (n=441)	SMD	-0.60 (-0.79, -0.41)	NR	0
Wewege et al 2018	Aerobic	8 (n=471)	MD	-0.29 (-0.43, -0.14)	<0.001	0
Wewege et al 2018	Resistance	2 (n=72)	MD	-0.15 (-0.58, 0.28)	0.49	0
Wood et al 2021	Aerobic	36 (n=2,558)	MD	-0.17 (-0.19, -0.14)	<0.001	0
**Fasting blood glucose (mmol/L)**						
Cramer et al 2016	Mind-body	4 (n=320)	SMD	0.03 (-0.19, 0.25)	0.80	0
Leme et al 2018	Aerobic	15 (n=469)	MD	-0.08 (-0.23, 0.08)	0.33	27
Leme et al 2016	Resistance	7 (n=370)	MD	-0.04 (-0.21, 0.12)	0.60	0
Li et al 2024	Mind-body	14 (n=1,665)	SMD	-0.57 (-0.99, -0.15)	0.008	94
Ostman et al 2017	Aerobic	15 (n=378)	MD	-0.16 (-0.32, -0.01)	0.04	81
Ostman et al 2017	Combined	2 (n=623)	MD	-0.18 (-0.47, 0.25)	0.21	0
Pattyn et al 2013	Aerobic	6 (n=187)	MD	-0.31 (-0.64, 0.01)	0.06	0
Poon et al 2024	HIIT	13 (n=674)	MD	-0.35 (-0.54, -0.16)	0.004	50
Serrablo et al 2020	HIIT	8 (n=456)	MD	-0.11 (-0.16, -0.06)	<0.001	75
Tao et al 2023	Mind-body	6 (n=441)	SMD	-1.28 (-2.16, -0.40)	NR	93
Wewege et al 2018	Aerobic	8 (n=471)	MD	-0.15 (-0.29, -0.02)	0.03	0
Wewege et al 2018	Resistance	2 (n=72)	MD	0.13 (-0.17, 0.43)	0.39	10
**Secondary outcome measures: Other clinically relevant cardiometabolic health variables**						
**Body mass index (kg/m^2)**						
Li et al 2024	Mind-body	8 (n=779)	MD	-1.50 (-2.03, -0.97)	<0.001	71
Ostman et al 2017	Aerobic	14 (n=385)	MD	-0.29 (-0.44, -0.15)	<0.001	79
Ostman et al 2017	Combined	3 (n=652)	MD	-0.40 (-0.88, 0.07)	0.10	100
Poon et al 2024	HIIT	8 (n=511)	MD	-1.40 (-1.68, -1.11)	<0.001	41
Tao et al 2023	Mind-body	5 (n=365)	SMD	-0.75 (-1.17, -0.32)	NR	67
Wewege et al 2018	Aerobic	4 (n=209)	MD	-0.48 (-1.04, 0.09)	0.10	0
Pattyn et al 2013	Aerobic	7 (n=209)	MD	-1.65 (-2.81, -0.48)	<0.01	0
**Body mass (kg)**						
Ostman et al 2017	Aerobic	11 (n=217)	MD	-1.16 (-1.83, -0.48)	<0.001	43

Ostman et al 2017	Combined	2 (n=46)	MD	-0.03 (-0.51, 0.46)	0.92	24
Poon et al 2024	HIIT	9 (n=544)	MD	-4.99 (-7.96, -2.01)	0.001	41
Wewege et al 2018	Aerobic	9 (n=369)	MD	-1.64 (-2.75, -0.54)	0.04	0
Pattyn et al 2013	Aerobic	6 (n=181)	MD	-3.51 (-5.40, -1.61)	<0.001	0
**Total fat mass (kg)**						
Ostman et al 2017	Aerobic	5 (n=176)	MD	-1.19 (-1.79, -0.59)	<0.001	0
Poon et al 2024	HIIT	6 (n=403)	MD	-2.36 (-4.44, -0.28)	0.03	15
Wewege et al 2018	Aerobic	4 (n=115)	MD	-0.25 (-0.63, 0.13)	0.19	0
Pattyn et al 2013	Aerobic	3 (n=90)	MD	-1.92 (-6.41, 2.57)	NS	0
**Homeostatic model assessment for insulin resistance (unit)**						
Li et al 2024	Mind-body	3 (n=450)	MD	-0.78 (-1.13, -0.43)	<0.001	41
Poon et al 2024	HIIT	5 (n=288)	MD	-0.76 (-1.53, 0.00)	0.02	88
**Total cholesterol (mmol/L)**						
Poon et al 2024	HIIT	8 (n=416)	MD	-0.42 (-0.64, 0.19)	<0.001	83
Tao et al 2023	Mind-body	6 (n=441)	SMD	-0.42 (-0.94, 0.09)	<0.01	83
Wewege et al 2018	Aerobic	6 (n=246)	MD	-0.25 (-0.45, -0.04)	0.02	0
Wood et al 2021	Aerobic	39 (n=2,594)	MD	-0.29 (-0.36, -0.21)	<0.01	33
Pattyn et al 2013	Aerobic	5 (n=152)	MD	-0.53 (-0.64, -0.42	<0.001	23
**Low-density lipoprotein cholesterol (mmol/L)**						
Ostman et al 2017	Aerobic	2 (n=44)	MD	-0.03 (-0.05, 0.00)	0.02	0
Ostman et al 2017	Combined	1 (n=722)	MD	-0.30 (-0.61, 0.01)	0.06	0
Poon et al 2024	HIIT	8 (n=340)	MD	-0.45 (-0.68, -0.22)	<0.001	86
Tao et al 2023	Mind-body	6 (n=441)	SMD	-1.22 (-1.95, -0.50)	NR	90
Wewege et al 2018	Aerobic	6 (n=376)	MD	-0.16 (-0.37, 0.05)	0.13	37
Wood et al 2021	Aerobic	38 (n=2,566)	MD	-0.12 (-0.16, -0.09)	<0.01	0
Pattyn et al 2013	Aerobic	4 (n=130)	MD	-0.38 (-0.75, -0.01)	0.05	60
**Cardiorespiratory fitness (Peak VO_2 – mL/kg/min)**						
Ostman et al 2017	Aerobic	13 (n=294)	MD	3.00 (1.92, 4.08)	<0.001	60
Ostman et al 2017	Combined	3 (n=652)	MD	4.64 (2.42, 6.87)	<0.001	48
Poon et al 2024	HIIT	4 (n=97)	MD	8.61 (6.05, 11.18)	<0.001	6
Pattyn et al 2013	Aerobic	6 (n=181)	MD	5.86 (3.03, 8.69)	<0.001	58
**MetS z-score (unit)**						
Poon et al 2024	HIIT	3 (n=248)	MD	-0.27 (-0.42, 0.11)	<0.001	0
Yin et al 2024	LV-HIIT	4 (n=221)	SMD	-0.76 (-1.02, -0.49)	<0.01	0

HIIT: High-intensity interval training, LV-HIIT: Low-volume high-intensity interval training, MD: mean difference, SMD: Standardized mean difference

Yoga for metabolic syndrome: A systematic review and meta-analysis
(Cramer et al., 2016)

AMSTAR 2 **(Rating: Critically Low)**

1. Did the research questions and inclusion criteria for the review include the components of PICO?

For Yes:	Optional (recommended)	
☑ Population<br>☑ Intervention<br>☑ Comparator group<br>☑ Outcome	☐ Timeframe for follow-up	☑ Yes<br>☐ No

2. Did the report of the review contain an explicit statement that the review methods were established prior to the conduct of the review and did the report justify any significant deviations from the protocol?

For Partial Yes:	For Yes:	
The authors state that they had a written protocol or guide that included ALL the following:<br>☒ review question(s)<br>☐ a search strategy<br>☐ inclusion/exclusion criteria<br>☐ a risk of bias assessment	As for partial yes, plus the protocol should be registered and should also have specified:<br>☐ a meta-analysis/synthesis plan, if appropriate, *and*<br>☐ a plan for investigating causes of heterogeneity<br>☐ justification for any deviations from the protocol	☐ Yes<br>☐ Partial Yes<br>☑ No

3. Did the review authors explain their selection of the study designs for inclusion in the review?

For Yes, the review should satisfy ONE of the following:	
☑ *Explanation for* including only RCTs<br>☐ OR *Explanation for* including only NRSI<br>☐ OR *Explanation for* including both RCTs and NRSI	☑ Yes<br>☐ No

4. Did the review authors use a comprehensive literature search strategy?

For Partial Yes (all the following):	For Yes, should also have (all the following):	
☑ searched at least 2 databases (relevant to research question)<br>☑ provided key word and/or search strategy<br>☑ justified publication restrictions (eg, language)	☑ searched the reference lists/bibliographies of included studies<br>☒ searched trial/study registries<br>☐ included/consulted content experts in the field<br>☐ where relevant, searched for grey literature<br>☐ conducted search within 24 months of completion of the review	☐ Yes<br>☑ Partial Yes<br>☐ No

5. Did the review authors perform study selection in duplicate?

For Yes, either ONE of the following:	
☑ at least two reviewers independently agreed on selection of eligible studies and achieved consensus on which studies to include<br>☐ OR two reviewers selected a sample of eligible studies and achieved good agreement (at least 80 per cent), with the remainder selected by one reviewer	☑ Yes<br>☐ No

6. Did the review authors perform data extraction in duplicate?

For Yes, either ONE of the following:	
☑ at least two reviewers achieved consensus on which data to extract	☑ Yes

from included studies<br>☐ OR two reviewers extracted data from a sample of eligible studies <u>and</u> achieved good agreement (at least 80 per cent), with the remainder extracted by one reviewer		☐ No

7. Did the review authors provide a list of excluded studies and justify the exclusions?

For Partial Yes:	For Yes, must also have:	
☑ provided a list of all potentially relevant studies that were read in full text form but excluded from the review	☑ Justified the exclusion from the review of each potentially relevant study	☑ Yes<br>☐ Partial Yes<br>☐ No

8. Did the review authors describe the included studies in adequate detail?

For Partial Yes (ALL the following):	For Yes, should also have ALL the following:	
☑ described populations<br>☑ described interventions<br>☑ described comparators<br>☑ described outcomes<br>☑ described research designs	☑ described population in detail<br>☑ described intervention and comparator in detail (including doses where relevant)<br>☑ described study's setting<br>☑ timeframe for follow-up	☑ Yes<br>☐ Partial Yes<br>☐ No

9. Did the review authors use a satisfactory technique for assessing the risk of bias (RoB) in individual studies that were included in the review?

**RCTs**<br>For Partial Yes, must have assessed RoB from	For Yes, must also have assessed RoB from:	
☑ unconcealed allocation, *and*<br>☑ lack of blinding of patients and assessors when assessing outcomes (unnecessary for objective outcomes such as all cause mortality)	☑ allocation sequence that was not truly random, *and*<br>☑ selection of the reported result from among multiple measurements or analyses of a specified outcome	☑ Yes<br>☐ Partial Yes<br>☐ No<br>☐ Includes only NRSI
**NRSI**<br>For Partial Yes, must have assessed RoB:	For Yes, must also have assessed RoB:	
☐ from confounding, *and*<br>☐ from selection bias	☐ methods used to ascertain exposures and outcomes, *and*<br>☐ selection of the reported result from among multiple measurements or analyses of a specified outcome	☐ Yes<br>☐ Partial Yes<br>☐ No<br>☑ Includes only RCTs

10. Did the review authors report on the sources of funding for the studies included in the review?

For Yes	
☒ Must have reported on the sources of funding for individual studies included in the review. Note: Reporting that the reviewers looked for this information but it was not reported by study authors also qualifies	☐ Yes<br>☑ No

11. If meta-analysis was performed did the review authors use appropriate methods for statistical combination of results?

**RCTs**<br>For Yes:	
☑ The authors justified combining the data in a meta-analysis<br>☑ AND they used an appropriate weighted technique to combine study results and adjusted for heterogeneity if present	☐ Yes<br>☑ No<br>☐ No meta-analysis

☒ AND investigated the causes of any heterogeneity	conducted
**For NRSI**<br>For Yes:<br>☐ The authors justified combining the data in a meta-analysis<br>☐ AND they used an appropriate weighted technique to combine study results, adjusting for heterogeneity if present<br>☐ AND they statistically combined effect estimates from NRSI that were adjusted for confounding, rather than combining raw data, or justified combining raw data when adjusted effect estimates were not available<br>☐ AND they reported separate summary estimates for RCTs and NRSI separately when both were included in the review	☐ Yes<br>☐ No<br>☐ No meta-analysis conducted
**12. If meta-analysis was performed, did the review authors assess the potential impact of RoB in individual studies on the results of the meta-analysis or other evidence synthesis?**	
For Yes:<br>☐ included only low risk of bias RCTs<br>☑ OR, if the pooled estimate was based on RCTs and/or NRSI at variable RoB, the authors performed analyses to investigate possible impact of RoB on summary estimates of effect	☑ Yes<br>☐ No<br>☐ No meta-analysis conducted
**13. Did the review authors account for RoB in individual studies when interpreting/discussing the results of the review?**	
For Yes:<br>☐ included only low risk of bias RCTs<br>☑ OR, if RCTs with moderate or high RoB, or NRSI were included the review provided a discussion of the likely impact of RoB on the results	☑ Yes<br>☐ No
**14. Did the review authors provide a satisfactory explanation for, and discussion of, any heterogeneity observed in the results of the review?**	
For Yes:<br>☐ There was no significant heterogeneity in the results<br>☒ OR if heterogeneity was present the authors performed an investigation of sources of any heterogeneity in the results and discussed the impact of this on the results of the review	☐ Yes<br>☑ No
**15. If they performed quantitative synthesis did the review authors carry out an adequate investigation of publication bias (small study bias) and discuss its likely impact on the results of the review?**	
For Yes:<br>☒ performed graphical or statistical tests for publication bias and discussed the likelihood and magnitude of impact of publication bias	☐ Yes<br>☑ No<br>☐ No meta-analysis conducted
**16. Did the review authors report any potential sources of conflict of interest, including any funding they received for conducting the review?**	
For Yes:<br>☐ The authors reported no competing interests OR<br>☑ The authors described their funding sources and how they managed potential conflicts of interest	☑ Yes<br>☐ No

Aerobic training reduces blood pressure and waist circumference, and increases HDL-c in metabolic syndrome: a systematic review and meta-analysis of randomized controlled trials
(Leme et al., 2018)

AMSTAR 2 **(Rating: Moderate)**

1. Did the research questions and inclusion criteria for the review include the components of PICO?

For Yes:	Optional (recommended)	
☑ Population<br>☑ Intervention<br>☑ Comparator group<br>☑ Outcome	☐ Timeframe for follow-up	☑ Yes<br>☐ No

2. Did the report of the review contain an explicit statement that the review methods were established prior to the conduct of the review and did the report justify any significant deviations from the protocol?

For Partial Yes: The authors state that they had a written protocol or guide that included ALL the following:	For Yes: As for partial yes, plus the protocol should be registered and should also have specified:	
☑ review question(s)<br>☑ a search strategy<br>☑ inclusion/exclusion criteria<br>☑ a risk of bias assessment	☑ a meta-analysis/synthesis plan, if appropriate, *and*<br>☒ a plan for investigating causes of heterogeneity<br>☐ justification for any deviations from the protocol	☐ Yes<br>☑ Partial Yes<br>☐ No

3. Did the review authors explain their selection of the study designs for inclusion in the review?

For Yes, the review should satisfy ONE of the following:	
☑ *Explanation for* including only RCTs<br>☐ OR *Explanation for* including only NRSI<br>☐ OR *Explanation for* including both RCTs and NRSI	☑ Yes<br>☐ No

4. Did the review authors use a comprehensive literature search strategy?

For Partial Yes (all the following):	For Yes, should also have (all the following):	
☑ searched at least 2 databases (relevant to research question)<br>☑ provided key word and/or search strategy<br>☑ justified publication restrictions (eg, language)	☑ searched the reference lists/bibliographies of included studies<br>☒ searched trial/study registries<br>☐ included/consulted content experts in the field<br>☐ where relevant, searched for grey literature<br>☐ conducted search within 24 months of completion of the review	☐ Yes<br>☑ Partial Yes<br>☐ No

5. Did the review authors perform study selection in duplicate?

For Yes, either ONE of the following:	
☑ at least two reviewers independently agreed on selection of eligible studies and achieved consensus on which studies to include<br>☐ OR two reviewers selected a sample of eligible studies and achieved good agreement (at least 80 per cent), with the remainder selected by one reviewer	☑ Yes<br>☐ No

6. Did the review authors perform data extraction in duplicate?

For Yes, either ONE of the following:	
☒ at least two reviewers achieved consensus on which data to extract	☐ Yes

from included studies
☐ OR two reviewers extracted data from a sample of eligible studies and achieved good agreement (at least 80 per cent), with the remainder extracted by one reviewer

☑ No

7. Did the review authors provide a list of excluded studies and justify the exclusions?

For Partial Yes:
☒ provided a list of all potentially relevant studies that were read in full text form but excluded from the review

For Yes, must also have:
☐ Justified the exclusion from the review of each potentially relevant study

☐ Yes
☐ Partial Yes
☑ No

8. Did the review authors describe the included studies in adequate detail?

For Partial Yes (ALL the following):
☑ described populations
☑ described interventions
☑ described comparators
☑ described outcomes
☑ described research designs

For Yes, should also have ALL the following:
☑ described population in detail
☑ described intervention and comparator in detail (including doses where relevant)
☒ described study's setting
☑ timeframe for follow-up

☐ Yes
☑ Partial Yes
☐ No

9. Did the review authors use a satisfactory technique for assessing the risk of bias (RoB) in individual studies that were included in the review?

RCTs

For Partial Yes, must have assessed RoB from
☑ unconcealed allocation, *and*
☑ lack of blinding of patients and assessors when assessing outcomes (unnecessary for objective outcomes such as all cause mortality)

For Yes, must also have assessed RoB from:
☐ allocation sequence that was not truly random, *and*
☒ selection of the reported result from among multiple measurements or analyses of a specified outcome

☐ Yes
☑ Partial Yes
☐ No
☐ Includes only NRSI

NRSI

For Partial Yes, must have assessed RoB:
☐ from confounding, *and*
☐ from selection bias

For Yes, must also have assessed RoB:
☐ methods used to ascertain exposures and outcomes, *and*
☐ selection of the reported result from among multiple measurements or analyses of a specified outcome

☐ Yes
☐ Partial Yes
☐ No
☑ Includes only RCTs

10. Did the review authors report on the sources of funding for the studies included in the review?

For Yes
☒ Must have reported on the sources of funding for individual studies included in the review. Note: Reporting that the reviewers looked for this information but it was not reported by study authors also qualifies

☐ Yes
☑ No

11. If meta-analysis was performed did the review authors use appropriate methods for statistical combination of results?

RCTs

For Yes:
☑ The authors justified combining the data in a meta-analysis
☑ AND they used an appropriate weighted technique to combine study results and adjusted for heterogeneity if present

☑ Yes
☐ No
☐ No meta-analysis

☑ AND investigated the causes of any heterogeneity	conducted
**For NRSI**<br>For Yes:<br>☐ The authors justified combining the data in a meta-analysis<br>☐ AND they used an appropriate weighted technique to combine study results, adjusting for heterogeneity if present<br>☐ AND they statistically combined effect estimates from NRSI that were adjusted for confounding, rather than combining raw data, or justified combining raw data when adjusted effect estimates were not available<br>☐ AND they reported separate summary estimates for RCTs and NRSI separately when both were included in the review	☐ Yes<br>☐ No<br>☐ No meta-analysis conducted

12. If meta-analysis was performed, did the review authors assess the potential impact of RoB in individual studies on the results of the meta-analysis or other evidence synthesis?

For Yes:<br>☐ included only low risk of bias RCTs<br>☑ OR, if the pooled estimate was based on RCTs and/or NRSI at variable RoB, the authors performed analyses to investigate possible impact of RoB on summary estimates of effect	☑ Yes<br>☐ No<br>☐ No meta-analysis conducted

13. Did the review authors account for RoB in individual studies when interpreting/discussing the results of the review?

For Yes:<br>☐ included only low risk of bias RCTs<br>☑ OR, if RCTs with moderate or high RoB, or NRSI were included the review provided a discussion of the likely impact of RoB on the results	☑ Yes<br>☐ No

14. Did the review authors provide a satisfactory explanation for, and discussion of, any heterogeneity observed in the results of the review?

For Yes:<br>☐ There was no significant heterogeneity in the results<br>☑ OR if heterogeneity was present the authors performed an investigation of sources of any heterogeneity in the results and discussed the impact of this on the results of the review	☑ Yes<br>☐ No

15. If they performed quantitative synthesis did the review authors carry out an adequate investigation of publication bias (small study bias) and discuss its likely impact on the results of the review?

For Yes:<br>☑ performed graphical or statistical tests for publication bias and discussed the likelihood and magnitude of impact of publication bias	☑ Yes<br>☐ No<br>☐ No meta-analysis conducted

16. Did the review authors report any potential sources of conflict of interest, including any funding they received for conducting the review?

For Yes:<br>☑ The authors reported no competing interests OR<br>☐ The authors described their funding sources and how they managed potential conflicts of interest	☑ Yes<br>☐ No

Resistance training reduces systolic blood pressure in metabolic syndrome: a systematic review and meta-analysis of randomised controlled trials
(Leme et al., 2016)

AMSTAR 2 **(Rating: Low)**

1. Did the research questions and inclusion criteria for the review include the components of PICO?

For Yes:	Optional (recommended)	
☑ Population	☐ Timeframe for follow-up	☑ Yes
☑ Intervention		☐ No
☑ Comparator group		
☑ Outcome		

2. Did the report of the review contain an explicit statement that the review methods were established prior to the conduct of the review and did the report justify any significant deviations from the protocol?

For Partial Yes: The authors state that they had a written protocol or guide that included ALL the following:	For Yes: As for partial yes, plus the protocol should be registered and should also have specified:	
		☐ Yes
☑ review question(s)	☑ a meta-analysis/synthesis plan, if appropriate, *and*	☑ Partial Yes
☑ a search strategy		☐ No
☑ inclusion/exclusion criteria	☒ a plan for investigating causes of heterogeneity	
☑ a risk of bias assessment	☐ justification for any deviations from the protocol	

3. Did the review authors explain their selection of the study designs for inclusion in the review?

For Yes, the review should satisfy ONE of the following:	
☑ *Explanation for* including only RCTs	☑ Yes
☐ OR *Explanation for* including only NRSI	☐ No
☐ OR *Explanation for* including both RCTs and NRSI	

4. Did the review authors use a comprehensive literature search strategy?

For Partial Yes (all the following):	For Yes, should also have (all the following):	
☑ searched at least 2 databases (relevant to research question)	☑ searched the reference lists/bibliographies of included studies	☐ Yes
☑ provided key word and/or search strategy	☒ searched trial/study registries	☑ Partial Yes
☑ justified publication restrictions (eg, language)	☐ included/consulted content experts in the field	☐ No
	☐ where relevant, searched for grey literature	
	☐ conducted search within 24 months of completion of the review	

5. Did the review authors perform study selection in duplicate?

For Yes, either ONE of the following:	
☑ at least two reviewers independently agreed on selection of eligible studies and achieved consensus on which studies to include	☑ Yes
☐ OR two reviewers selected a sample of eligible studies <u>and</u> achieved good agreement (at least 80 per cent), with the remainder selected by one reviewer	☐ No

6. Did the review authors perform data extraction in duplicate?

For Yes, either ONE of the following:	
☑ at least two reviewers achieved consensus on which data to extract	☑ Yes

from included studies
☐ OR two reviewers extracted data from a sample of eligible studies and achieved good agreement (at least 80 per cent), with the remainder extracted by one reviewer

☐ No

7. Did the review authors provide a list of excluded studies and justify the exclusions?

For Partial Yes:	For Yes, must also have:	
☑ provided a list of all potentially relevant studies that were read in full text form but excluded from the review	☑ Justified the exclusion from the review of each potentially relevant study	☑ Yes<br>☐ Partial Yes<br>☐ No

8. Did the review authors describe the included studies in adequate detail?

For Partial Yes (ALL the following):	For Yes, should also have ALL the following:	
☑ described populations<br>☑ described interventions<br>☑ described comparators<br>☑ described outcomes<br>☑ described research designs	☑ described population in detail<br>☑ described intervention and comparator in detail (including doses where relevant)<br>☐ described study's setting<br>☑ timeframe for follow-up	☐ Yes<br>☑ Partial Yes<br>☐ No

9. Did the review authors use a satisfactory technique for assessing the risk of bias (RoB) in individual studies that were included in the review?

RCTs

For Partial Yes, must have assessed RoB from	For Yes, must also have assessed RoB from:	
☑ unconcealed allocation, *and*<br>☑ lack of blinding of patients and assessors when assessing outcomes (unnecessary for objective outcomes such as all cause mortality)	☑ allocation sequence that was not truly random, *and*<br>☑ selection of the reported result from among multiple measurements or analyses of a specified outcome	☑ Yes<br>☐ Partial Yes<br>☐ No<br>☐ Includes only NRSI

NRSI

For Partial Yes, must have assessed RoB:	For Yes, must also have assessed RoB:	
☐ from confounding, *and*<br>☐ from selection bias	☐ methods used to ascertain exposures and outcomes, *and*<br>☐ selection of the reported result from among multiple measurements or analyses of a specified outcome	☐ Yes<br>☐ Partial Yes<br>☐ No<br>☑ Includes only RCTs

10. Did the review authors report on the sources of funding for the studies included in the review?

For Yes

☒ Must have reported on the sources of funding for individual studies included in the review. Note: Reporting that the reviewers looked for this information but it was not reported by study authors also qualifies

☐ Yes
☑ No

11. If meta-analysis was performed did the review authors use appropriate methods for statistical combination of results?

RCTs

For Yes:

☑ The authors justified combining the data in a meta-analysis
☑ AND they used an appropriate weighted technique to combine study results and adjusted for heterogeneity if present

☑ Yes
☐ No
☐ No meta-analysis

☑ AND investigated the causes of any heterogeneity — conducted

For NRSI

For Yes:

- ☐ The authors justified combining the data in a meta-analysis
  - ☐ AND they used an appropriate weighted technique to combine study results, adjusting for heterogeneity if present
  - ☐ AND they statistically combined effect estimates from NRSI that were adjusted for confounding, rather than combining raw data, or justified combining raw data when adjusted effect estimates were not available
  - ☐ AND they reported separate summary estimates for RCTs and NRSI separately when both were included in the review

☐ Yes
☐ No
☐ No meta-analysis conducted

12. If meta-analysis was performed, did the review authors assess the potential impact of RoB in individual studies on the results of the meta-analysis or other evidence synthesis?

For Yes:

- ☐ included only low risk of bias RCTs
- ☑ OR, if the pooled estimate was based on RCTs and/or NRSI at variable RoB, the authors performed analyses to investigate possible impact of RoB on summary estimates of effect

☑ Yes
☐ No
☐ No meta-analysis conducted

13. Did the review authors account for RoB in individual studies when interpreting/discussing the results of the review?

For Yes:

- ☐ included only low risk of bias RCTs
- ☑ OR, if RCTs with moderate or high RoB, or NRSI were included the review provided a discussion of the likely impact of RoB on the results

☑ Yes
☐ No

14. Did the review authors provide a satisfactory explanation for, and discussion of, any heterogeneity observed in the results of the review?

For Yes:

- ☐ There was no significant heterogeneity in the results
- ☑ OR if heterogeneity was present the authors performed an investigation of sources of any heterogeneity in the results and discussed the impact of this on the results of the review

☑ Yes
☐ No

15. If they performed quantitative synthesis did the review authors carry out an adequate investigation of publication bias (small study bias) and discuss its likely impact on the results of the review?

For Yes:

- ☒ performed graphical or statistical tests for publication bias and discussed the likelihood and magnitude of impact of publication bias

☐ Yes
☑ No
☐ No meta-analysis conducted

16. Did the review authors report any potential sources of conflict of interest, including any funding they received for conducting the review?

For Yes:

- ☐ The authors reported no competing interests OR
- ☑ The authors described their funding sources and how they managed potential conflicts of interest

☑ Yes
☐ No

Effect of mind-body exercise on risk factors for metabolic syndrome including insulin resistance: a meta-analysis
(Li et al., 2024)

AMSTAR 2 **(Rating: Critically Low)**

1. Did the research questions and inclusion criteria for the review include the components of PICO?

For Yes:	Optional (recommended)	
☑ Population<br>☑ Intervention<br>☑ Comparator group<br>☑ Outcome	☐ Timeframe for follow-up	☑ Yes<br>☐ No

2. Did the report of the review contain an explicit statement that the review methods were established prior to the conduct of the review and did the report justify any significant deviations from the protocol?

For Partial Yes:<br>The authors state that they had a written protocol or guide that included ALL the following:	For Yes:<br>As for partial yes, plus the protocol should be registered and should also have specified:	
☑ review question(s)<br>☑ a search strategy<br>☑ inclusion/exclusion criteria<br>☑ a risk of bias assessment	☑ a meta-analysis/synthesis plan, if appropriate, *and*<br>☑ a plan for investigating causes of heterogeneity<br>☑ justification for any deviations from the protocol	☑ Yes<br>☐ Partial Yes<br>☐ No

3. Did the review authors explain their selection of the study designs for inclusion in the review?

For Yes, the review should satisfy ONE of the following:	
☑ *Explanation for* including only RCTs<br>☐ OR *Explanation for* including only NRSI<br>☐ OR *Explanation for* including both RCTs and NRSI	☑ Yes<br>☐ No

4. Did the review authors use a comprehensive literature search strategy?

For Partial Yes (all the following):	For Yes, should also have (all the following):	
☑ searched at least 2 databases (relevant to research question)<br>☑ provided key word and/or search strategy<br>☑ justified publication restrictions (eg, language)	☒ searched the reference lists/bibliographies of included studies<br>☐ searched trial/study registries<br>☐ included/consulted content experts in the field<br>☐ where relevant, searched for grey literature<br>☐ conducted search within 24 months of completion of the review	☐ Yes<br>☑ Partial Yes<br>☐ No

5. Did the review authors perform study selection in duplicate?

For Yes, either ONE of the following:	
☑ at least two reviewers independently agreed on selection of eligible studies and achieved consensus on which studies to include<br>☐ OR two reviewers selected a sample of eligible studies and achieved good agreement (at least 80 per cent), with the remainder selected by one reviewer	☑ Yes<br>☐ No

6. Did the review authors perform data extraction in duplicate?

For Yes, either ONE of the following:	
☑ at least two reviewers achieved consensus on which data to extract	☑ Yes

from included studies<br>☐ OR two reviewers extracted data from a sample of eligible studies and achieved good agreement (at least 80 per cent), with the remainder extracted by one reviewer		☐ No

7. Did the review authors provide a list of excluded studies and justify the exclusions?

For Partial Yes:	For Yes, must also have:	
☒ provided a list of all potentially relevant studies that were read in full text form but excluded from the review	☐ Justified the exclusion from the review of each potentially relevant study	☐ Yes<br>☐ Partial Yes<br>☑ No

8. Did the review authors describe the included studies in adequate detail?

For Partial Yes (ALL the following):	For Yes, should also have ALL the following:	
☑ described populations<br>☑ described interventions<br>☑ described comparators<br>☑ described outcomes<br>☑ described research designs	☑ described population in detail<br>☑ described intervention and comparator in detail (including doses where relevant)<br>☑ described study's setting<br>☑ timeframe for follow-up	☑ Yes<br>☐ Partial Yes<br>☐ No

9. Did the review authors use a satisfactory technique for assessing the risk of bias (RoB) in individual studies that were included in the review?

**RCTs**<br>For Partial Yes, must have assessed RoB from<br>☑ unconcealed allocation, *and*<br>☑ lack of blinding of patients and assessors when assessing outcomes (unnecessary for objective outcomes such as all cause mortality)	For Yes, must also have assessed RoB from:<br>☑ allocation sequence that was not truly random, *and*<br>☑ selection of the reported result from among multiple measurements or analyses of a specified outcome	☑ Yes<br>☐ Partial Yes<br>☐ No<br>☐ Includes only NRSI
**NRSI**<br>For Partial Yes, must have assessed RoB:<br>☐ from confounding, *and*<br>☐ from selection bias	For Yes, must also have assessed RoB:<br>☐ methods used to ascertain exposures and outcomes, *and*<br>☐ selection of the reported result from among multiple measurements or analyses of a specified outcome	☐ Yes<br>☐ Partial Yes<br>☐ No<br>☑ Includes only RCTs

10. Did the review authors report on the sources of funding for the studies included in the review?

For Yes	
☒ Must have reported on the sources of funding for individual studies included in the review. Note: Reporting that the reviewers looked for this information but it was not reported by study authors also qualifies	☐ Yes<br>☑ No

11. If meta-analysis was performed did the review authors use appropriate methods for statistical combination of results?

**RCTs**<br>For Yes:	
☑ The authors justified combining the data in a meta-analysis<br>☒ AND they used an appropriate weighted technique to combine study results and adjusted for heterogeneity if present	☐ Yes<br>☑ No<br>☐ No meta-analysis

☐ AND investigated the causes of any heterogeneity	conducted
**For NRSI**<br>For Yes:<br>☐ The authors justified combining the data in a meta-analysis<br>☐ AND they used an appropriate weighted technique to combine study results, adjusting for heterogeneity if present<br>☐ AND they statistically combined effect estimates from NRSI that were adjusted for confounding, rather than combining raw data, or justified combining raw data when adjusted effect estimates were not available<br>☐ AND they reported separate summary estimates for RCTs and NRSI separately when both were included in the review	☐ Yes<br>☐ No<br>☐ No meta-analysis conducted

12. If meta-analysis was performed, did the review authors assess the potential impact of RoB in individual studies on the results of the meta-analysis or other evidence synthesis?

For Yes:<br>☐ included only low risk of bias RCTs<br>☒ OR, if the pooled estimate was based on RCTs and/or NRSI at variable RoB, the authors performed analyses to investigate possible impact of RoB on summary estimates of effect	☐ Yes<br>☑ No<br>☐ No meta-analysis conducted

13. Did the review authors account for RoB in individual studies when interpreting/discussing the results of the review?

For Yes:<br>☐ included only low risk of bias RCTs<br>☒ OR, if RCTs with moderate or high RoB, or NRSI were included the review provided a discussion of the likely impact of RoB on the results	☐ Yes<br>☑ No

14. Did the review authors provide a satisfactory explanation for, and discussion of, any heterogeneity observed in the results of the review?

For Yes:<br>☐ There was no significant heterogeneity in the results<br>☒ OR if heterogeneity was present the authors performed an investigation of sources of any heterogeneity in the results and discussed the impact of this on the results of the review	☐ Yes<br>☑ No

15. If they performed quantitative synthesis did the review authors carry out an adequate investigation of publication bias (small study bias) and discuss its likely impact on the results of the review?

For Yes:<br>☑ performed graphical or statistical tests for publication bias and discussed the likelihood and magnitude of impact of publication bias	☑ Yes<br>☐ No<br>☐ No meta-analysis conducted

16. Did the review authors report any potential sources of conflict of interest, including any funding they received for conducting the review?

For Yes:<br>☐ The authors reported no competing interests OR<br>☑ The authors described their funding sources and how they managed potential conflicts of interest	☑ Yes<br>☐ No

The effect of exercise training on clinical outcomes in patients with the metabolic syndrome: a systematic review and meta-analysis
(Ostman et al., 2017)

AMSTAR 2 **(Rating: Low)**

1. Did the research questions and inclusion criteria for the review include the components of PICO?

For Yes:
- ☑ Population
- ☑ Intervention
- ☑ Comparator group
- ☑ Outcome

Optional (recommended)
- ☐ Timeframe for follow-up

- ☑ Yes
- ☐ No

2. Did the report of the review contain an explicit statement that the review methods were established prior to the conduct of the review and did the report justify any significant deviations from the protocol?

For Partial Yes:
The authors state that they had a written protocol or guide that included ALL the following:
- ☑ review question(s)
- ☑ a search strategy
- ☑ inclusion/exclusion criteria
- ☑ a risk of bias assessment

For Yes:
As for partial yes, plus the protocol should be registered and should also have specified:
- ☑ a meta-analysis/synthesis plan, if appropriate, *and*
- ☑ a plan for investigating causes of heterogeneity
- ☑ justification for any deviations from the protocol

- ☑ Yes
- ☐ Partial Yes
- ☐ No

3. Did the review authors explain their selection of the study designs for inclusion in the review?

For Yes, the review should satisfy ONE of the following:
- ☑ *Explanation for* including only RCTs
- ☐ OR *Explanation for* including only NRSI
- ☐ OR *Explanation for* including both RCTs and NRSI

- ☑ Yes
- ☐ No

4. Did the review authors use a comprehensive literature search strategy?

For Partial Yes (all the following):
- ☑ searched at least 2 databases (relevant to research question)
- ☑ provided key word and/or search strategy
- ☑ justified publication restrictions (eg, language)

For Yes, should also have (all the following):
- ☑ searched the reference lists/bibliographies of included studies
- ☒ searched trial/study registries
- ☐ included/consulted content experts in the field
- ☐ where relevant, searched for grey literature
- ☐ conducted search within 24 months of completion of the review

- ☐ Yes
- ☑ Partial Yes
- ☐ No

5. Did the review authors perform study selection in duplicate?

For Yes, either ONE of the following:
- ☑ at least two reviewers independently agreed on selection of eligible studies and achieved consensus on which studies to include
- ☐ OR two reviewers selected a sample of eligible studies and achieved good agreement (at least 80 per cent), with the remainder selected by one reviewer

- ☑ Yes
- ☐ No

6. Did the review authors perform data extraction in duplicate?

For Yes, either ONE of the following:
- ☑ at least two reviewers achieved consensus on which data to extract

- ☑ Yes

from included studies
☐ OR two reviewers extracted data from a sample of eligible studies and achieved good agreement (at least 80 per cent), with the remainder extracted by one reviewer

☐ No

7. Did the review authors provide a list of excluded studies and justify the exclusions?

For Partial Yes:
☒ provided a list of all potentially relevant studies that were read in full text form but excluded from the review

For Yes, must also have:
☐ Justified the exclusion from the review of each potentially relevant study

☐ Yes
☐ Partial Yes
☑ No

8. Did the review authors describe the included studies in adequate detail?

For Partial Yes (ALL the following):
☑ described populations
☑ described interventions
☑ described comparators
☑ described outcomes
☑ described research designs

For Yes, should also have ALL the following:
☑ described population in detail
☑ described intervention and comparator in detail (including doses where relevant)
☑ described study's setting
☑ timeframe for follow-up

☑ Yes
☐ Partial Yes
☐ No

9. Did the review authors use a satisfactory technique for assessing the risk of bias (RoB) in individual studies that were included in the review?

RCTs
For Partial Yes, must have assessed RoB from
☑ unconcealed allocation, *and*
☑ lack of blinding of patients and assessors when assessing outcomes (unnecessary for objective outcomes such as all cause mortality)

For Yes, must also have assessed RoB from:
☑ allocation sequence that was not truly random, *and*
☑ selection of the reported result from among multiple measurements or analyses of a specified outcome

☑ Yes
☐ Partial Yes
☐ No
☐ Includes only NRSI

NRSI
For Partial Yes, must have assessed RoB:
☐ from confounding, *and*
☐ from selection bias

For Yes, must also have assessed RoB:
☐ methods used to ascertain exposures and outcomes, *and*
☐ selection of the reported result from among multiple measurements or analyses of a specified outcome

☐ Yes
☐ Partial Yes
☐ No
☑ Includes only RCTs

10. Did the review authors report on the sources of funding for the studies included in the review?

For Yes
☒ Must have reported on the sources of funding for individual studies included in the review. Note: Reporting that the reviewers looked for this information but it was not reported by study authors also qualifies

☐ Yes
☑ No

11. If meta-analysis was performed did the review authors use appropriate methods for statistical combination of results?

RCTs
For Yes:
☑ The authors justified combining the data in a meta-analysis
☑ AND they used an appropriate weighted technique to combine study results and adjusted for heterogeneity if present

☑ Yes
☐ No
☐ No meta-analysis

☑ AND investigated the causes of any heterogeneity	conducted
**For NRSI**<br>For Yes:<br>☐ The authors justified combining the data in a meta-analysis<br>☐ AND they used an appropriate weighted technique to combine study results, adjusting for heterogeneity if present<br>☐ AND they statistically combined effect estimates from NRSI that were adjusted for confounding, rather than combining raw data, or justified combining raw data when adjusted effect estimates were not available<br>☐ AND they reported separate summary estimates for RCTs and NRSI separately when both were included in the review	☐ Yes<br>☐ No<br>☐ No meta-analysis conducted
**12. If meta-analysis was performed, did the review authors assess the potential impact of RoB in individual studies on the results of the meta-analysis or other evidence synthesis?**	
For Yes:<br>☒ included only low risk of bias RCTs<br>☐ OR, if the pooled estimate was based on RCTs and/or NRSI at variable RoB, the authors performed analyses to investigate possible impact of RoB on summary estimates of effect	☐ Yes<br>☑ No<br>☐ No meta-analysis conducted
**13. Did the review authors account for RoB in individual studies when interpreting/discussing the results of the review?**	
For Yes:<br>☒ included only low risk of bias RCTs<br>☐ OR, if RCTs with moderate or high RoB, or NRSI were included the review provided a discussion of the likely impact of RoB on the results	☐ Yes<br>☑ No
**14. Did the review authors provide a satisfactory explanation for, and discussion of, any heterogeneity observed in the results of the review?**	
For Yes:<br>☐ There was no significant heterogeneity in the results<br>☒ OR if heterogeneity was present the authors performed an investigation of sources of any heterogeneity in the results and discussed the impact of this on the results of the review	☐ Yes<br>☑ No
**15. If they performed quantitative synthesis did the review authors carry out an adequate investigation of publication bias (small study bias) and discuss its likely impact on the results of the review?**	
For Yes:<br>☑ performed graphical or statistical tests for publication bias and discussed the likelihood and magnitude of impact of publication bias	☑ Yes<br>☐ No<br>☐ No meta-analysis conducted
**16. Did the review authors report any potential sources of conflict of interest, including any funding they received for conducting the review?**	
For Yes:<br>☑ The authors reported no competing interests OR<br>☐ The authors described their funding sources and how they managed potential conflicts of interest	☑ Yes<br>☐ No

The Effect of Exercise on the Cardiovascular Risk Factors Constituting the Metabolic Syndrome
(Pattyn et al., 2013)

AMSTAR 2 **(Rating: Critically Low)**

1. Did the research questions and inclusion criteria for the review include the components of PICO?

For Yes:
- ☑ Population
- ☑ Intervention
- ☒ Comparator group
- ☑ Outcome

Optional (recommended)
- ☐ Timeframe for follow-up

- ☐ Yes
- ☑ No

2. Did the report of the review contain an explicit statement that the review methods were established prior to the conduct of the review and did the report justify any significant deviations from the protocol?

For Partial Yes:
The authors state that they had a written protocol or guide that included ALL the following:
- ☒ review question(s)
- ☐ a search strategy
- ☐ inclusion/exclusion criteria
- ☐ a risk of bias assessment

For Yes:
As for partial yes, plus the protocol should be registered and should also have specified:
- ☒ a meta-analysis/synthesis plan, if appropriate, *and*
- ☐ a plan for investigating causes of heterogeneity
- ☐ justification for any deviations from the protocol

- ☐ Yes
- ☐ Partial Yes
- ☑ No

3. Did the review authors explain their selection of the study designs for inclusion in the review?

For Yes, the review should satisfy ONE of the following:
- ☐ *Explanation for* including only RCTs
- ☐ OR *Explanation for* including only NRSI
- ☑ OR *Explanation for* including both RCTs and NRSI

- ☑ Yes
- ☐ No

4. Did the review authors use a comprehensive literature search strategy?

For Partial Yes (all the following):
- ☒ searched at least 2 databases (relevant to research question)
- ☑ provided key word and/or search strategy
- ☒ justified publication restrictions (eg, language)

For Yes, should also have (all the following):
- ☑ searched the reference lists/bibliographies of included studies
- ☐ searched trial/study registries
- ☐ included/consulted content experts in the field
- ☐ where relevant, searched for grey literature
- ☐ conducted search within 24 months of completion of the review

- ☐ Yes
- ☐ Partial Yes
- ☑ No

5. Did the review authors perform study selection in duplicate?

For Yes, either ONE of the following:
- ☒ at least two reviewers independently agreed on selection of eligible studies and achieved consensus on which studies to include
- ☐ OR two reviewers selected a sample of eligible studies and achieved good agreement (at least 80 per cent), with the remainder selected by one reviewer

- ☐ Yes
- ☑ No

6. Did the review authors perform data extraction in duplicate?

For Yes, either ONE of the following:
- ☑ at least two reviewers achieved consensus on which data to extract

- ☑ Yes

from included studies
☐ OR two reviewers extracted data from a sample of eligible studies and achieved good agreement (at least 80 per cent), with the remainder extracted by one reviewer

☐ No

7. Did the review authors provide a list of excluded studies and justify the exclusions?

For Partial Yes:	For Yes, must also have:	
☑ provided a list of all potentially relevant studies that were read in full text form but excluded from the review	☑ Justified the exclusion from the review of each potentially relevant study	☑ Yes<br>☐ Partial Yes<br>☐ No

8. Did the review authors describe the included studies in adequate detail?

For Partial Yes (ALL the following):	For Yes, should also have ALL the following:	
☑ described populations<br>☑ described interventions<br>☒ described comparators<br>☑ described outcomes<br>☑ described research designs	☒ described population in detail<br>☐ described intervention and comparator in detail (including doses where relevant)<br>☐ described study's setting<br>☐ timeframe for follow-up	☐ Yes<br>☐ Partial Yes<br>☑ No

9. Did the review authors use a satisfactory technique for assessing the risk of bias (RoB) in individual studies that were included in the review?

**RCTs**<br>For Partial Yes, must have assessed RoB from	For Yes, must also have assessed RoB from:	
☑ unconcealed allocation, *and*<br>☑ lack of blinding of patients and assessors when assessing outcomes (unnecessary for objective outcomes such as all cause mortality)	☑ allocation sequence that was not truly random, *and*<br>☒ selection of the reported result from among multiple measurements or analyses of a specified outcome	☐ Yes<br>☑ Partial Yes<br>☐ No<br>☐ Includes only NRSI
**NRSI**<br>For Partial Yes, must have assessed RoB:	For Yes, must also have assessed RoB:	
☐ from confounding, *and*<br>☐ from selection bias	☐ methods used to ascertain exposures and outcomes, *and*<br>☐ selection of the reported result from among multiple measurements or analyses of a specified outcome	☐ Yes<br>☐ Partial Yes<br>☐ No<br>☑ Includes only RCTs

10. Did the review authors report on the sources of funding for the studies included in the review?

For Yes	
☒ Must have reported on the sources of funding for individual studies included in the review. Note: Reporting that the reviewers looked for this information but it was not reported by study authors also qualifies	☐ Yes<br>☑ No

11. If meta-analysis was performed did the review authors use appropriate methods for statistical combination of results?

**RCTs**<br>For Yes:	
☑ The authors justified combining the data in a meta-analysis<br>☑ AND they used an appropriate weighted technique to combine study results and adjusted for heterogeneity if present	☐ Yes<br>☑ No<br>☐ No meta-analysis

☒ AND investigated the causes of any heterogeneity	conducted
**For NRSI**<br>For Yes:<br>☐ The authors justified combining the data in a meta-analysis<br>☐ AND they used an appropriate weighted technique to combine study results, adjusting for heterogeneity if present<br>☐ AND they statistically combined effect estimates from NRSI that were adjusted for confounding, rather than combining raw data, or justified combining raw data when adjusted effect estimates were not available<br>☐ AND they reported separate summary estimates for RCTs and NRSI separately when both were included in the review	☐ Yes<br>☐ No<br>☐ No meta-analysis conducted
**12. If meta-analysis was performed, did the review authors assess the potential impact of RoB in individual studies on the results of the meta-analysis or other evidence synthesis?**	
For Yes:<br>☒ included only low risk of bias RCTs<br>☐ OR, if the pooled estimate was based on RCTs and/or NRSI at variable RoB, the authors performed analyses to investigate possible impact of RoB on summary estimates of effect	☐ Yes<br>☑ No<br>☐ No meta-analysis conducted
**13. Did the review authors account for RoB in individual studies when interpreting/discussing the results of the review?**	
For Yes:<br>☒ included only low risk of bias RCTs<br>☐ OR, if RCTs with moderate or high RoB, or NRSI were included the review provided a discussion of the likely impact of RoB on the results	☐ Yes<br>☑ No
**14. Did the review authors provide a satisfactory explanation for, and discussion of, any heterogeneity observed in the results of the review?**	
For Yes:<br>☐ There was no significant heterogeneity in the results<br>☒ OR if heterogeneity was present the authors performed an investigation of sources of any heterogeneity in the results and discussed the impact of this on the results of the review	☐ Yes<br>☑ No
**15. If they performed quantitative synthesis did the review authors carry out an adequate investigation of publication bias (small study bias) and discuss its likely impact on the results of the review?**	
For Yes:<br>☑ performed graphical or statistical tests for publication bias and discussed the likelihood and magnitude of impact of publication bias	☑ Yes<br>☐ No<br>☐ No meta-analysis conducted
**16. Did the review authors report any potential sources of conflict of interest, including any funding they received for conducting the review?**	
For Yes:<br>☑ The authors reported no competing interests OR<br>☐ The authors described their funding sources and how they managed potential conflicts of interest	☑ Yes<br>☐ No

High-intensity interval training for cardiometabolic health in adults with metabolic syndrome: a systematic review and meta-analysis of randomised controlled trials
(Poon et al., 2024)

AMSTAR 2 **(Rating: Moderate)**

1. Did the research questions and inclusion criteria for the review include the components of PICO?

For Yes:
- ☑ Population
- ☑ Intervention
- ☑ Comparator group
- ☑ Outcome

Optional (recommended)
- ☑ Timeframe for follow-up

- ☑ Yes
- ☐ No

2. Did the report of the review contain an explicit statement that the review methods were established prior to the conduct of the review and did the report justify any significant deviations from the protocol?

For Partial Yes:
The authors state that they had a written protocol or guide that included ALL the following:
- ☑ review question(s)
- ☑ a search strategy
- ☑ inclusion/exclusion criteria
- ☑ a risk of bias assessment

For Yes:
As for partial yes, plus the protocol should be registered and should also have specified:
- ☑ a meta-analysis/synthesis plan, if appropriate, *and*
- ☑ a plan for investigating causes of heterogeneity
- ☑ justification for any deviations from the protocol

- ☑ Yes
- ☐ Partial Yes
- ☐ No

3. Did the review authors explain their selection of the study designs for inclusion in the review?

For Yes, the review should satisfy ONE of the following:
- ☑ *Explanation for* including only RCTs
- ☐ OR *Explanation for* including only NRSI
- ☐ OR *Explanation for* including both RCTs and NRSI

- ☑ Yes
- ☐ No

4. Did the review authors use a comprehensive literature search strategy?

For Partial Yes (all the following):
- ☑ searched at least 2 databases (relevant to research question)
- ☑ provided key word and/or search strategy
- ☑ justified publication restrictions (eg, language)

For Yes, should also have (all the following):
- ☑ searched the reference lists/bibliographies of included studies
- ☑ searched trial/study registries
- ☐ included/consulted content experts in the field
- ☒ where relevant, searched for grey literature
- ☑ conducted search within 24 months of completion of the review

- ☐ Yes
- ☑ Partial Yes
- ☐ No

5. Did the review authors perform study selection in duplicate?

For Yes, either ONE of the following:
- ☑ at least two reviewers independently agreed on selection of eligible studies and achieved consensus on which studies to include
- ☐ OR two reviewers selected a sample of eligible studies and achieved good agreement (at least 80 per cent), with the remainder selected by one reviewer

- ☑ Yes
- ☐ No

6. Did the review authors perform data extraction in duplicate?

For Yes, either ONE of the following:
- ☑ at least two reviewers achieved consensus on which data to extract

- ☑ Yes

from included studies
☐ OR two reviewers extracted data from a sample of eligible studies and achieved good agreement (at least 80 per cent), with the remainder extracted by one reviewer

☐ No

7. Did the review authors provide a list of excluded studies and justify the exclusions?

For Partial Yes:	For Yes, must also have:	
☒ provided a list of all potentially relevant studies that were read in full text form but excluded from the review	☐ Justified the exclusion from the review of each potentially relevant study	☐ Yes<br>☐ Partial Yes<br>☑ No

8. Did the review authors describe the included studies in adequate detail?

For Partial Yes (ALL the following):	For Yes, should also have ALL the following:	
☑ described populations<br>☑ described interventions<br>☑ described comparators<br>☑ described outcomes<br>☑ described research designs	☑ described population in detail<br>☑ described intervention and comparator in detail (including doses where relevant)<br>☑ described study's setting<br>☑ timeframe for follow-up	☑ Yes<br>☐ Partial Yes<br>☐ No

9. Did the review authors use a satisfactory technique for assessing the risk of bias (RoB) in individual studies that were included in the review?

RCTs

For Partial Yes, must have assessed RoB from	For Yes, must also have assessed RoB from:	
☑ unconcealed allocation, *and*<br>☑ lack of blinding of patients and assessors when assessing outcomes (unnecessary for objective outcomes such as all cause mortality)	☑ allocation sequence that was not truly random, *and*<br>☑ selection of the reported result from among multiple measurements or analyses of a specified outcome	☑ Yes<br>☐ Partial Yes<br>☐ No<br>☐ Includes only NRSI

NRSI

For Partial Yes, must have assessed RoB:	For Yes, must also have assessed RoB:	
☐ from confounding, *and*<br>☐ from selection bias	☐ methods used to ascertain exposures and outcomes, *and*<br>☐ selection of the reported result from among multiple measurements or analyses of a specified outcome	☐ Yes<br>☐ Partial Yes<br>☐ No<br>☑ Includes only RCTs

10. Did the review authors report on the sources of funding for the studies included in the review?

For Yes	
☒ Must have reported on the sources of funding for individual studies included in the review. Note: Reporting that the reviewers looked for this information but it was not reported by study authors also qualifies	☐ Yes<br>☑ No

11. If meta-analysis was performed did the review authors use appropriate methods for statistical combination of results?

RCTs

For Yes:	
☑ The authors justified combining the data in a meta-analysis<br>☑ AND they used an appropriate weighted technique to combine study results and adjusted for heterogeneity if present	☑ Yes<br>☐ No<br>☐ No meta-analysis

☑ AND investigated the causes of any heterogeneity	conducted
**For NRSI**<br>For Yes:<br>☐ The authors justified combining the data in a meta-analysis<br>☐ AND they used an appropriate weighted technique to combine study results, adjusting for heterogeneity if present<br>☐ AND they statistically combined effect estimates from NRSI that were adjusted for confounding, rather than combining raw data, or justified combining raw data when adjusted effect estimates were not available<br>☐ AND they reported separate summary estimates for RCTs and NRSI separately when both were included in the review	☐ Yes<br>☐ No<br>☐ No meta-analysis conducted
**12. If meta-analysis was performed, did the review authors assess the potential impact of RoB in individual studies on the results of the meta-analysis or other evidence synthesis?**	
For Yes:<br>☐ included only low risk of bias RCTs<br>☑ OR, if the pooled estimate was based on RCTs and/or NRSI at variable RoB, the authors performed analyses to investigate possible impact of RoB on summary estimates of effect	☑ Yes<br>☐ No<br>☐ No meta-analysis conducted
**13. Did the review authors account for RoB in individual studies when interpreting/discussing the results of the review?**	
For Yes:<br>☐ included only low risk of bias RCTs<br>☑ OR, if RCTs with moderate or high RoB, or NRSI were included the review provided a discussion of the likely impact of RoB on the results	☑ Yes<br>☐ No
**14. Did the review authors provide a satisfactory explanation for, and discussion of, any heterogeneity observed in the results of the review?**	
For Yes:<br>☐ There was no significant heterogeneity in the results<br>☑ OR if heterogeneity was present the authors performed an investigation of sources of any heterogeneity in the results and discussed the impact of this on the results of the review	☑ Yes<br>☐ No
**15. If they performed quantitative synthesis did the review authors carry out an adequate investigation of publication bias (small study bias) and discuss its likely impact on the results of the review?**	
For Yes:<br>☑ performed graphical or statistical tests for publication bias and discussed the likelihood and magnitude of impact of publication bias	☑ Yes<br>☐ No<br>☐ No meta-analysis conducted
**16. Did the review authors report any potential sources of conflict of interest, including any funding they received for conducting the review?**	
For Yes:<br>☑ The authors reported no competing interests OR<br>☐ The authors described their funding sources and how they managed potential conflicts of interest	☑ Yes<br>☐ No

High intensity interval training exerciseinduced physiological changes and their potential influence on metabolic syndrome clinical biomarkers: a meta-analysis
(Serrablo et al., 2020)

AMSTAR 2 **(Rating: Critically Low)**

1. Did the research questions and inclusion criteria for the review include the components of PICO?

For Yes:
- ☑ Population
- ☑ Intervention
- ☑ Comparator group
- ☑ Outcome

Optional (recommended)
- ☑ Timeframe for follow-up

- ☑ Yes
- ☐ No

2. Did the report of the review contain an explicit statement that the review methods were established prior to the conduct of the review and did the report justify any significant deviations from the protocol?

For Partial Yes:
The authors state that they had a written protocol or guide that included ALL the following:
- ☒ review question(s)
- ☐ a search strategy
- ☐ inclusion/exclusion criteria
- ☐ a risk of bias assessment

For Yes:
As for partial yes, plus the protocol should be registered and should also have specified:
- ☐ a meta-analysis/synthesis plan, if appropriate, *and*
- ☐ a plan for investigating causes of heterogeneity
- ☐ justification for any deviations from the protocol

- ☐ Yes
- ☐ Partial Yes
- ☑ No

3. Did the review authors explain their selection of the study designs for inclusion in the review?

For Yes, the review should satisfy ONE of the following:
- ☑ *Explanation for* including only RCTs
- ☐ OR *Explanation for* including only NRSI
- ☐ OR *Explanation for* including both RCTs and NRSI

- ☑ Yes
- ☐ No

4. Did the review authors use a comprehensive literature search strategy?

For Partial Yes (all the following):
- ☑ searched at least 2 databases (relevant to research question)
- ☑ provided key word and/or search strategy
- ☑ justified publication restrictions (eg, language)

For Yes, should also have (all the following):
- ☑ searched the reference lists/bibliographies of included studies
- ☒ searched trial/study registries
- ☐ included/consulted content experts in the field
- ☐ where relevant, searched for grey literature
- ☐ conducted search within 24 months of completion of the review

- ☐ Yes
- ☑ Partial Yes
- ☐ No

5. Did the review authors perform study selection in duplicate?

For Yes, either ONE of the following:
- ☑ at least two reviewers independently agreed on selection of eligible studies and achieved consensus on which studies to include
- ☐ OR two reviewers selected a sample of eligible studies and achieved good agreement (at least 80 per cent), with the remainder selected by one reviewer

- ☑ Yes
- ☐ No

6. Did the review authors perform data extraction in duplicate?

For Yes, either ONE of the following:
- ☒ at least two reviewers achieved consensus on which data to extract

- ☐ Yes

from included studies
☐ OR two reviewers extracted data from a sample of eligible studies and achieved good agreement (at least 80 per cent), with the remainder extracted by one reviewer

☑ No

7. Did the review authors provide a list of excluded studies and justify the exclusions?

For Partial Yes:
☒ provided a list of all potentially relevant studies that were read in full text form but excluded from the review

For Yes, must also have:
☐ Justified the exclusion from the review of each potentially relevant study

☐ Yes
☐ Partial Yes
☑ No

8. Did the review authors describe the included studies in adequate detail?

For Partial Yes (ALL the following):
☑ described populations
☑ described interventions
☑ described comparators
☑ described outcomes
☑ described research designs

For Yes, should also have ALL the following:
☑ described population in detail
☑ described intervention and comparator in detail (including doses where relevant)
☑ described study's setting
☑ timeframe for follow-up

☑ Yes
☐ Partial Yes
☐ No

9. Did the review authors use a satisfactory technique for assessing the risk of bias (RoB) in individual studies that were included in the review?

RCTs

For Partial Yes, must have assessed RoB from
☑ unconcealed allocation, *and*
☑ lack of blinding of patients and assessors when assessing outcomes (unnecessary for objective outcomes such as all cause mortality)

For Yes, must also have assessed RoB from:
☑ allocation sequence that was not truly random, *and*
☒ selection of the reported result from among multiple measurements or analyses of a specified outcome

☐ Yes
☑ Partial Yes
☐ No
☐ Includes only NRSI

NRSI

For Partial Yes, must have assessed RoB:
☐ from confounding, *and*
☐ from selection bias

For Yes, must also have assessed RoB:
☐ methods used to ascertain exposures and outcomes, *and*
☐ selection of the reported result from among multiple measurements or analyses of a specified outcome

☐ Yes
☐ Partial Yes
☐ No
☑ Includes only RCTs

10. Did the review authors report on the sources of funding for the studies included in the review?

For Yes
☒ Must have reported on the sources of funding for individual studies included in the review. Note: Reporting that the reviewers looked for this information but it was not reported by study authors also qualifies

☐ Yes
☑ No

11. If meta-analysis was performed did the review authors use appropriate methods for statistical combination of results?

RCTs

For Yes:
☑ The authors justified combining the data in a meta-analysis
☒ AND they used an appropriate weighted technique to combine study results and adjusted for heterogeneity if present

☐ Yes
☑ No
☐ No meta-analysis

☐ AND investigated the causes of any heterogeneity — conducted

For NRSI

For Yes:

- ☐ The authors justified combining the data in a meta-analysis
  - ☐ AND they used an appropriate weighted technique to combine study results, adjusting for heterogeneity if present
  - ☐ AND they statistically combined effect estimates from NRSI that were adjusted for confounding, rather than combining raw data, or justified combining raw data when adjusted effect estimates were not available
  - ☐ AND they reported separate summary estimates for RCTs and NRSI separately when both were included in the review

☐ Yes
☐ No
☐ No meta-analysis conducted

12. If meta-analysis was performed, did the review authors assess the potential impact of RoB in individual studies on the results of the meta-analysis or other evidence synthesis?

For Yes:

- ☒ included only low risk of bias RCTs
- ☐ OR, if the pooled estimate was based on RCTs and/or NRSI at variable RoB, the authors performed analyses to investigate possible impact of RoB on summary estimates of effect

☐ Yes
☑ No
☐ No meta-analysis conducted

13. Did the review authors account for RoB in individual studies when interpreting/discussing the results of the review?

For Yes:

- ☒ included only low risk of bias RCTs
- ☐ OR, if RCTs with moderate or high RoB, or NRSI were included the review provided a discussion of the likely impact of RoB on the results

☐ Yes
☑ No

14. Did the review authors provide a satisfactory explanation for, and discussion of, any heterogeneity observed in the results of the review?

For Yes:

- ☐ There was no significant heterogeneity in the results
- ☒ OR if heterogeneity was present the authors performed an investigation of sources of any heterogeneity in the results and discussed the impact of this on the results of the review

☐ Yes
☑ No

15. If they performed quantitative synthesis did the review authors carry out an adequate investigation of publication bias (small study bias) and discuss its likely impact on the results of the review?

For Yes:

- ☒ performed graphical or statistical tests for publication bias and discussed the likelihood and magnitude of impact of publication bias

☐ Yes
☑ No
☐ No meta-analysis conducted

16. Did the review authors report any potential sources of conflict of interest, including any funding they received for conducting the review?

For Yes:

- ☐ The authors reported no competing interests OR
- ☑ The authors described their funding sources and how they managed potential conflicts of interest

☑ Yes
☐ No

Effects of qigong exercise on cardiovascular risk factors in patients with metabolic syndrome: A systematic review and meta-analysis
(Tao et al., 2023)

AMSTAR 2 **(Rating: Critically Low)**

1. Did the research questions and inclusion criteria for the review include the components of PICO?

For Yes:
- ☑ Population
- ☑ Intervention
- ☑ Comparator group
- ☑ Outcome

Optional (recommended)
- ☐ Timeframe for follow-up

- ☑ Yes
- ☐ No

2. Did the report of the review contain an explicit statement that the review methods were established prior to the conduct of the review and did the report justify any significant deviations from the protocol?

For Partial Yes:
The authors state that they had a written protocol or guide that included ALL the following:
- ☒ review question(s)
- ☐ a search strategy
- ☐ inclusion/exclusion criteria
- ☐ a risk of bias assessment

For Yes:
As for partial yes, plus the protocol should be registered and should also have specified:
- ☒ a meta-analysis/synthesis plan, if appropriate, *and*
- ☐ a plan for investigating causes of heterogeneity
- ☐ justification for any deviations from the protocol

- ☐ Yes
- ☐ Partial Yes
- ☑ No

3. Did the review authors explain their selection of the study designs for inclusion in the review?

For Yes, the review should satisfy ONE of the following:
- ☑ *Explanation for* including only RCTs
- ☐ OR *Explanation for* including only NRSI
- ☐ OR *Explanation for* including both RCTs and NRSI

- ☑ Yes
- ☐ No

4. Did the review authors use a comprehensive literature search strategy?

For Partial Yes (all the following):
- ☑ searched at least 2 databases (relevant to research question)
- ☑ provided key word and/or search strategy
- ☒ justified publication restrictions (eg, language)

For Yes, should also have (all the following):
- ☒ searched the reference lists/bibliographies of included studies
- ☐ searched trial/study registries
- ☐ included/consulted content experts in the field
- ☐ where relevant, searched for grey literature
- ☐ conducted search within 24 months of completion of the review

- ☐ Yes
- ☐ Partial Yes
- ☑ No

5. Did the review authors perform study selection in duplicate?

For Yes, either ONE of the following:
- ☒ at least two reviewers independently agreed on selection of eligible studies and achieved consensus on which studies to include
- ☐ OR two reviewers selected a sample of eligible studies and achieved good agreement (at least 80 per cent), with the remainder selected by one reviewer

- ☐ Yes
- ☑ No

6. Did the review authors perform data extraction in duplicate?

For Yes, either ONE of the following:
- ☒ at least two reviewers achieved consensus on which data to extract

- ☐ Yes

from included studies<br>☐ OR two reviewers extracted data from a sample of eligible studies <u>and</u> achieved good agreement (at least 80 per cent), with the remainder extracted by one reviewer		☑ No

7. Did the review authors provide a list of excluded studies and justify the exclusions?

For Partial Yes:	For Yes, must also have:	
☒ provided a list of all potentially relevant studies that were read in full text form but excluded from the review	☐ Justified the exclusion from the review of each potentially relevant study	☐ Yes<br>☐ Partial Yes<br>☑ No

8. Did the review authors describe the included studies in adequate detail?

For Partial Yes (ALL the following):	For Yes, should also have ALL the following:	
☑ described populations<br>☑ described interventions<br>☑ described comparators<br>☑ described outcomes<br>☑ described research designs	☑ described population in detail<br>☑ described intervention and comparator in detail (including doses where relevant)<br>☑ described study's setting<br>☑ timeframe for follow-up	☑ Yes<br>☐ Partial Yes<br>☐ No

9. Did the review authors use a satisfactory technique for assessing the risk of bias (RoB) in individual studies that were included in the review?

**RCTs**<br>For Partial Yes, must have assessed RoB from	For Yes, must also have assessed RoB from:	
☑ unconcealed allocation, *and*<br>☑ lack of blinding of patients and assessors when assessing outcomes (unnecessary for objective outcomes such as all cause mortality)	☑ allocation sequence that was not truly random, *and*<br>☑ selection of the reported result from among multiple measurements or analyses of a specified outcome	☑ Yes<br>☐ Partial Yes<br>☐ No<br>☐ Includes only NRSI

**NRSI**<br>For Partial Yes, must have assessed RoB:	For Yes, must also have assessed RoB:	
☐ from confounding, *and*<br>☐ from selection bias	☐ methods used to ascertain exposures and outcomes, *and*<br>☐ selection of the reported result from among multiple measurements or analyses of a specified outcome	☐ Yes<br>☐ Partial Yes<br>☐ No<br>☑ Includes only RCTs

10. Did the review authors report on the sources of funding for the studies included in the review?

For Yes	
☒ Must have reported on the sources of funding for individual studies included in the review. Note: Reporting that the reviewers looked for this information but it was not reported by study authors also qualifies	☐ Yes<br>☑ No

11. If meta-analysis was performed did the review authors use appropriate methods for statistical combination of results?

**RCTs**<br>For Yes:	
☑ The authors justified combining the data in a meta-analysis<br>☑ AND they used an appropriate weighted technique to combine study results and adjusted for heterogeneity if present	☑ Yes<br>☐ No<br>☐ No meta-analysis

☑ AND investigated the causes of any heterogeneity	conducted
**For NRSI**<br>For Yes:<br>☐ The authors justified combining the data in a meta-analysis<br>☐ AND they used an appropriate weighted technique to combine study results, adjusting for heterogeneity if present<br>☐ AND they statistically combined effect estimates from NRSI that were adjusted for confounding, rather than combining raw data, or justified combining raw data when adjusted effect estimates were not available<br>☐ AND they reported separate summary estimates for RCTs and NRSI separately when both were included in the review	☐ Yes<br>☐ No<br>☐ No meta-analysis conducted

12. If meta-analysis was performed, did the review authors assess the potential impact of RoB in individual studies on the results of the meta-analysis or other evidence synthesis?

For Yes:<br>☑ included only low risk of bias RCTs<br>☐ OR, if the pooled estimate was based on RCTs and/or NRSI at variable RoB, the authors performed analyses to investigate possible impact of RoB on summary estimates of effect	☑ Yes<br>☐ No<br>☐ No meta-analysis conducted

13. Did the review authors account for RoB in individual studies when interpreting/discussing the results of the review?

For Yes:<br>☑ included only low risk of bias RCTs<br>☐ OR, if RCTs with moderate or high RoB, or NRSI were included the review provided a discussion of the likely impact of RoB on the results	☑ Yes<br>☐ No

14. Did the review authors provide a satisfactory explanation for, and discussion of, any heterogeneity observed in the results of the review?

For Yes:<br>☐ There was no significant heterogeneity in the results<br>☑ OR if heterogeneity was present the authors performed an investigation of sources of any heterogeneity in the results and discussed the impact of this on the results of the review	☑ Yes<br>☐ No

15. If they performed quantitative synthesis did the review authors carry out an adequate investigation of publication bias (small study bias) and discuss its likely impact on the results of the review?

For Yes:<br>☒ performed graphical or statistical tests for publication bias and discussed the likelihood and magnitude of impact of publication bias	☐ Yes<br>☑ No<br>☐ No meta-analysis conducted

16. Did the review authors report any potential sources of conflict of interest, including any funding they received for conducting the review?

For Yes:<br>☐ The authors reported no competing interests OR<br>☒ The authors described their funding sources and how they managed potential conflicts of interest	☐ Yes<br>☑ No

Aerobic, resistance or combined training: A systematic review and meta-analysis of exercise to reduce cardiovascular risk in adults with metabolic syndrome
(Wewege et al., 2018)

AMSTAR 2 **(Rating: Low)**

1. Did the research questions and inclusion criteria for the review include the components of PICO?

For Yes:	Optional (recommended)	
☑ Population<br>☑ Intervention<br>☑ Comparator group<br>☑ Outcome	☑ Timeframe for follow-up	☑ Yes<br>☐ No

2. Did the report of the review contain an explicit statement that the review methods were established prior to the conduct of the review and did the report justify any significant deviations from the protocol?

For Partial Yes:	For Yes:	
The authors state that they had a written protocol or guide that included ALL the following:<br>☑ review question(s)<br>☑ a search strategy<br>☑ inclusion/exclusion criteria<br>☑ a risk of bias assessment	As for partial yes, plus the protocol should be registered and should also have specified:<br>☑ a meta-analysis/synthesis plan, if appropriate, *and*<br>☑ a plan for investigating causes of heterogeneity<br>☑ justification for any deviations from the protocol	☑ Yes<br>☐ Partial Yes<br>☐ No

3. Did the review authors explain their selection of the study designs for inclusion in the review?

For Yes, the review should satisfy ONE of the following:	
☑ *Explanation for* including only RCTs<br>☐ OR *Explanation for* including only NRSI<br>☐ OR *Explanation for* including both RCTs and NRSI	☑ Yes<br>☐ No

4. Did the review authors use a comprehensive literature search strategy?

For Partial Yes (all the following):	For Yes, should also have (all the following):	
☑ searched at least 2 databases (relevant to research question)<br>☑ provided key word and/or search strategy<br>☑ justified publication restrictions (eg, language)	☒ searched the reference lists/bibliographies of included studies<br>☐ searched trial/study registries<br>☐ included/consulted content experts in the field<br>☐ where relevant, searched for grey literature<br>☐ conducted search within 24 months of completion of the review	☐ Yes<br>☑ Partial Yes<br>☐ No

5. Did the review authors perform study selection in duplicate?

For Yes, either ONE of the following:	
☑ at least two reviewers independently agreed on selection of eligible studies and achieved consensus on which studies to include<br>☐ OR two reviewers selected a sample of eligible studies and achieved good agreement (at least 80 per cent), with the remainder selected by one reviewer	☑ Yes<br>☐ No

6. Did the review authors perform data extraction in duplicate?

For Yes, either ONE of the following:	
☒ at least two reviewers achieved consensus on which data to extract	☐ Yes

from included studies

☐ OR two reviewers extracted data from a sample of eligible studies and achieved good agreement (at least 80 per cent), with the remainder extracted by one reviewer

☑ No

7. Did the review authors provide a list of excluded studies and justify the exclusions?

For Partial Yes:	For Yes, must also have:	
☑ provided a list of all potentially relevant studies that were read in full text form but excluded from the review	☑ Justified the exclusion from the review of each potentially relevant study	☑ Yes<br>☐ Partial Yes<br>☐ No

8. Did the review authors describe the included studies in adequate detail?

For Partial Yes (ALL the following):	For Yes, should also have ALL the following:	
☑ described populations<br>☑ described interventions<br>☒ described comparators<br>☒ described outcomes<br>☒ described research designs	☐ described population in detail<br>☐ described intervention and comparator in detail (including doses where relevant)<br>☐ described study's setting<br>☐ timeframe for follow-up	☐ Yes<br>☐ Partial Yes<br>☑ No

9. Did the review authors use a satisfactory technique for assessing the risk of bias (RoB) in individual studies that were included in the review?

RCTs

For Partial Yes, must have assessed RoB from	For Yes, must also have assessed RoB from:	
☑ unconcealed allocation, *and*<br>☑ lack of blinding of patients and assessors when assessing outcomes (unnecessary for objective outcomes such as all cause mortality)	☑ allocation sequence that was not truly random, *and*<br>☒ selection of the reported result from among multiple measurements or analyses of a specified outcome	☐ Yes<br>☑ Partial Yes<br>☐ No<br>☐ Includes only NRSI

NRSI

For Partial Yes, must have assessed RoB:	For Yes, must also have assessed RoB:	
☐ from confounding, *and*<br>☐ from selection bias	☐ methods used to ascertain exposures and outcomes, *and*<br>☐ selection of the reported result from among multiple measurements or analyses of a specified outcome	☐ Yes<br>☐ Partial Yes<br>☐ No<br>☑ Includes only RCTs

10. Did the review authors report on the sources of funding for the studies included in the review?

For Yes

☒ Must have reported on the sources of funding for individual studies included in the review. Note: Reporting that the reviewers looked for this information but it was not reported by study authors also qualifies

☐ Yes
☑ No

11. If meta-analysis was performed did the review authors use appropriate methods for statistical combination of results?

RCTs

For Yes:

☑ The authors justified combining the data in a meta-analysis

☑ AND they used an appropriate weighted technique to combine study results and adjusted for heterogeneity if present

☑ Yes
☐ No
☐ No meta-analysis

☑ AND investigated the causes of any heterogeneity	conducted
**For NRSI**<br>For Yes:<br>☐ The authors justified combining the data in a meta-analysis<br>☐ AND they used an appropriate weighted technique to combine study results, adjusting for heterogeneity if present<br>☐ AND they statistically combined effect estimates from NRSI that were adjusted for confounding, rather than combining raw data, or justified combining raw data when adjusted effect estimates were not available<br>☐ AND they reported separate summary estimates for RCTs and NRSI separately when both were included in the review	☐ Yes<br>☐ No<br>☐ No meta-analysis conducted
**12. If meta-analysis was performed, did the review authors assess the potential impact of RoB in individual studies on the results of the meta-analysis or other evidence synthesis?**	
For Yes:<br>☐ included only low risk of bias RCTs<br>☒ OR, if the pooled estimate was based on RCTs and/or NRSI at variable RoB, the authors performed analyses to investigate possible impact of RoB on summary estimates of effect	☐ Yes<br>☑ No<br>☐ No meta-analysis conducted
**13. Did the review authors account for RoB in individual studies when interpreting/discussing the results of the review?**	
For Yes:<br>☐ included only low risk of bias RCTs<br>☒ OR, if RCTs with moderate or high RoB, or NRSI were included the review provided a discussion of the likely impact of RoB on the results	☐ Yes<br>☑ No
**14. Did the review authors provide a satisfactory explanation for, and discussion of, any heterogeneity observed in the results of the review?**	
For Yes:<br>☑ There was no significant heterogeneity in the results<br>☐ OR if heterogeneity was present the authors performed an investigation of sources of any heterogeneity in the results and discussed the impact of this on the results of the review	☑ Yes<br>☐ No
**15. If they performed quantitative synthesis did the review authors carry out an adequate investigation of publication bias (small study bias) and discuss its likely impact on the results of the review?**	
For Yes:<br>☑ performed graphical or statistical tests for publication bias and discussed the likelihood and magnitude of impact of publication bias	☑ Yes<br>☐ No<br>☐ No meta-analysis conducted
**16. Did the review authors report any potential sources of conflict of interest, including any funding they received for conducting the review?**	
For Yes:<br>☑ The authors reported no competing interests OR<br>☐ The authors described their funding sources and how they managed potential conflicts of interest	☑ Yes<br>☐ No

Determining the effect size of aerobic exercise training on the standard lipid profile in sedentary adults with three or more metabolic syndrome factors: a systematic review and meta-analysis of randomised controlled trials
(Wood et al., 2021)

AMSTAR 2 **(Rating: Low)**

1. Did the research questions and inclusion criteria for the review include the components of PICO?

For Yes:
- ☑ Population
- ☑ Intervention
- ☑ Comparator group
- ☑ Outcome

Optional (recommended)
- ☐ Timeframe for follow-up

- ☑ Yes
- ☐ No

2. Did the report of the review contain an explicit statement that the review methods were established prior to the conduct of the review and did the report justify any significant deviations from the protocol?

For Partial Yes:
The authors state that they had a written protocol or guide that included ALL the following:
- ☑ review question(s)
- ☑ a search strategy
- ☑ inclusion/exclusion criteria
- ☑ a risk of bias assessment

For Yes:
As for partial yes, plus the protocol should be registered and should also have specified:
- ☑ a meta-analysis/synthesis plan, if appropriate, *and*
- ☑ a plan for investigating causes of heterogeneity
- ☑ justification for any deviations from the protocol

- ☑ Yes
- ☐ Partial Yes
- ☐ No

3. Did the review authors explain their selection of the study designs for inclusion in the review?

For Yes, the review should satisfy ONE of the following:
- ☑ *Explanation for* including only RCTs
- ☐ OR *Explanation for* including only NRSI
- ☐ OR *Explanation for* including both RCTs and NRSI

- ☑ Yes
- ☐ No

4. Did the review authors use a comprehensive literature search strategy?

For Partial Yes (all the following):
- ☑ searched at least 2 databases (relevant to research question)
- ☑ provided key word and/or search strategy
- ☑ justified publication restrictions (eg, language)

For Yes, should also have (all the following):
- ☑ searched the reference lists/bibliographies of included studies
- ☒ searched trial/study registries
- ☐ included/consulted content experts in the field
- ☐ where relevant, searched for grey literature
- ☐ conducted search within 24 months of completion of the review

- ☐ Yes
- ☑ Partial Yes
- ☐ No

5. Did the review authors perform study selection in duplicate?

For Yes, either ONE of the following:
- ☑ at least two reviewers independently agreed on selection of eligible studies and achieved consensus on which studies to include
- ☐ OR two reviewers selected a sample of eligible studies and achieved good agreement (at least 80 per cent), with the remainder selected by one reviewer

- ☑ Yes
- ☐ No

6. Did the review authors perform data extraction in duplicate?

For Yes, either ONE of the following:
- ☑ at least two reviewers achieved consensus on which data to extract

- ☑ Yes

from included studies
☐ OR two reviewers extracted data from a sample of eligible studies and achieved good agreement (at least 80 per cent), with the remainder extracted by one reviewer

☐ No

7. Did the review authors provide a list of excluded studies and justify the exclusions?

For Partial Yes:	For Yes, must also have:	
☒ provided a list of all potentially relevant studies that were read in full text form but excluded from the review	☐ Justified the exclusion from the review of each potentially relevant study	☐ Yes<br>☐ Partial Yes<br>☑ No

8. Did the review authors describe the included studies in adequate detail?

For Partial Yes (ALL the following):	For Yes, should also have ALL the following:	
☑ described populations<br>☑ described interventions<br>☑ described comparators<br>☒ described outcomes<br>☐ described research designs	☐ described population in detail<br>☒ described intervention and comparator in detail (including doses where relevant)<br>☐ described study's setting<br>☐ timeframe for follow-up	☐ Yes<br>☐ Partial Yes<br>☑ No

9. Did the review authors use a satisfactory technique for assessing the risk of bias (RoB) in individual studies that were included in the review?

**RCTs**<br>For Partial Yes, must have assessed RoB from<br>☑ unconcealed allocation, *and*<br>☑ lack of blinding of patients and assessors when assessing outcomes (unnecessary for objective outcomes such as all cause mortality)	For Yes, must also have assessed RoB from:<br>☑ allocation sequence that was not truly random, *and*<br>☑ selection of the reported result from among multiple measurements or analyses of a specified outcome	☑ Yes<br>☐ Partial Yes<br>☐ No<br>☐ Includes only NRSI
**NRSI**<br>For Partial Yes, must have assessed RoB:<br>☐ from confounding, *and*<br>☐ from selection bias	For Yes, must also have assessed RoB:<br>☐ methods used to ascertain exposures and outcomes, *and*<br>☐ selection of the reported result from among multiple measurements or analyses of a specified outcome	☐ Yes<br>☐ Partial Yes<br>☐ No<br>☑ Includes only RCTs

10. Did the review authors report on the sources of funding for the studies included in the review?

For Yes	
☒ Must have reported on the sources of funding for individual studies included in the review. Note: Reporting that the reviewers looked for this information but it was not reported by study authors also qualifies	☐ Yes<br>☑ No

11. If meta-analysis was performed did the review authors use appropriate methods for statistical combination of results?

**RCTs**<br>For Yes:<br>☑ The authors justified combining the data in a meta-analysis<br>☑ AND they used an appropriate weighted technique to combine study results and adjusted for heterogeneity if present	☑ Yes<br>☐ No<br>☐ No meta-analysis

☑ AND investigated the causes of any heterogeneity	conducted
**For NRSI**<br>For Yes:<br>☐ The authors justified combining the data in a meta-analysis<br>☐ AND they used an appropriate weighted technique to combine study results, adjusting for heterogeneity if present<br>☐ AND they statistically combined effect estimates from NRSI that were adjusted for confounding, rather than combining raw data, or justified combining raw data when adjusted effect estimates were not available<br>☐ AND they reported separate summary estimates for RCTs and NRSI separately when both were included in the review	☐ Yes<br>☐ No<br>☐ No meta-analysis conducted

12. If meta-analysis was performed, did the review authors assess the potential impact of RoB in individual studies on the results of the meta-analysis or other evidence synthesis?

For Yes:<br>☐ included only low risk of bias RCTs<br>☑ OR, if the pooled estimate was based on RCTs and/or NRSI at variable RoB, the authors performed analyses to investigate possible impact of RoB on summary estimates of effect	☑ Yes<br>☐ No<br>☐ No meta-analysis conducted

13. Did the review authors account for RoB in individual studies when interpreting/discussing the results of the review?

For Yes:<br>☐ included only low risk of bias RCTs<br>☑ OR, if RCTs with moderate or high RoB, or NRSI were included the review provided a discussion of the likely impact of RoB on the results	☑ Yes<br>☐ No

14. Did the review authors provide a satisfactory explanation for, and discussion of, any heterogeneity observed in the results of the review?

For Yes:<br>☐ There was no significant heterogeneity in the results<br>☑ OR if heterogeneity was present the authors performed an investigation of sources of any heterogeneity in the results and discussed the impact of this on the results of the review	☑ Yes<br>☐ No

15. If they performed quantitative synthesis did the review authors carry out an adequate investigation of publication bias (small study bias) and discuss its likely impact on the results of the review?

For Yes:<br>☒ performed graphical or statistical tests for publication bias and discussed the likelihood and magnitude of impact of publication bias	☐ Yes<br>☑ No<br>☐ No meta-analysis conducted

16. Did the review authors report any potential sources of conflict of interest, including any funding they received for conducting the review?

For Yes:<br>☑ The authors reported no competing interests OR<br>☐ The authors described their funding sources and how they managed potential conflicts of interest	☑ Yes<br>☐ No

Is low-volume high-intensity interval training a time-efficient strategy to improve cardiometabolic health and body composition? A meta-analysis
(Yin et al., 2024)

AMSTAR 2 **(Rating: Low)**

1. Did the research questions and inclusion criteria for the review include the components of PICO?

For Yes:
- ☑ Population
- ☑ Intervention
- ☑ Comparator group
- ☑ Outcome

Optional (recommended)
- ☑ Timeframe for follow-up

- ☑ Yes
- ☐ No

2. Did the report of the review contain an explicit statement that the review methods were established prior to the conduct of the review and did the report justify any significant deviations from the protocol?

For Partial Yes:
The authors state that they had a written protocol or guide that included ALL the following:
- ☑ review question(s)
- ☑ a search strategy
- ☑ inclusion/exclusion criteria
- ☑ a risk of bias assessment

For Yes:
As for partial yes, plus the protocol should be registered and should also have specified:
- ☑ a meta-analysis/synthesis plan, if appropriate, *and*
- ☑ a plan for investigating causes of heterogeneity
- ☒ justification for any deviations from the protocol

- ☐ Yes
- ☑ Partial Yes
- ☐ No

3. Did the review authors explain their selection of the study designs for inclusion in the review?

For Yes, the review should satisfy ONE of the following:
- ☑ *Explanation for* including only RCTs
- ☐ OR *Explanation for* including only NRSI
- ☐ OR *Explanation for* including both RCTs and NRSI

- ☑ Yes
- ☐ No

4. Did the review authors use a comprehensive literature search strategy?

For Partial Yes (all the following):
- ☑ searched at least 2 databases (relevant to research question)
- ☑ provided key word and/or search strategy
- ☑ justified publication restrictions (eg, language)

For Yes, should also have (all the following):
- ☑ searched the reference lists/bibliographies of included studies
- ☒ searched trial/study registries
- ☐ included/consulted content experts in the field
- ☐ where relevant, searched for grey literature
- ☐ conducted search within 24 months of completion of the review

- ☐ Yes
- ☑ Partial Yes
- ☐ No

5. Did the review authors perform study selection in duplicate?

For Yes, either ONE of the following:
- ☒ at least two reviewers independently agreed on selection of eligible studies and achieved consensus on which studies to include
- ☐ OR two reviewers selected a sample of eligible studies and achieved good agreement (at least 80 per cent), with the remainder selected by one reviewer

- ☐ Yes
- ☑ No

6. Did the review authors perform data extraction in duplicate?

For Yes, either ONE of the following:
- ☑ at least two reviewers achieved consensus on which data to extract

- ☑ Yes

from included studies
☐ OR two reviewers extracted data from a sample of eligible studies and achieved good agreement (at least 80 per cent), with the remainder extracted by one reviewer

☐ No

7. Did the review authors provide a list of excluded studies and justify the exclusions?

For Partial Yes:
☒ provided a list of all potentially relevant studies that were read in full text form but excluded from the review

For Yes, must also have:
☐ Justified the exclusion from the review of each potentially relevant study

☐ Yes
☐ Partial Yes
☑ No

8. Did the review authors describe the included studies in adequate detail?

For Partial Yes (ALL the following):
☑ described populations
☑ described interventions
☑ described comparators
☒ described outcomes
☑ described research designs

For Yes, should also have ALL the following:
☑ described population in detail
☑ described intervention and comparator in detail (including doses where relevant)
☒ described study's setting
☑ timeframe for follow-up

☐ Yes
☐ Partial Yes
☑ No

9. Did the review authors use a satisfactory technique for assessing the risk of bias (RoB) in individual studies that were included in the review?

RCTs
For Partial Yes, must have assessed RoB from
☑ unconcealed allocation, *and*
☑ lack of blinding of patients and assessors when assessing outcomes (unnecessary for objective outcomes such as all cause mortality)

For Yes, must also have assessed RoB from:
☑ allocation sequence that was not truly random, *and*
☒ selection of the reported result from among multiple measurements or analyses of a specified outcome

☐ Yes
☑ Partial Yes
☐ No
☐ Includes only NRSI

NRSI
For Partial Yes, must have assessed RoB:
☐ from confounding, *and*
☐ from selection bias

For Yes, must also have assessed RoB:
☐ methods used to ascertain exposures and outcomes, *and*
☐ selection of the reported result from among multiple measurements or analyses of a specified outcome

☐ Yes
☐ Partial Yes
☐ No
☑ Includes only RCTs

10. Did the review authors report on the sources of funding for the studies included in the review?

For Yes
☒ Must have reported on the sources of funding for individual studies included in the review. Note: Reporting that the reviewers looked for this information but it was not reported by study authors also qualifies

☐ Yes
☑ No

11. If meta-analysis was performed did the review authors use appropriate methods for statistical combination of results?

RCTs
For Yes:
☑ The authors justified combining the data in a meta-analysis
☑ AND they used an appropriate weighted technique to combine study results and adjusted for heterogeneity if present

☑ Yes
☐ No
☐ No meta-analysis

☑ AND investigated the causes of any heterogeneity	conducted
**For NRSI**<br>For Yes:<br>☐ The authors justified combining the data in a meta-analysis<br>☐ AND they used an appropriate weighted technique to combine study results, adjusting for heterogeneity if present<br>☐ AND they statistically combined effect estimates from NRSI that were adjusted for confounding, rather than combining raw data, or justified combining raw data when adjusted effect estimates were not available<br>☐ AND they reported separate summary estimates for RCTs and NRSI separately when both were included in the review	☐ Yes<br>☐ No<br>☐ No meta-analysis conducted
**12. If meta-analysis was performed, did the review authors assess the potential impact of RoB in individual studies on the results of the meta-analysis or other evidence synthesis?**	
For Yes:<br>☐ included only low risk of bias RCTs<br>☒ OR, if the pooled estimate was based on RCTs and/or NRSI at variable RoB, the authors performed analyses to investigate possible impact of RoB on summary estimates of effect	☐ Yes<br>☑ No<br>☐ No meta-analysis conducted
**13. Did the review authors account for RoB in individual studies when interpreting/discussing the results of the review?**	
For Yes:<br>☐ included only low risk of bias RCTs<br>☒ OR, if RCTs with moderate or high RoB, or NRSI were included the review provided a discussion of the likely impact of RoB on the results	☐ Yes<br>☑ No
**14. Did the review authors provide a satisfactory explanation for, and discussion of, any heterogeneity observed in the results of the review?**	
For Yes:<br>☐ There was no significant heterogeneity in the results<br>☑ OR if heterogeneity was present the authors performed an investigation of sources of any heterogeneity in the results and discussed the impact of this on the results of the review	☑ Yes<br>☐ No
**15. If they performed quantitative synthesis did the review authors carry out an adequate investigation of publication bias (small study bias) and discuss its likely impact on the results of the review?**	
For Yes:<br>☑ performed graphical or statistical tests for publication bias and discussed the likelihood and magnitude of impact of publication bias	☑ Yes<br>☐ No<br>☐ No meta-analysis conducted
**16. Did the review authors report any potential sources of conflict of interest, including any funding they received for conducting the review?**	
For Yes:<br>☑ The authors reported no competing interests OR<br>☐ The authors described their funding sources and how they managed potential conflicts of interest	☑ Yes<br>☐ No

Supplementary Table 6 Summary of risk of bias, methodological quality, certainty of evidence, sensitivity analysis, publication bias, and potential moderators on PA outcomes reported by each included systematic review

Reference	Main findings
Cramer et al 2016	**Risk of bias**<br>• Cochrane risk of bias showed that one study had reported adequate random sequence generation and allocation concealment, and one further study had reported adequate allocation concealment but not random sequence generation.<br>• One study had a low risk of detection bias and three had a low risk of attrition bias.<br>**Publication bias**<br>• As fewer than 10 studies were included in each meta-analysis, funnel plots could not be analysed<br>**Sensitivity analyses**<br>• A low risk of selection bias and a low risk of detection bias each only appeared in one study.<br>• No effects remained significant if only this study was included in the analysis.<br>• In studies with a low risk of attrition bias, the effect of yoga compared to usual care on systolic blood pressure did not change substantially<br>**Potential moderators**<br>• As all studies included yoga postures, subgroup analyses could not be performed
Leme et al 2018	**Certainty of evidence**<br>• On the basis of the GRADE system, pooled data of all meta-analyses were classified as low-quality evidence.<br>• All variables were downgraded one level owing to the presence of risk of bias and one level owing to the presence of publication bias<br>**Methodological quality**<br>• PEDro scale indicated that three studies were considered ''high quality,'' thirteen studies were considered "moderate quality,'' and one study was considered "poor quality.''<br>**Potential moderators**<br>Potential influences of the duration and type of AT training, that is, short-term vs. long-term trials and continuous vs. interval training, were not detected as comparisons of subgroups revealed no differences in pooled estimates.
Leme et al 2016	**Certainty of evidence**<br>• On the basis of the GRADE system , pooled data of HDL-cholesterol, triglycerides, systolic blood pressure, diastolic blood pressure and waist circumference were classified as moderate-quality evidence.<br>• All these variables were downgraded one level owing to the presence of risk of bias<br>• The evidence for fasting plasma glucose was classified as low-quality evidence<br>**Methodological quality**<br>• PEDro scale indicated that one study was considered 'high quality', three studies were considered 'moderate quality' and four studies were considered 'poor quality'.<br>**Potential moderators**<br>• Long-term trials (≥6 months) are more effective than short-term trials in reducing systolic blood pressure.<br>• There was also a reduction in diastolic blood pressure, although this was not statistically significant (p=0.07)<br>• Other potential influences of these aspects were not detected
Li et al 2024	**Risk of bias**<br>• One study had unclear bias in the randomization process, and all studies were rated as "Some concerns" for bias in the implementation of the

	predefined interventions.<br>• No studies showed bias related to outcome data missing, outcome measurement bias, or selective reporting bias.<br>**Publication bias**<br>• The results of the Begg test suggested that there was no publication bias in the study.<br>**Sensitivity analysis**<br>• The results of the meta-analysis for each outcome indicator appear to be stable.<br>**Potential moderators**<br>• Subgroup analysis showed that the intervention program with exercise **form of fitness qigong, exercise cycle of 24-48 weeks**, and **exercise frequency of 6-7 times/week** could significantly improve each risk factor.
Ostman et al 2017	**Methodological quality**<br>• Median TESTex score was 9 out of 15.<br>• One study scored 5, two studies scored 6, four studies scored 7, four scored 8, four scored 9, nine scored 10, three scored 11, one scored 12 and one scored 13.<br>**Publication bias**<br>• Funnel (Egger) plots of the primary analyses showed mild to moderate evidence of publication bias as more than one-third of the studies fell outside the funnel plot border.<br>**Potential moderators**<br>• Neither weekly nor total program exercise time significantly affected outcome measures.<br>• The inclusion of participants with T2DM who also fit diagnostic criteria for MetS did not significantly affect any of the outcome measures.
Pattyn et al 2013	**Methodological quality**<br>• The median PEDro score was 6 (out of 9), with a range from 5 to 8.<br>**Publication bias**<br>• Funnel plots did not show any significant publication bias for all of the primary and secondary outcomes.<br>**Sensitivity analysis**<br>• When omitted the only trial in which all patients had T2DM, overall changes in DBP, HDL-C and WC were no longer statistically significant.
Poon et al 2024	**Risk of bias**<br>• Seven studies displayed a 'high' overall RoB, 15 studies showed 'some concerns', and only one study displayed a 'low' risk of overall bias<br>**Publication bias**<br>• Funnel plots for all analyses showed no indication of publication bias<br>**Sensitivity analysis**<br>• Sensitivity analyses using the leave-one-out method did not reveal substantial impact of individual studies on overall results for HIIT versus non-ac tive control.<br>**Potential moderators**<br>• Subgroup analyses indicated that improvements were observed irrespective of protocol volume or intervention duration for most outcome measures, except that **low-volume HIIT protocols and shorter-duration interventions tended to result in greater improvements in TC, HDL-C, LDL-C and TG as compared with interventions with high-volume protocols and longer durations**
Serrablo et al 2020	**Methodological quality**<br>• The mean score obtained with the PEDro quality scale was 6.7 (minimum score being 5, and the maximum score being 7, higher scores indicating better quality).<br>**Risk of bias**

	• According to the Cochrane risk of bias tool, most items (60%) were classified as low risk of bias.
Tao et al 2023	**Risk of bias**<br>• The quality of the included studies was scored according to the Cochrane risk of bias assessment tool using six evaluation criteria<br>• The quality of the included articles in this study was mainly between 4 and 4.5 points, indicating a low risk of research bias<br>**Sensitivity analysis**<br>• The sensitivity analysis was performed by excluding individual studies one-by-one<br>• The results showed no significant changes between the combined results of the remaining studies and the meta-analysis results, indicating that the original analysis results were not affected by specific studies<br>**Potential moderators**<br>• For BMI and the HDL-C and TC levels, the longer the intervention duration, the better the effect in general<br>• However, for the FBG level, the 3-month intervention seemed more effective.<br>• In addition, for the LDL-C level, Qigong exercise had no obvious improvement effect.
Wewege et al 2018	**Methodological quality**<br>• PEDro scores showed that the majority of studies were only of moderate quality; no studies were considered high quality.<br>**Publication bias**<br>• Publication bias was not detected in any analysis.<br>**Potential moderators**<br>• Sub-analyses suggested that aerobic exercise progressed to vigorous intensity, and conducted 3 days/week for 12 weeks offered larger and more widespread improvements<br>• Females exhibited statistically significant improvements to 5 MetS risk factors, compared to 2 for males.<br>• Participants >50 years experienced significant improvements in more MetS factors than <50 years.
Wood et al 2021	**Methodological quality**<br>• A median TESTEX score of 10 (from maximum score of 15;<br>range 7–15) was derived.<br>• Within-study risk of bias was mainly low or medium.<br>**Publication bias**<br>• Potential small study effects for the SLP were trivial and did not invalidate the univariate meta-analysis<br>**Potential moderators**<br>• The change in LDL-C was approximately 50% associated with AET volume<br>• The AET intensity covariate explained approximately 50% of the change in TRG<br>• AET volume was principally responsible for the change in HDL-C<br>• Pooled RCT data indicated AET programmes of moderate or higher intensity with a minimum 12-week duration significantly improved the standard lipid profile in sedentary adults diagnosed with MetS but otherwise healthy.
Yin et al 2024	**Methodological quality**<br>• The obtained PEDro scores ranged from moderate to high quality (5 to 9) for the systematic review and meta-analysis
